# Supplementary material for: Coverage and quality of DNA barcode references for Central and Northern European Odonata
Source: PeerJ. 2021 May 3;9:e11192. doi: 10.7717/peerj.11192 (PMC8101477; doi:10.7717/peerj.11192)
Supplement: Supplemental Information 1 — All project data is available under the publicly accessible DOI: dx.doi.org/10.5883/DS-ODOGER [file peerj-09-11192-s001.pdf]

GenBank #	Process ID	Sample ID	BIN	Seq. Length	Collection Date	Identification	Life Stage	Institution (short)	Identifier	country; GPS
HM422047	FBAQU308-09	BC ZSM AQU 00308	BOLD:AAJ5779	658[0n]	19-Aug-2009	*Aeshna affinis*	adult	SNSB-ZSM	M. Schoen	Germany; 48.366, 11.875
MW490495	PLSW064-20	ODOPL_198	BOLD:AAJ5779	658[0n]	25-Aug-2019	*Aeshna affinis*	adult	Lodz	Grzegorz Tonczyk	Poland; 51.757, 19.4
MW490196	PLSW065-20	ODOPL_199	BOLD:AAJ5779	658[0n]	25-Aug-2019	*Aeshna affinis*	adult	Lodz	Grzegorz Tonczyk	Poland; 51.757, 19.4
MW490475	RODI021-20	ROAE-AA-1	BOLD:AAJ5779	658[0n]	18-Jul-2017	*Aeshna affinis*	adult	Babes-Bolyai	Beata Nagy	Romania; 47.843, 23.27
MW490399	RODI022-20	ROAE-AA-2	BOLD:AAJ5779	658[0n]	18-Jul-2017	*Aeshna affinis*	adult	Babes-Bolyai	Beata Nagy	Romania; 47.843, 23.27
MW490232	EDF016-18	Odon416	BOLD:AAA6531	639[0n]	23-Aug-2018	*Aeshna caerulea*	adult	NHMW	Lukas Zangl	Austria; 47.288, 13.659
MW490272	EDF017-18	Odon417	BOLD:AAA6531	658[0n]	23-Aug-2018	*Aeshna caerulea*	adult	NHMW	Lukas Zangl	Austria; 47.291, 13.66
MW490172	TRDOD058-14	TRD-ODO76	BOLD:AAA6531	658[0n]	08-Sep-2010	*Aeshna caerulea*	adult	NTNU	Jon K. Skei	Norway; 63.325, 10.652
MW490352	TRDOD067-14	TRD-ODO85	BOLD:AAA6531	658[0n]	03-Aug-2010	*Aeshna caerulea*	adult	NTNU	Jon K. Skei	Norway; 63.498, 11.23
MW490283	TRDOD036-14	TRD-ODO54	BOLD:AAA6531	658[0n]	19-Jul-2014	*Aeshna caerulea*	adult	NTNU	Jon K. Skei	Norway; 63.543, 11.033
MW490109	GBMIX941-14	GBOL10043	BOLD:ACI1053	658[0n]	23-Jul-2014	*Aeshna cyanea*		SNSB-ZSM	Emmanuel FA Toussaint	France; 48.639, 0.085
MW490148	GBODO010-18	GBOL 20133	BOLD:ACI1053	658[6n]	27-Jul-2018	*Aeshna cyanea*	adult	SNSB-ZSM	Stefan Koch	Germany; 47.9229, 11.5085
MW490364	GBMIX3763-18	GBOL19831	BOLD:ACI1053	663[2n]	24-Sep-2017	*Aeshna cyanea*	adult	SNSB-ZSM	Lars Hendrich	Germany; 48.0216, 10.3358
MW490245	GBMIX2219-15	GBOL13668	BOLD:ACI1053	658[0n]	26-Aug-2013	*Aeshna cyanea*	adult	SNSB-ZSM	Jerome Moriniere	Germany; 50.4707, 7.22338
MW490372	PLSW129-20	ODOPL_227	BOLD:ACI1053	658[0n]	30-Jun-2019	*Aeshna cyanea*	larvae	Lodz	Grzegorz Tonczyk	Poland; 54.612, 18.51
MW490429	PLSW130-20	ODOPL_228	BOLD:ACI1053	658[0n]	30-Jun-2019	*Aeshna cyanea*	larvae	Lodz	Grzegorz Tonczyk	Poland; 54.612, 18.51
MW490539	PLSW131-20	ODOPL_229	BOLD:ACI1053	658[0n]	30-Jun-2019	*Aeshna cyanea*	larvae	Lodz	Grzegorz Tonczyk	Poland; 54.612, 18.51
HM901857	FBAQU478-10	BC ZSM AQU 00383	BOLD:AAJ5811	619[0n]	16-Jul-2009	*Aeshna grandis*	adult	SNSB-ZSM	S. V. Ober	Germany; 47.908, 11.523
MW490538	GBODO066-18	GBOL 20189	BOLD:AAJ5811	658[1n]	04-Jul-2018	*Aeshna grandis*	adult	SNSB-ZSM	Stefan Koch	Germany; 48.3305, 11.8047
MW490355	ZMBN005-15	Kurs2015-MH5	BOLD:AAJ5811	658[0n]	19-Aug-2015	*Aeshna grandis*	adult	Bergen	Steffen Roth	Norway; 60.333, 5.359
MW490265	TRDOD038-14	TRD-ODO56	BOLD:AAJ5811	658[0n]	22-Jul-2014	*Aeshna grandis*	adult	NTNU	Jon K. Skei	Norway; 63.548, 10.996
MW490517	TRDOD039-14	TRD-ODO57	BOLD:AAJ5811	658[0n]	22-Jul-2014	*Aeshna grandis*	adult	NTNU	Jon K. Skei	Norway; 63.548, 10.996
MW490273	TRDOD040-14	TRD-ODO58	BOLD:AAJ5811	658[0n]	22-Jul-2014	*Aeshna grandis*	adult	NTNU	Jon K. Skei	Norway; 63.548, 10.996
MW490130	TRDOD033-14	TRD-ODO51	BOLD:AAJ5811	658[0n]	14-Jul-2014	*Aeshna grandis*	adult	NTNU	Jon K. Skei	Norway; 63.653, 11.093
MW490309	ODOPL064-19	OdoPL64	BOLD:AAJ5811	637[0n]	02-Nov-2018	*Aeshna grandis*	larvae	Lodz	Grzegorz Tonczyk	Poland; 51.3338, 19.8961
MW490417	ODOPL066-19	OdoPL66	BOLD:AAJ5811	635[0n]	02-Nov-2018	*Aeshna grandis*	larvae	Lodz	Grzegorz Tonczyk	Poland; 51.3338, 19.8961
MW490237	EDF001-18	NOaS1-2019_Odo0029	BOLD:ADC2941	658[0n]	31-May-2017	*Aeshna isoceles*	adult	NHMW	Iris Fischer	Austria; ,
MW490521	EDF002-18	NOaS1-2019_Odo0053	BOLD:ADC2941	658[0n]	01-Jun-2017	*Aeshna isoceles*	adult	NHMW	Iris Fischer	Austria; ,
MW490288	GBODO007-18	GBOL 20130	BOLD:ADC2941	658[0n]	21-May-2018	*Aeshna isoceles*	adult	SNSB-ZSM	Stefan Koch	Germany; 47.7561, 11.3698
MW490325	GBODO143-18	GBOL 20266	BOLD:ADC2941	658[0n]	21-May-2018	*Aeshna isoceles*	adult	SNSB-ZSM	Stefan Koch	Germany; 47.7561, 11.3698
MW490370	PLSW077-20	ODOPL_211	BOLD:ADC2941	654[0n]	02-Jun-2019	*Aeshna isoceles*	adult	Lodz	Grzegorz Tonczyk	Poland; 51.551, 19.338
MW490312	GBEPT2003-14	GBOL09395	BOLD:AAJ1281	658[0n]	02-Jul-2014	*Aeshna juncea*	larvae	SNSB-ZSM	Sofia Wiedenbrug	Germany; 47.506, 12.967
MW490104	FBAQU1431-13	GBOL00275	BOLD:AAJ1281	620[0n]	23-Aug-2011	*Aeshna juncea*	adult	SNSB-ZSM	Stefan Koch	Germany; 47.6291, 11.1893
MW490419	GBODO013-18	GBOL 20136	BOLD:AAJ1281	530[1n]	27-Jul-2018	*Aeshna juncea*	adult	SNSB-ZSM	Stefan Koch	Germany; 47.8252, 11.3012
MW490477	GBODO009-18	GBOL 20132	BOLD:AAJ1281	658[4n]	27-Jul-2018	*Aeshna juncea*	adult	SNSB-ZSM	Stefan Koch	Germany; 47.9229, 11.5085
HM901858	FBAQU479-10	BC ZSM AQU 00384	BOLD:AAJ1281	633[0n]	27-Jul-2009	*Aeshna juncea*	adult	SNSB-ZSM	S. V. Ober	Germany; 48.107, 11.458
MW490396	TRDOD034-14	TRD-ODO52	BOLD:AAJ1281	609[0n]	12-Jul-2014	*Aeshna juncea*	adult	NTNU	Jon K. Skei	Norway; 63.541, 11.033
MW490188	TRDOD035-14	TRD-ODO53	BOLD:AAJ1281	658[0n]	12-Jul-2014	*Aeshna juncea*	adult	NTNU	Jon K. Skei	Norway; 63.541, 11.033
MW490305	TRDOD041-14	TRD-ODO59	BOLD:AAJ1281	658[0n]	19-Jul-2014	*Aeshna juncea*	adult	NTNU	Jon K. Skei	Norway; 63.541, 11.033
MW490448	TRDOD037-14	TRD-ODO55	BOLD:AAJ1281	658[0n]	22-Jul-2014	*Aeshna juncea*	adult	NTNU	Jon K. Skei	Norway; 63.548, 10.996
HM901884	FBAQU522-10	BC ZSM AQU 00427	BOLD:AAJ5810	628[0n]	09-Jan-2009	*Aeshna mixta*	adult	SNSB-ZSM	F. Weihrauch	Germany; 47.895, 12.167

GenBank #	Process ID	Sample ID	BIN	Seq. Length	Collection Date	Identification	Life Stage	Institution (short)	Identifier	country; GPS
MW490236	FBAQU480-10	BC ZSM AQU 00385	BOLD:AAJ5810	554[0n]	24-Aug-2009	*Aeshna mixta*	adult	SNSB-ZSM	S. V. Ober	Germany; 48.177, 11.384
MW490375	GODO046-19	ZFMK-TIS-2616217	BOLD:AAJ5810	658[0n]	23-Oct-2018	*Aeshna mixta*	adult	Udo Rothe	Udo Rothe	Italy; 41.0228, 9.42699
MW490185	GODO045-19	ZFMK-TIS-2616216	BOLD:AAJ5810	658[0n]	22-Oct-2018	*Aeshna mixta*	adult	Udo Rothe	Udo Rothe	Italy; 41.1237, 9.3061
KU180298	GBMIN88537-17	KU180298	BOLD:ABZ5296	603[0n]		*Aeshna subarctica*		GenBank, NCBI	0	Germany; ,
MW490481	GBODO043-18	GBOL 20166	BOLD:ABZ5296	658[0n]	05-Sep-2018	*Aeshna subarctica*	adult	SNSB-ZSM	Stefan Koch	Germany; 47.6668, 11.0506
MW490450	GBODO045-18	GBOL 20168	BOLD:ABZ5296	658[2n]	05-Sep-2018	*Aeshna subarctica*	adult	SNSB-ZSM	Stefan Koch	Germany; 47.6668, 11.0506
MW490403	GBODO048-18	GBOL 20171	BOLD:ABZ5296	658[0n]	05-Sep-2018	*Aeshna subarctica*	adult	SNSB-ZSM	Stefan Koch	Germany; 47.6668, 11.0506
HM901883	FBAQU521-10	BC ZSM AQU 00426	BOLD:ABZ5296	625[0n]	16-Jul-2009	*Aeshna subarctica*	adult	SNSB-ZSM	F. Weihrauch	Germany; 47.895, 12.167
MW490090	TRDOD069-14	TRD-ODO87	BOLD:ABZ5296	658[0n]	25-Aug-2014	*Aeshna subarctica*	adult	NTNU	Jon K. Skei	Norway; 63.355, 10.824
MW490291	TRDOD062-14	TRD-ODO80	BOLD:ABZ5296	658[0n]	15-Aug-2009	*Aeshna subarctica*	adult	NTNU	Jon K. Skei	Norway; 63.395, 11.806
MW490195	TRDOD050-14	TRD-ODO68	BOLD:ABZ5296	658[0n]	02-Aug-2014	*Aeshna subarctica*	adult	NTNU	Jon K. Skei	Norway; 63.543, 11.033
MW490221	TRDOD056-14	TRD-ODO74	BOLD:ABZ5296	658[0n]	11-Aug-2014	*Aeshna subarctica*	adult	NTNU	Jon K. Skei	Norway; 63.555, 11.099
KU180300	GBMIN88540-17	KU180300	BOLD:ADC2700	603[0n]		*Aeshna viridis*		GenBank, NCBI	0	Germany; ,
KU180301	GBMIN88539-17	KU180301	BOLD:ADC2700	603[3n]		*Aeshna viridis*		GenBank, NCBI	0	Germany; ,
MT298251	ZPLOD042-20	MIB:ZPL:07842	BOLD:ACH7840	658[0n]		*Anax ephippiger*	adult	Milano Bicocca	Giacomo Assandri	Italy; 44.4, 8.8
MT298252	ZPLOD044-20	MIB:ZPL:07844	BOLD:ACH7840	658[0n]		*Anax ephippiger*	adult	Giacomo Assandri	Giacomo Assandri	Italy; 45.2, 8.7
MW490464	GBODO126-18	GBOL 20249	BOLD:ABX6596	658[0n]	27-Jun-2018	*Anax imperator*	adult	SNSB-ZSM	Stefan Koch	Germany; 47.7852, 11.3619
MW490466	GBODO128-18	GBOL 20251	BOLD:ABX6596	658[0n]	27-Jun-2018	*Anax imperator*	adult	SNSB-ZSM	Stefan Koch	Germany; 47.7852, 11.3619
MW490140	GBODO012-18	GBOL 20135	BOLD:ABX6596	658[1n]	27-Jul-2018	*Anax imperator*	adult	SNSB-ZSM	Stefan Koch	Germany; 47.9229, 11.5085
HM901859	FBAQU481-10	BC ZSM AQU 00386	BOLD:ABX6596	611[0n]	18-Jun-2009	*Anax imperator*	adult	SNSB-ZSM	S. V. Ober	Germany; 48.236, 11.505
MW490125	GBODO094-18	GBOL 20217	BOLD:ABX6596	657[0n]	04-Jul-2018	*Anax imperator*	adult	SNSB-ZSM	Stefan Koch	Germany; 48.3115, 11.9189
MW490299	GBODO082-18	GBOL 20205	BOLD:ABX6596	514[1n]	04-Jul-2018	*Anax imperator*	adult	SNSB-ZSM	Stefan Koch	Germany; 48.3305, 11.8047
MN731357	ODOPL085-19	OdoPL85	BOLD:ABX6596	637[0n]	02-Nov-2018	*Anax imperator*	larvae	Lodz	Grzegorz Tonczyk	Poland; 51.3164, 19.9037
MN731359	ODOPL118-19	OdoPL118	BOLD:ABX6596	637[0n]	17-Nov-2018	*Anax imperator*	larvae	Lodz	Grzegorz Tonczyk	Poland; 51.5799, 19.2154
MN731358	ODOPL119-19	OdoPL119	BOLD:ABX6596	636[0n]	17-Nov-2018	*Anax imperator*	larvae	Lodz	Grzegorz Tonczyk	Poland; 51.5799, 19.2154
MW490509	PLSW120-20	ODOPL_225	BOLD:ABX6596	658[0n]	10-Apr-2019	*Anax imperator*	larvae	Lodz	Grzegorz Tonczyk	Poland; 51.58, 19.215
MN731356	ODOPL025-19	OdoPL25	BOLD:ABX6596	634[0n]	14-Oct-2018	*Anax imperator*	larvae	Lodz	Grzegorz Tonczyk	Poland; 51.5814, 19.2188
MW490490	PLSW054-20	ODOPL_188	BOLD:ABX6596	658[0n]	09-Jun-2019	*Anax imperator*	adult	Lodz	Grzegorz Tonczyk	Poland; 51.758, 19.407
MW490451	EDF019-19	Odon200	BOLD:ABX6596	658[0n]	05-Jun-2018	*Anax parthenope*	adult	NHMW	Stephan Koblmueller	Austria; 47.76, 16.808
MN701501	ODOPL132-19	ODOPL132	BOLD:ABX6596	671[0n]	10-Jun-2019	*Anax parthenope*	adult	Lodz	Grzegorz Tonczyk	Poland; 54.0263, 23.0665
MN701506	ODOPL133-19	ODOPL133	BOLD:ABX6596	671[0n]	10-Jun-2019	*Anax parthenope*	adult	Lodz	Grzegorz Tonczyk	Poland; 54.0263, 23.0665
MN701514	ODOPL134-19	ODOPL134	BOLD:ABX6596	603[0n]	10-Jun-2019	*Anax parthenope*	adult	Lodz	Grzegorz Tonczyk	Poland; 54.0286, 23.0527
MW490145	GODO005-18	ZFMK-TIS-2561818	BOLD:ABX6596	658[0n]	11-Aug-2015	*Anax sp.*	larvae	ZFMK	0	Hungary; 47.6787, 16.8479
MW490368	GODO006-18	ZFMK-TIS-2561820	BOLD:ABX6596	658[0n]	11-Aug-2015	*Anax sp.*	larvae	ZFMK	0	Hungary; 47.6787, 16.8479
MW490313	GODO039-18	ZFMK-TIS-2554164	BOLD:ADN2163	658[0n]	16-Jun-2011	*Boyeria irene*		ZFMK	Armin W. Lorenz	France; ,
MT298270	ZPLOD075-20	MIB:ZPL:07875	BOLD:ABA9406	658[0n]		*Brachythemis impartita*	adult	Milano Bicocca	Giacomo Assandri	Italy; 37.5, 14.9
MT298271	ZPLOD073-20	MIB:ZPL:07873	BOLD:ABA9406	658[0n]		*Brachythemis impartita*	adult	Giacomo Assandri	Giacomo Assandri	Italy; 39.9, 8.5
MT298269	ZPLOD072-20	MIB:ZPL:07872	BOLD:ABA9406	658[0n]		*Brachythemis impartita*	adult	Giacomo Assandri	Giacomo Assandri	Italy; 40.7, 9
MW490174	GBEPT945-14	GBOL05515	BOLD:ACI1765	658[0n]	13-Jun-2014	*Brachytron pratense*	adult	SNSB-ZSM	Michael Franzen	Austria; 47.931, 16.746
MW490212	GBEPT946-14	GBOL05516	BOLD:ACI1765	658[0n]	13-Jun-2014	*Brachytron pratense*	adult	SNSB-ZSM	Michael Franzen	Austria; 47.931, 16.746
MW490502	GBUPS282-14	GBOL02932	BOLD:ACI1765	610[2n]		*Brachytron pratense*	adult	SNSB-ZSM	Lars Hendrich	Germany; ,

GenBank #	Process ID	Sample ID	BIN	Seq. Length	Collection Date	Identification	Life Stage	Institution (short)	Identifier	country; GPS
MW490323	GBODO142-18	GBOL 20265	BOLD:ACI1765	658[0n]	21-May-2018	*Brachytron pratense*	adult	SNSB-ZSM	Stefan Koch	Germany; 47.7561, 11.3698
MW490259	GBODO149-18	GBOL 20272	BOLD:ACI1765	658[0n]	21-May-2018	*Brachytron pratense*	adult	SNSB-ZSM	Stefan Koch	Germany; 47.7561, 11.3698
MW490168	PLSW135-20	ODOPL_230	BOLD:ACI1765	658[0n]	30-Jun-2019	*Brachytron pratense*	larvae	Lodz	Grzegorz Tonczyk	Poland; 54.626, 18.489
MW490290	PLSW136-20	ODOPL_231	BOLD:ACI1765	658[0n]	30-Jun-2019	*Brachytron pratense*	larvae	Lodz	Grzegorz Tonczyk	Poland; 54.626, 18.489
MW490216	PLSW137-20	ODOPL_232	BOLD:ACI1765	658[0n]	30-Jun-2019	*Brachytron pratense*	larvae	Lodz	Grzegorz Tonczyk	Poland; 54.626, 18.489
MT298276	ZPLOD815-20	MIB:ZPL:08615	BOLD:ADC2822	658[0n]		*Caliaeschna microstigma*	adult	Giacomo Assandri	Giacomo Assandri	Montenegro; 42.2, 19.1
MW490203	GODO040-19	ZFMK-TIS-2616197	BOLD:ADV2208	658[0n]	23-Oct-2018	*Calopteryx haemorrhoidalis*	adult	Udo Rothe	Udo Rothe	Italy; 41.0228, 9.42699
MW490230	GODO018-18	ZFMK-TIS-2008755	BOLD:ADC4648	658[0n]		*Calopteryx splendens*		ZFMK	Sabine Schmidt-Halewicz	Germany; 47.6587, 9.16629
MW490332	GODO019-18	ZFMK-TIS-2008756	BOLD:ADC4648	658[0n]		*Calopteryx splendens*		ZFMK	Sabine Schmidt-Halewicz	Germany; 47.6587, 9.16629
MW490432	GODO020-18	ZFMK-TIS-2595144	BOLD:ADC4648	658[0n]		*Calopteryx splendens*	larvae	ZFMK	Sabine Schmidt-Halewicz	Germany; 49.4121, 11.0182
MW490134	GODO022-18	ZFMK-TIS-2010621	BOLD:ADC4648	658[0n]	30-May-2017	*Calopteryx splendens*	adult	ZFMK	T. Sellmeier	Germany; 50.777, 7.365
MW490510	GODO024-18	ZFMK-TIS-2612900	BOLD:ADC4648	658[0n]	30-May-2017	*Calopteryx splendens*	adult	ZFMK	T. Sellmeier	Germany; 50.777, 7.365
MW490124	GODO025-18	ZFMK-TIS-2612901	BOLD:ADC4648	658[0n]	30-May-2017	*Calopteryx splendens*	adult	ZFMK	T. Sellmeier	Germany; 50.777, 7.365
MW490324	GODO032-18	ZFMK-TIS-2010640	BOLD:ADC4648	658[0n]	11-Jul-2017	*Calopteryx splendens*	adult	ZFMK	T. Sellmeier	Germany; 50.8, 7.585
MW490199	GODO033-18	ZFMK-TIS-2612907	BOLD:ADC4648	658[0n]	27-Jun-2017	*Calopteryx splendens*	adult	ZFMK	T. Sellmeier	Germany; 50.8, 7.585
MW490487	GODO034-18	ZFMK-TIS-2612908	BOLD:ADC4648	658[0n]	27-Jun-2017	*Calopteryx splendens*	adult	ZFMK	T. Sellmeier	Germany; 50.8, 7.585
MW490143	ODOPL056-19	OdoPL56	BOLD:ADC4648	634[0n]	11-Oct-2018	*Calopteryx splendens*	larvae	Lodz	Grzegorz Tonczyk	Poland; 50.4118, 18.1075
MW490204	ODOPL057-19	OdoPL57	BOLD:ADC4648	636[0n]	11-Oct-2018	*Calopteryx splendens*	larvae	Lodz	Grzegorz Tonczyk	Poland; 50.4118, 18.1075
MW490397	ODOPL042-19	OdoPL42	BOLD:ADC4648	635[0n]	10-Oct-2018	*Calopteryx splendens*	larvae	Lodz	Grzegorz Tonczyk	Poland; 51.6184, 16.333
MW490099	ODOPL043-19	OdoPL43	BOLD:ADC4648	638[0n]	10-Oct-2018	*Calopteryx splendens*	larvae	Lodz	Grzegorz Tonczyk	Poland; 51.6184, 16.333
MW490111	ODOPL033-19	OdoPL33	BOLD:ADC4648	638[0n]	10-Oct-2018	*Calopteryx splendens*	larvae	Lodz	Grzegorz Tonczyk	Poland; 52.0552, 15.4302
MW490329	ODOPL034-19	OdoPL34	BOLD:ADC4648	634[0n]	10-Oct-2018	*Calopteryx splendens*	larvae	Lodz	Grzegorz Tonczyk	Poland; 52.0552, 15.4302
MW490433	ODOPL012-18	OdoPL12	BOLD:ADC4648	626[0n]	05-Oct-2018	*Calopteryx splendens*	larvae	Lodz	Grzegorz Tonczyk	Poland; 52.3855, 20.1954
HM901860	FBAQU484-10	BC ZSM AQU 00389	BOLD:AAE7398	601[0n]	29-Jul-2009	*Calopteryx virgo*	adult	SNSB-ZSM	S. V. Ober	Germany; 47.765, 12.816
HM901885	FBAQU526-10	BC ZSM AQU 00431	BOLD:AAE7398	543[1n]	09-May-2009	*Calopteryx virgo*	larvae	SNSB-ZSM	Monika Hess	Germany; 49.128, 11.522
MW459503	GODO001-18	ZFMK-TIS-2532522	BOLD:AAE7398	658[0n]	22-Jun-2014	*Calopteryx virgo*		ZFMK	Guido Haas	Germany; 50.8, 7.17
MW490428	PLSW039-20	ODOPL_173	BOLD:AAE7398	658[0n]	13-Jun-2019	*Calopteryx virgo*	adult	Lodz	Grzegorz Tonczyk	Poland; 54.097, 23.063
MT298297	ZPLOD124-20	MIB:ZPL:07924	BOLD:ADC4648	658[0n]		*Calopteryx xanthostoma*	adult	Milano Bicocca	Giacomo Assandri	Italy; 44.1, 8.2
MT298296	ZPLOD125-20	MIB:ZPL:07925	BOLD:ADC4648	658[0n]		*Calopteryx xanthostoma*	adult	Milano Bicocca	Giacomo Assandri	Italy; 44.1, 8.2
MT298299	ZPLOD130-20	MIB:ZPL:07930	BOLD:ACH6070	658[0n]		*Ceriagrion tenellum*	adult	Milano Bicocca	Giacomo Assandri	Italy; 37.1, 15.3
MT298303	ZPLOD134-20	MIB:ZPL:07934	BOLD:ACH6070	658[0n]		*Ceriagrion tenellum*	adult	Milano Bicocca	Giacomo Assandri	Italy; 40.2, 18.4
MT298301	ZPLOD127-20	MIB:ZPL:07927	BOLD:ACH6070	658[0n]		*Ceriagrion tenellum*	adult	Milano Bicocca	Giacomo Assandri	Italy; 40.4, 8.6
MT298304	ZPLOD140-20	MIB:ZPL:07940	BOLD:ACH6070	658[0n]		*Ceriagrion tenellum*	adult	Milano Bicocca	Giacomo Assandri	Italy; 41.5, 13.1
MT298298	ZPLOD138-20	MIB:ZPL:07938	BOLD:ACH6070	658[0n]		*Ceriagrion tenellum*	adult	Milano Bicocca	Giacomo Assandri	Italy; 44.5, 7.7
MT298302	ZPLOD135-20	MIB:ZPL:07935	BOLD:ACH6070	658[0n]		*Ceriagrion tenellum*	adult	Milano Bicocca	Giacomo Assandri	Italy; 45.8, 9.2
MT298300	ZPLOD129-20	MIB:ZPL:07929	BOLD:ACH6070	658[0n]		*Ceriagrion tenellum*	adult	Milano Bicocca	Giacomo Assandri	Italy; 45.9, 13.2
MW490121	EDF018-18	Odon454	BOLD:ADR7794	658[0n]	18-Sep-2018	*Chalcolestes parvidens*	adult	NHMW	Lukas Zangl	Austria; 47.71, 16.887
MT298308	ZPLOD141-20	MIB:ZPL:07941	BOLD:ADR7794	658[0n]		*Chalcolestes parvidens*	adult	Milano Bicocca	Giacomo Assandri	Italy; 42.6, 12.2
MT298309	ZPLOD147-20	MIB:ZPL:07947	BOLD:ADR7794	658[0n]		*Chalcolestes parvidens*	adult	Milano Bicocca	Giacomo Assandri	Italy; 44.6, 11.1
MT298310	ZPLOD142-20	MIB:ZPL:07942	BOLD:ADR7794	658[0n]		*Chalcolestes parvidens*	adult	Milano Bicocca	Giacomo Assandri	Italy; 45.6, 13.8
MT298311	ZPLOD143-20	MIB:ZPL:07943	BOLD:ADR7794	658[0n]		*Chalcolestes parvidens*	adult	Milano Bicocca	Giacomo Assandri	Italy; 45.8, 13.6

GenBank #	Process ID	Sample ID	BIN	Seq. Length	Collection Date	Identification	Life Stage	Institution (short)	Identifier	country; GPS
GU682188	FBAQU201-09	BC ZSM AQU 00201	BOLD:AAI7225	655[0n]	31-Jul-2009	*Chalcolestes viridis*	larvae	SNSB-ZSM	Monika Hess	Germany; 47.731, 11.312
MW490416	GBCOU7598-14	GBOL10430	BOLD:AAI7225	658[0n]	23-Jul-2014	*Chalcolestes viridis*	adult	SNSB-ZSM	Michael Franzen	Germany; 48.386, 11.82
GU682190	FBAQU200-09	BC ZSM AQU 00200	BOLD:AAI7225	623[0n]	22-Jun-2009	*Chalcolestes viridis*	larvae	SNSB-ZSM	Monika Hess	Germany; 49.032, 10.369
MT298319	ZPLOD179-20	MIB:ZPL:07979	BOLD:AAI7225	658[0n]		*Chalcolestes viridis*	adult	Milano Bicocca	Giacomo Assandri	Italy; 37.9, 14.9
MT298313	ZPLOD164-20	MIB:ZPL:07964	BOLD:AAI7225	658[0n]		*Chalcolestes viridis*	adult	Milano Bicocca	Giacomo Assandri	Italy; 39.4, 8.7
MT298314	ZPLOD165-20	MIB:ZPL:07965	BOLD:AAI7225	658[0n]		*Chalcolestes viridis*	adult	Giacomo Assandri	Giacomo Assandri	Italy; 40.3, 9.5
MT298315	ZPLOD167-20	MIB:ZPL:07967	BOLD:AAI7225	658[0n]		*Chalcolestes viridis*	adult	Milano Bicocca	Giacomo Assandri	Italy; 40.9, 16.4
MT298312	ZPLOD168-20	MIB:ZPL:07968	BOLD:ADR7794	658[0n]		*Chalcolestes viridis*	adult	Milano Bicocca	Giacomo Assandri	Italy; 40.9, 16.4
MT298317	ZPLOD174-20	MIB:ZPL:07974	BOLD:AAI7225	658[0n]		*Chalcolestes viridis*	adult	Milano Bicocca	Giacomo Assandri	Italy; 42.9, 12.3
MT298320	ZPLOD181-20	MIB:ZPL:07981	BOLD:AAI7225	658[0n]		*Chalcolestes viridis*	adult	Milano Bicocca	Giacomo Assandri	Italy; 45.7, 8.9
MT298318	ZPLOD177-20	MIB:ZPL:07977	BOLD:AAI7225	658[0n]		*Chalcolestes viridis*	adult	Milano Bicocca	Giacomo Assandri	Italy; 45.8, 13.1
MT298316	ZPLOD173-20	MIB:ZPL:07973	BOLD:AAI7225	658[0n]		*Chalcolestes viridis*	adult	Milano Bicocca	Giacomo Assandri	Italy; 45.9, 11.1
MW490529	TRDOD016-14	TRD-ODO34	BOLD:ACP7016	658[0n]	05-Jun-2014	*Coenagrion armatum*	adult	NTNU	Jon K. Skei	Norway; 63.217, 10.307
MW490383	TRDOD017-14	TRD-ODO35	BOLD:ACP7016	658[0n]	05-Jun-2014	*Coenagrion armatum*	adult	NTNU	Jon K. Skei	Norway; 63.217, 10.307
MW490202	TRDOD018-14	TRD-ODO36	BOLD:ACP7016	658[0n]	05-Jun-2014	*Coenagrion armatum*	adult	NTNU	Jon K. Skei	Norway; 63.217, 10.307
MW490147	TRDOD019-14	TRD-ODO37	BOLD:ACP7016	658[0n]	05-Jun-2014	*Coenagrion armatum*	adult	NTNU	Jon K. Skei	Norway; 63.217, 10.307
MT298328	ZPLOD189-20	MIB:ZPL:07989	BOLD:ADK6267	658[0n]		*Coenagrion caerulescens*	adult	Milano Bicocca	Giacomo Assandri	Italy; 37.5, 13.2
MT298329	ZPLOD190-20	MIB:ZPL:07990	BOLD:ADK6267	658[0n]		*Coenagrion caerulescens*	adult	Milano Bicocca	Giacomo Assandri	Italy; 40.4, 8.6
MT298325	ZPLOD192-20	MIB:ZPL:07992	BOLD:ADK6267	658[0n]		*Coenagrion caerulescens*	adult	Milano Bicocca	Giacomo Assandri	Italy; 40.9, 16.6
MT298324	ZPLOD198-20	MIB:ZPL:07998	BOLD:ADK6267	658[0n]		*Coenagrion caerulescens*	adult	Milano Bicocca	Giacomo Assandri	Italy; 41.6, 14.8
MT298326	ZPLOD194-20	MIB:ZPL:07994	BOLD:ADK6267	658[0n]		*Coenagrion caerulescens*	adult	Milano Bicocca	Giacomo Assandri	Italy; 44.5, 7.7
MT298327	ZPLOD195-20	MIB:ZPL:07995	BOLD:ADK6267	658[0n]		*Coenagrion caerulescens*	adult	Milano Bicocca	Giacomo Assandri	Italy; 44.5, 7.7
MT298322	ZPLOD196-20	MIB:ZPL:07996	BOLD:ADK6267	658[0n]		*Coenagrion caerulescens*	adult	Milano Bicocca	Giacomo Assandri	Italy; 44.5, 7.7
MT298323	ZPLOD197-20	MIB:ZPL:07997	BOLD:ADK6267	658[0n]		*Coenagrion caerulescens*	adult	Milano Bicocca	Giacomo Assandri	Italy; 44.5, 7.7
MW490311	FBAQU556-10	BC ZSM AQU 00461	BOLD:ACH0316	658[0n]	27-Apr-2004	*Coenagrion hastulatum*	larvae	SNSB-ZSM	Monika Hess	Germany; 47.816, 11.899
MW490322	TRDOD042-14	TRD-ODO60	BOLD:ACH0316	658[0n]	08-Jun-2014	*Coenagrion hastulatum*	adult	NTNU	Jon K. Skei	Norway; 63.306, 10.813
MW490200	TRDOD075-14	TRD-ODO93	BOLD:ACH0316	630[0n]	01-Jul-2014	*Coenagrion hastulatum*	adult	NTNU	Jon K. Skei	Norway; 63.374, 10.286
MW490294	TRDOD043-14	TRD-ODO61	BOLD:ACH0316	658[0n]	14-Jun-2014	*Coenagrion hastulatum*	adult	NTNU	Jon K. Skei	Norway; 63.405, 10.729
MW490151	TRDOD076-14	TRD-ODO94	BOLD:ACH0316	630[1n]	19-Jul-2014	*Coenagrion hastulatum*	adult	NTNU	Jon K. Skei	Norway; 63.526, 11.051
MW490422	ODTRI004-14	TRD-ODO4	BOLD:ACH0316	631[0n]	29-Jul-2010	*Coenagrion hastulatum*	adult	NTNU	Jon K. Skei	Norway; 63.548, 10.996
MW490362	ODTRI006-14	TRD-ODO6	BOLD:ACH0316	621[0n]	29-Jul-2010	*Coenagrion hastulatum*	adult	NTNU	Jon K. Skei	Norway; 63.548, 10.996
MW490491	ODOPL072-19	OdoPL72	BOLD:ACH0316	636[0n]	02-Nov-2018	*Coenagrion hastulatum*	larvae	Lodz	Grzegorz Tonczyk	Poland; 51.3338, 19.8961
MW490261	PLSW010-20	ODOPL_144	BOLD:ACH0316	618[1n]	12-Jun-2019	*Coenagrion hastulatum*	adult	Lodz	Grzegorz Tonczyk	Poland; 54.112, 23.043
MW490350	TRDOD005-14	TRD-ODO23	BOLD:ACM5448	658[0n]	06-Jun-2014	*Coenagrion johanssoni*	adult	NTNU	Jon K. Skei	Norway; 63.321, 10.638
MW490152	TRDOD021-14	TRD-ODO39	BOLD:ACM5448	658[0n]	09-Jul-2014	*Coenagrion johanssoni*	adult	NTNU	Jon K. Skei	Norway; 63.54, 10.989
MW490315	TRDOD022-14	TRD-ODO40	BOLD:ACM5448	658[0n]	09-Jul-2014	*Coenagrion johanssoni*	adult	NTNU	Jon K. Skei	Norway; 63.54, 10.989
MW490137	ODTRI007-14	TRD-ODO7	BOLD:ACM5448	621[0n]	22-Aug-2010	*Coenagrion johanssoni*	adult	NTNU	Jon K. Skei	Norway; 63.548, 10.996
MW490112	TRDOD074-14	TRD-ODO92	BOLD:ACM5448	658[0n]	05-Jul-2014	*Coenagrion johanssoni*	adult	NTNU	Jon K. Skei	Norway; 63.548, 10.996
MW490187	FBAQU1429-13	GBOL00273	BOLD:ACG0797	658[0n]	27-Jun-2011	*Coenagrion mercuriale*	adult	SNSB-ZSM	Stefan Koch	Germany; 48.119, 10.456
MW490164	GBMIX947-14	GBOL10049	BOLD:ACG0797	658[0n]	23-Jul-2014	*Coenagrion mercuriale*		SNSB-ZSM	Michael Franzen	Germany; 48.409, 11.795
MW490446	FBAQU559-10	BC ZSM AQU 00464	BOLD:AAJ0782	658[0n]	05-Sep-2003	*Coenagrion ornatum*	larvae	SNSB-ZSM	Monika Hess	Germany; 48.35, 11.736

GenBank #	Process ID	Sample ID	BIN	Seq. Length	Collection Date	Identification	Life Stage	Institution (short)	Identifier	country; GPS
GU682176	FBAQU310-09	BC ZSM AQU 00310	BOLD:AAJ0782	658[0n]	17-Jun-2009	*Coenagrion ornatum*	adult	SNSB-ZSM	M. Schoen	Germany; 48.376, 11.842
MW490467	FBAQU1446-13	GBOL00290	BOLD:AAJ0782	658[0n]	26-May-2011	*Coenagrion ornatum*	adult	SNSB-ZSM	Michael Franzen	Germany; 48.3795, 11.841
MW490159	FBAQU1447-13	GBOL00291	BOLD:AAJ0782	658[0n]	26-May-2011	*Coenagrion ornatum*	adult	SNSB-ZSM	Michael Franzen	Germany; 48.3795, 11.841
MW490306	FBAQU1448-13	GBOL00292	BOLD:AAJ0782	658[0n]	26-May-2011	*Coenagrion ornatum*	adult	SNSB-ZSM	Michael Franzen	Germany; 48.3795, 11.841
MW490363	GBODO079-18	GBOL 20202	BOLD:AAJ0782	658[5n]	12-Jul-2018	*Coenagrion puella*	adult	SNSB-ZSM	Stefan Koch	Germany; 47.6668, 11.0506
MW490454	GBEPT918-14	GBOL05488	BOLD:AAJ0782	658[0n]	01-Jun-2014	*Coenagrion puella*	adult	SNSB-ZSM	Michael Franzen	Germany; 47.709, 10.852
MW490524	GBODO037-18	GBOL 20160	BOLD:AAJ0782	658[1n]	27-Jun-2018	*Coenagrion puella*	adult	SNSB-ZSM	Stefan Koch	Germany; 47.7561, 11.3698
MW490553	GBODO151-18	GBOL 20274	BOLD:AAJ0782	658[0n]	21-May-2018	*Coenagrion puella*	adult	SNSB-ZSM	Stefan Koch	Germany; 47.7561, 11.3698
MW490165	GBODO153-18	GBOL 20276	BOLD:AAJ0782	658[0n]	21-May-2018	*Coenagrion puella*	adult	SNSB-ZSM	Stefan Koch	Germany; 47.7561, 11.3698
MW490289	GBODO156-18	GBOL 20279	BOLD:AAJ0782	658[0n]	21-May-2018	*Coenagrion puella*	adult	SNSB-ZSM	Stefan Koch	Germany; 47.7561, 11.3698
MW490233	GBODO186-18	GBOL 20309	BOLD:AAJ0782	658[0n]	21-May-2018	*Coenagrion puella*	adult	SNSB-ZSM	Stefan Koch	Germany; 47.7561, 11.3698
MW490562	GBODO179-18	GBOL 20302	BOLD:AAJ0782	658[0n]	27-Jun-2018	*Coenagrion puella*	adult	SNSB-ZSM	Stefan Koch	Germany; 47.8252, 11.3012
HM901887	FBAQU528-10	BC ZSM AQU 00433	BOLD:AAJ0782	616[0n]	16-Jul-2009	*Coenagrion puella*	adult	SNSB-ZSM	F. Weihrauch	Germany; 47.895, 12.167
MW490498	GBODO176-18	GBOL 20299	BOLD:AAJ0782	658[0n]	22-May-2018	*Coenagrion puella*	adult	SNSB-ZSM	Stefan Koch	Germany; 48.0616, 10.6428
GU682174	FBAQU311-09	BC ZSM AQU 00311	BOLD:AAJ0782	658[0n]	20-May-2009	*Coenagrion puella*	adult	SNSB-ZSM	M. Schoen	Germany; 48.298, 11.822
MW490279	GBODO068-18	GBOL 20191	BOLD:AAJ0782	658[1n]	04-Jul-2018	*Coenagrion puella*	adult	SNSB-ZSM	Stefan Koch	Germany; 48.3305, 11.8047
MW490206	GBODO069-18	GBOL 20192	BOLD:AAJ0782	655[0n]	04-Jul-2018	*Coenagrion puella*	adult	SNSB-ZSM	Stefan Koch	Germany; 48.3537, 11.775
MW490336	GBEPT939-14	GBOL05509	BOLD:AAJ0782	658[0n]	22-May-2014	*Coenagrion puella*	adult	SNSB-ZSM	Michael Franzen	Germany; 48.378, 11.841
MW490381	GODO028-18	ZFMK-TIS-2010636	BOLD:AAJ0782	658[0n]	27-Jun-2017	*Coenagrion puella*	adult	ZFMK	T. Sellmeier	Germany; 50.8, 7.585
MW490247	GODO029-18	ZFMK-TIS-2010637	BOLD:AAJ0782	658[0n]	27-Jun-2017	*Coenagrion puella*	adult	ZFMK	T. Sellmeier	Germany; 50.8, 7.585
MW490307	GODO031-18	ZFMK-TIS-2010639	BOLD:AAJ0782	658[0n]	11-Jul-2017	*Coenagrion puella*	adult	ZFMK	T. Sellmeier	Germany; 50.8, 7.585
MW490437	GODO035-18	ZFMK-TIS-2612910	BOLD:AAJ0782	658[0n]	27-Jun-2017	*Coenagrion puella*	adult	ZFMK	T. Sellmeier	Germany; 50.8, 7.585
MW490406	GODO036-18	ZFMK-TIS-2612911	BOLD:AAJ0782	658[0n]	11-Jul-2017	*Coenagrion puella*	adult	ZFMK	T. Sellmeier	Germany; 50.8, 7.585
MW490447	ODOPL069-19	OdoPL69	BOLD:AAJ0782	635[0n]	02-Nov-2018	*Coenagrion puella*	larvae	Lodz	Grzegorz Tonczyk	Poland; 51.3338, 19.8961
MW490347	ODOPL070-19	OdoPL70	BOLD:AAJ0782	635[0n]	02-Nov-2018	*Coenagrion puella*	larvae	Lodz	Grzegorz Tonczyk	Poland; 51.3338, 19.8961
MW490269	ODOPL071-19	OdoPL71	BOLD:AAJ0782	631[0n]	02-Nov-2018	*Coenagrion puella*	larvae	Lodz	Grzegorz Tonczyk	Poland; 51.3338, 19.8961
MW490219	ODOPL102-19	OdoPL102	BOLD:AAJ0782	635[0n]	04-Nov-2018	*Coenagrion puella*	larvae	Lodz	Grzegorz Tonczyk	Poland; 51.6201, 19.3112
MW490442	ODOPL103-19	OdoPL103	BOLD:AAJ0782	635[0n]	04-Nov-2018	*Coenagrion puella*	larvae	Lodz	Grzegorz Tonczyk	Poland; 51.6201, 19.3112
MW490226	ODOPL104-19	OdoPL104	BOLD:AAJ0782	641[0n]	04-Nov-2018	*Coenagrion puella*	larvae	Lodz	Grzegorz Tonczyk	Poland; 51.6201, 19.3112
MW490340	PLSW008-20	ODOPL_142	BOLD:AAJ0782	658[1n]	12-Jun-2019	*Coenagrion puella*	adult	Lodz	Grzegorz Tonczyk	Poland; 54.112, 23.043
MW490176	GBEPT910-14	GBOL05480	BOLD:AAJ0782	658[0n]	01-Jun-2014	*Coenagrion pulchellum*	adult	SNSB-ZSM	Michael Franzen	Germany; 47.682, 10.731
MW490484	GBEPT911-14	GBOL05481	BOLD:AAJ0782	658[0n]	01-Jun-2014	*Coenagrion pulchellum*	adult	SNSB-ZSM	Michael Franzen	Germany; 47.682, 10.731
MW490488	GBODO154-18	GBOL 20277	BOLD:AAJ0782	658[0n]	21-May-2018	*Coenagrion pulchellum*	adult	SNSB-ZSM	Stefan Koch	Germany; 47.7561, 11.3698
MW490493	GBODO155-18	GBOL 20278	BOLD:AAJ0782	658[0n]	21-May-2018	*Coenagrion pulchellum*	adult	SNSB-ZSM	Stefan Koch	Germany; 47.7561, 11.3698
MW490107	GBODO157-18	GBOL 20280	BOLD:AAJ0782	658[0n]	21-May-2018	*Coenagrion pulchellum*	adult	SNSB-ZSM	Stefan Koch	Germany; 47.7561, 11.3698
MW490440	GBODO170-18	GBOL 20293	BOLD:AAJ0782	658[0n]	27-Jun-2018	*Coenagrion pulchellum*	adult	SNSB-ZSM	Stefan Koch	Germany; 47.7561, 11.3698
MW490198	GBODO188-18	GBOL 20311	BOLD:AAJ0782	647[0n]	21-May-2018	*Coenagrion pulchellum*	adult	SNSB-ZSM	Stefan Koch	Germany; 47.7561, 11.3698
MW490436	GBODO189-18	GBOL 20312	BOLD:AAJ0782	533[1n]	21-May-2018	*Coenagrion pulchellum*	adult	SNSB-ZSM	Stefan Koch	Germany; 47.7561, 11.3698
MW490149	GBODO129-18	GBOL 20252	BOLD:AAJ0782	658[0n]	27-Jun-2018	*Coenagrion pulchellum*	adult	SNSB-ZSM	Stefan Koch	Germany; 47.7852, 11.3619
MW490139	GBODO137-18	GBOL 20260	BOLD:AAJ0782	658[0n]	27-Jun-2018	*Coenagrion pulchellum*	adult	SNSB-ZSM	Stefan Koch	Germany; 47.7852, 11.3619
MW490184	GBMIX558-14	GBOL09090	BOLD:AAJ0782	658[0n]	04-Jul-2014	*Coenagrion pulchellum*		SNSB-ZSM	Hans Muehle	Germany; 47.811, 11.59

GenBank #	Process ID	Sample ID	BIN	Seq. Length	Collection Date	Identification	Life Stage	Institution (short)	Identifier	country; GPS
HM901888	FBAQU529-10	BC ZSM AQU 00434	BOLD:AAJ0782	587[0n]	16-Jul-2009	*Coenagrion pulchellum*	adult	SNSB-ZSM	F. Weihrauch	Germany; 47.895, 12.167
HM901861	FBAQU488-10	BC ZSM AQU 00393	BOLD:AAJ0782	551[1n]	16-Jul-2009	*Coenagrion pulchellum*	adult	SNSB-ZSM	S. V. Ober	Germany; 47.908, 11.523
MW490120	GBEPT902-14	GBOL05472	BOLD:AAJ0782	658[0n]	13-Jun-2014	*Coenagrion pulchellum*	adult	SNSB-ZSM	Michael Franzen	Germany; 49.085, 11.623
MW490114	GBEPT903-14	GBOL05473	BOLD:AAJ0782	658[0n]	13-Jun-2014	*Coenagrion pulchellum*	adult	SNSB-ZSM	Michael Franzen	Germany; 49.085, 11.623
MW490540	GODO007-18	ZFMK-TIS-2556889	BOLD:AAJ0782	658[0n]		*Coenagrion pulchellum*		ZFMK	Laura von der Mark	Germany; 53.4255, 12.7791
MW490282	GODO008-18	ZFMK-TIS-2556891	BOLD:AAJ0782	658[0n]		*Coenagrion pulchellum*		ZFMK	Laura von der Mark	Germany; 53.4255, 12.7791
MW490183	GODO009-18	ZFMK-TIS-2556897	BOLD:AAJ0782	658[0n]		*Coenagrion pulchellum*		ZFMK	Laura von der Mark	Germany; 53.4255, 12.7791
MW490181	GODO010-18	ZFMK-TIS-2556899	BOLD:AAJ0782	658[0n]		*Coenagrion pulchellum*		ZFMK	Laura von der Mark	Germany; 53.4255, 12.7791
MW490126	ZMBN958-17	IBOL Kurs2017-JE1	BOLD:AAJ0782	658[0n]	23-Aug-2017	*Coenagrion pulchellum*	larvae	Bergen	Jonas M. Eliassen	Norway; 60.333, 5.359
MW490318	TRDOD077-14	TRD-ODO95	BOLD:AAJ0782	658[0n]	19-Jul-2014	*Coenagrion pulchellum*	adult	NTNU	Jon K. Skei	Norway; 63.526, 11.051
MW490522	ODTRI010-14	TRD-ODO10	BOLD:AAJ0782	658[0n]	29-Jul-2010	*Coenagrion pulchellum*	adult	NTNU	Jon K. Skei	Norway; 63.548, 10.996
MW490532	ODTRI008-14	TRD-ODO8	BOLD:AAJ0782	622[0n]	29-Jul-2010	*Coenagrion pulchellum*	adult	NTNU	Jon K. Skei	Norway; 63.548, 10.996
MW490154	ODTRI009-14	TRD-ODO9	BOLD:AAJ0782	658[0n]	29-Jul-2010	*Coenagrion pulchellum*	adult	NTNU	Jon K. Skei	Norway; 63.548, 10.996
MW490274	TRDOD044-14	TRD-ODO62	BOLD:AAJ0782	658[0n]	14-Jul-2014	*Coenagrion pulchellum*	adult	NTNU	Jon K. Skei	Norway; 63.653, 11.093
MW490240	PLSW017-20	ODOPL_151	BOLD:AAJ0782	658[0n]	12-Jun-2019	*Coenagrion pulchellum*	adult	Lodz	Grzegorz Tonczyk	Poland; 54.112, 23.043
MW490123	PLSW019-20	ODOPL_153	BOLD:AAJ0782	658[0n]	12-Jun-2019	*Coenagrion pulchellum*	adult	Lodz	Grzegorz Tonczyk	Poland; 54.112, 23.043
MW490438	EDF007-18	NOaS1-2019_Odo0186	BOLD:ACP4983	658[0n]	15-Jun-2018	*Coenagrion scitulum*	adult	NHMW	Iris Fischer	Austria; ,
MW490472	EDF008-18	NOaS1-2019_Odo0187	BOLD:ACP4983	658[0n]	15-Jun-2018	*Coenagrion scitulum*	adult	NHMW	Iris Fischer	Austria; ,
MW490158	GBEPT907-14	GBOL05477	BOLD:ACP4983	658[0n]	12-Jun-2014	*Coenagrion scitulum*	adult	SNSB-ZSM	Michael Franzen	Austria; 47.782, 16.848
MW490178	GBEPT888-14	GBOL05458	BOLD:ACP4983	658[0n]	06-Jun-2014	*Coenagrion scitulum*	adult	SNSB-ZSM	Hans Muehle	Greece; 41.114, 26.213
HM901889	FBAQU530-10	BC ZSM AQU 00435	BOLD:AAJ5749	601[0n]	16-Jul-2009	*Cordulegaster bidentata*	larvae	SNSB-ZSM	Monika Hess	Germany; 47.687, 12.056
MW490281	GBMIX942-14	GBOL10044	BOLD:AAJ5773	658[0n]	23-Jul-2014	*Cordulegaster boltonii*		SNSB-ZSM	Emmanuel FA Toussaint	France; 48.639, 0.085
HM901890	FBAQU531-10	BC ZSM AQU 00436	BOLD:AAJ5773	571[1n]	23-Aug-2009	*Cordulegaster boltonii*	larvae	SNSB-ZSM	Monika Hess	Germany; 47.821, 11.904
MW490410	GODO037-18	ZFMK-TIS-2007235	BOLD:AAJ5773	658[0n]	03-Apr-2017	*Cordulegaster boltonii*		ZFMK	S. Schueler	Germany; 50.9081, 7.14023
MW490296	ZMBN037-15	Kurs2015-AB1	BOLD:AAJ5773	658[0n]	19-Aug-2015	*Cordulegaster boltonii*	larvae	Bergen	Steffen Roth	Norway; 60.333, 5.359
MW490492	TRDOD083-14	TRD-ODO101	BOLD:AAJ5773	658[0n]	04-Jul-2008	*Cordulegaster boltonii*	adult	NTNU	Dag Dolmen	Norway; 63.5, 10.983
MW490228	TRDOD029-14	TRD-ODO47	BOLD:AAJ5773	658[0n]	19-Jul-2014	*Cordulegaster boltonii*	adult	NTNU	Jon K. Skei	Norway; 63.543, 11.033
MW490286	TRDOD068-14	TRD-ODO86	BOLD:AAJ5773	658[0n]	05-Aug-2014	*Cordulegaster boltonii*	larvae	NTNU	Gaute Kjaerstad	Norway; 64.076, 11.717
MW490469	TRDOD084-14	TRD-ODO102	BOLD:AAJ5773	658[0n]	31-Jul-2009	*Cordulegaster boltonii*	adult	NTNU	Dag Dolmen	Norway; 64.432, 11.001
MW490369	EDF003-18	NOaS1-2019_Odo0181	BOLD:ACQ4796	658[0n]	16-Jun-2018	*Cordulegaster heros*	adult	NHMW	Iris Fischer	Austria; ,
MW490316	EDF009-18	NOaS1-2019_Odo0200	BOLD:ACQ4796	658[0n]	14-Jul-2018	*Cordulegaster heros*	adult	NHMW	Iris Fischer	Austria; ,
MW490260	RODI004-20	BOCO-CH-1		499[6n]	17-Jul-2012	*Cordulegaster heros*	adult	Babes-Bolyai	Lujza Keresztes	Bulgaria; 41.846, 24.426
LT715983	GBMIN88645-17	LT715983	BOLD:ACQ4354	519[0n]		*Cordulegaster picta*		GenBank, NCBI	0	Russia; 44.6668, 37.9192
MT298395	ZPLOD314-20	MIB:ZPL:08114	BOLD:ACQ2278	658[0n]		*Cordulegaster trinacriae*	adult	Milano Bicocca	Giacomo Assandri	Italy; 40.8, 16.4
MT298394	ZPLOD315-20	MIB:ZPL:08115	BOLD:ACQ2278	658[0n]		*Cordulegaster trinacriae*	adult	Giacomo Assandri	Giacomo Assandri	Italy; 41.7, 14.9
MW490331	GBMIX687-14	GBOL09789	BOLD:AAJ5771	614[0n]	23-Jul-2014	*Cordulia aenea*		SNSB-ZSM	Emmanuel FA Toussaint	France; 48.639, 0.085
MW490103	GBODO092-18	GBOL 20215	BOLD:AAJ5771	315[1n]	12-Jul-2018	*Cordulia aenea*	adult	SNSB-ZSM	Stefan Koch	Germany; 47.6668, 11.0506
MW490452	GBEPT915-14	GBOL05485	BOLD:AAJ5771	642[0n]	01-Jun-2014	*Cordulia aenea*	adult	SNSB-ZSM	Michael Franzen	Germany; 47.682, 10.731
MW490133	GBODO146-18	GBOL 20269	BOLD:AAJ5771	652[2n]	22-Apr-2018	*Cordulia aenea*	adult	SNSB-ZSM	Stefan Koch	Germany; 47.7561, 11.3698
MW490455	GBODO132-18	GBOL 20255	BOLD:AAJ5771	318[3n]	21-May-2018	*Cordulia aenea*	adult	SNSB-ZSM	Stefan Koch	Germany; 47.7852, 11.3619
HM901862	FBAQU489-10	BC ZSM AQU 00394	BOLD:AAJ5771	658[0n]	26-May-2009	*Cordulia aenea*	adult	SNSB-ZSM	S. V. Ober	Germany; 48.048, 11.468

GenBank #	Process ID	Sample ID	BIN	Seq. Length	Collection Date	Identification	Life Stage	Institution (short)	Identifier	country; GPS
MW490526	GBODO144-18	GBOL 20267	BOLD:AAJ5771	315[0n]	12-May-2018	*Cordulia aenea*	adult	SNSB-ZSM	Stefan Koch	Germany; 48.0616, 10.6428
MW490252	TRDOD007-14	TRD-ODO25	BOLD:AAJ5771	658[0n]	01-Jul-2014	*Cordulia aenea*	adult	NTNU	Jon K. Skei	Norway; 63.382, 10.869
MW490271	TRDOD004-14	TRD-ODO22	BOLD:ACP6876	658[0n]	07-Jul-2014	*Cordulia aenea*	adult	NTNU	Jon K. Skei	Norway; 63.477, 11.252
MW490358	TRDOD006-14	TRD-ODO24	BOLD:AAJ5771	658[0n]	09-Jul-2014	*Cordulia aenea*	adult	NTNU	Jon K. Skei	Norway; 63.54, 10.989
MW490480	TRDOD008-14	TRD-ODO26	BOLD:ACP6876	658[0n]	05-Jul-2014	*Cordulia aenea*	adult	NTNU	Jon K. Skei	Norway; 63.548, 10.996
MW490390	ODOPL067-19	OdoPL67	BOLD:ACP6876	639[0n]	02-Nov-2018	*Cordulia aenea*	larvae	Lodz	Grzegorz Tonczyk	Poland; 51.3338, 19.8961
MW490424	ODOPL068-19	OdoPL68	BOLD:ACP6876	641[0n]	02-Nov-2018	*Cordulia aenea*	larvae	Lodz	Grzegorz Tonczyk	Poland; 51.3338, 19.8961
MW490248	PLSW024-20	ODOPL_158	BOLD:ACP6876	658[0n]	10-Jun-2019	*Cordulia aenea*	adult	Lodz	Grzegorz Tonczyk	Poland; 54.088, 23.018
MW490554	PLSW025-20	ODOPL_159	BOLD:ACP6876	654[0n]	10-Jun-2019	*Cordulia aenea*	adult	Lodz	Grzegorz Tonczyk	Poland; 54.088, 23.018
MW490222	PLSW026-20	ODOPL_160	BOLD:ACP6876	654[0n]	10-Jun-2019	*Cordulia aenea*	adult	Lodz	Grzegorz Tonczyk	Poland; 54.088, 23.018
MW490268	GBEPT947-14	GBOL05517	BOLD:AAI9726	633[0n]	13-Jun-2014	*Crocothemis erythraea*	adult	SNSB-ZSM	Michael Franzen	Austria; 47.931, 16.746
GU682175	FBAQU312-09	BC ZSM AQU 00312	BOLD:AAI9726	658[0n]	13-Jun-2009	*Crocothemis erythraea*	adult	SNSB-ZSM	M. Schoen	Germany; 48.298, 11.822
MW490533	GODO042-19	ZFMK-TIS-2616202	BOLD:AAI9726	658[1n]	05-Jun-2018	*Crocothemis erythraea*	adult	Potsdam	Udo Rothe	Germany; 52.4666, 12.6981
MW490565	PLSW069-20	ODOPL_203	BOLD:AAI9726	658[0n]	28-Jul-2019	*Crocothemis erythraea*	adult	Lodz	Grzegorz Tonczyk	Poland; 51.573, 19.06
MW490327	PLSW070-20	ODOPL_204	BOLD:AAI9726	658[0n]	28-Jul-2019	*Crocothemis erythraea*	adult	Lodz	Grzegorz Tonczyk	Poland; 51.573, 19.06
MW490321	PLSW071-20	ODOPL_205	BOLD:AAI9726	647[0n]	28-Jul-2019	*Crocothemis erythraea*	adult	Lodz	Grzegorz Tonczyk	Poland; 51.573, 19.06
MT298407	ZPLOD334-20	MIB:ZPL:08134	BOLD:ABU6643	658[0n]		*Diplacodes lefebvrii*	adult	Milano Bicocca	Giacomo Assandri	Italy; 39.2, 8.2
MT298406	ZPLOD335-20	MIB:ZPL:08135	BOLD:ABU6643	658[0n]		*Diplacodes lefebvrii*	adult	Milano Bicocca	Giacomo Assandri	Italy; 39.2, 8.2
GU682183	FBAQU015-09	BC ZSM AQU 00015	BOLD:AAA2218	644[0n]	11-Apr-2009	*Enallagma cyathigerum*	larvae	SNSB-ZSM	Monika Hess	Germany; ,
MW490118	GBODO100-18	GBOL 20223	BOLD:AAA2218	658[0n]	12-Jul-2018	*Enallagma cyathigerum*	adult	SNSB-ZSM	Stefan Koch	Germany; 47.6656, 11.0246
MW490250	GBODO040-18	GBOL 20163	BOLD:AAA2218	658[2n]	05-Sep-2018	*Enallagma cyathigerum*	adult	SNSB-ZSM	Stefan Koch	Germany; 47.6668, 11.0506
MW490528	GBODO050-18	GBOL 20173	BOLD:AAA2218	567[2n]	05-Sep-2018	*Enallagma cyathigerum*	adult	SNSB-ZSM	Stefan Koch	Germany; 47.6668, 11.0506
MW490557	GBODO051-18	GBOL 20174	BOLD:AAA2218	511[3n]	05-Sep-2018	*Enallagma cyathigerum*	adult	SNSB-ZSM	Stefan Koch	Germany; 47.6668, 11.0506
MW490117	GBODO052-18	GBOL 20175	BOLD:AAA2218	658[2n]	05-Sep-2018	*Enallagma cyathigerum*	adult	SNSB-ZSM	Stefan Koch	Germany; 47.6668, 11.0506
MW490343	GBODO054-18	GBOL 20177	BOLD:AAA2218	658[3n]	05-Sep-2018	*Enallagma cyathigerum*	adult	SNSB-ZSM	Stefan Koch	Germany; 47.6668, 11.0506
MW490359	GBODO072-18	GBOL 20195	BOLD:AAA2218	577[0n]	12-Jul-2018	*Enallagma cyathigerum*	adult	SNSB-ZSM	Stefan Koch	Germany; 47.6668, 11.0506
MW490535	GBODO076-18	GBOL 20199	BOLD:AAA2218	658[1n]	12-Jul-2018	*Enallagma cyathigerum*	adult	SNSB-ZSM	Stefan Koch	Germany; 47.6668, 11.0506
MW490512	GBODO096-18	GBOL 20219	BOLD:AAA2218	658[2n]	12-Jul-2018	*Enallagma cyathigerum*	adult	SNSB-ZSM	Stefan Koch	Germany; 47.6668, 11.0506
MW490301	GBEPT912-14	GBOL05482	BOLD:AAA2218	658[0n]	01-Jun-2014	*Enallagma cyathigerum*	adult	SNSB-ZSM	Michael Franzen	Germany; 47.682, 10.731
MW490385	GBODO024-18	GBOL 20147	BOLD:AAA2218	658[1n]	27-Jul-2018	*Enallagma cyathigerum*	adult	SNSB-ZSM	Stefan Koch	Germany; 47.8252, 11.3012
MW490190	GBODO172-18	GBOL 20295	BOLD:AAA2218	658[0n]	22-May-2018	*Enallagma cyathigerum*	adult	SNSB-ZSM	Stefan Koch	Germany; 48.0616, 10.6428
MW490537	GBODO181-18	GBOL 20304	BOLD:AAA2218	658[0n]	21-Jun-2018	*Enallagma cyathigerum*	adult	SNSB-ZSM	Stefan Koch	Germany; 48.0616, 10.6428
HQ563103	FBAQU490-10	BC ZSM AQU 00395	BOLD:AAA2218	616[0n]	21-May-2009	*Enallagma cyathigerum*	adult	SNSB-ZSM	S. V. Ober	Germany; 48.107, 11.458
GU682173	FBAQU313-09	BC ZSM AQU 00313	BOLD:AAA2218	658[0n]	20-May-2009	*Enallagma cyathigerum*	adult	SNSB-ZSM	M. Schoen	Germany; 48.298, 11.822
HQ563105	FBAQU532-10	BC ZSM AQU 00437	BOLD:AAA2218	658[0n]	31-Jul-2009	*Enallagma cyathigerum*	adult	SNSB-ZSM	F. Weihrauch	Germany; 48.72, 11.676
MW490408	FBAQU1504-13	GBOL00348	BOLD:AAA2218	658[0n]	22-May-2012	*Enallagma cyathigerum*	adult	SNSB-ZSM	Reinhard Mueller	Germany; 52.2443, 13.1185
MW490300	GODO011-18	ZFMK-TIS-2556883	BOLD:AAA2218	658[0n]		*Enallagma cyathigerum*		ZFMK	Laura von der Mark	Germany; 53.4255, 12.7791
MW490460	GODO012-18	ZFMK-TIS-2556885	BOLD:AAA2218	658[0n]		*Enallagma cyathigerum*		ZFMK	Laura von der Mark	Germany; 53.4255, 12.7791
MW490166	GODO013-18	ZFMK-TIS-2556893	BOLD:AAA2218	658[0n]		*Enallagma cyathigerum*		ZFMK	Laura von der Mark	Germany; 53.4255, 12.7791
MW490354	GODO014-18	ZFMK-TIS-2556895	BOLD:AAA2218	658[0n]		*Enallagma cyathigerum*		ZFMK	Laura von der Mark	Germany; 53.4255, 12.7791
MW490548	ZMBN960-17	IBOL Kurs2017-JE3	BOLD:AAA2218	658[0n]	22-Aug-2017	*Enallagma cyathigerum*	larvae	Bergen	Jonas M. Eliassen	Norway; 60.579, 5.272

GenBank #	Process ID	Sample ID	BIN	Seq. Length	Collection Date	Identification	Life Stage	Institution (short)	Identifier	country; GPS
MW490227	TRDOD072-14	TRD-ODO90	BOLD:AAA2218	658[0n]	01-Jul-2014	*Enallagma cyathigerum*	adult	NTNU	Jon K. Skei	Norway; 63.375, 10.292
MW490225	TRDOD024-14	TRD-ODO42	BOLD:AAA2218	658[0n]	09-Jul-2014	*Enallagma cyathigerum*	adult	NTNU	Jon K. Skei	Norway; 63.54, 10.989
MW490218	ODTRI011-14	TRD-ODO11	BOLD:AAA2218	602[0n]	29-Jul-2010	*Enallagma cyathigerum*	adult	NTNU	Jon K. Skei	Norway; 63.548, 10.996
MW490136	ODTRI012-14	TRD-ODO12	BOLD:AAA2218	611[0n]	29-Jul-2010	*Enallagma cyathigerum*	adult	NTNU	Jon K. Skei	Norway; 63.548, 10.996
MW490411	TRDOD073-14	TRD-ODO91	BOLD:AAA2218	658[0n]	14-Jul-2014	*Enallagma cyathigerum*	adult	NTNU	Jon K. Skei	Norway; 63.653, 11.093
MW490507	HETFI049-11	FinHet49	BOLD:AAA2218	658[0n]	31-Jul-2010	*Enallagma cyathigerum*	adult	Bergen	Steffen Roth	Norway; 69.231, 29.1609
MW490193	HETFI052-11	FinHet52	BOLD:AAA2218	658[0n]	27-Jul-2010	*Enallagma cyathigerum*	adult	Bergen	Steffen Roth	Norway; 69.8237, 25.2009
MW490550	PLSW072-20	ODOPL_206	BOLD:AAA2218	658[0n]	28-Jul-2019	*Enallagma cyathigerum*	adult	Lodz	Grzegorz Tonczyk	Poland; 51.573, 19.06
MW490276	PLSW073-20	ODOPL_207	BOLD:AAA2218	633[0n]	28-Jul-2019	*Enallagma cyathigerum*	adult	Lodz	Grzegorz Tonczyk	Poland; 51.573, 19.06
MW490142	ODOPL126-19	OdoPL126	BOLD:AAA2218	640[0n]	17-Nov-2018	*Enallagma cyathigerum*	larvae	Lodz	Grzegorz Tonczyk	Poland; 51.5799, 19.2154
MW490167	ODOPL100-19	OdoPL100	BOLD:AAA2218	636[0n]	04-Nov-2018	*Enallagma cyathigerum*	larvae	Lodz	Grzegorz Tonczyk	Poland; 51.6201, 19.3112
MW490513	ODOPL101-19	OdoPL101	BOLD:AAA2218	634[0n]	04-Nov-2018	*Enallagma cyathigerum*	larvae	Lodz	Grzegorz Tonczyk	Poland; 51.6201, 19.3112
MW490503	PLSW016-20	ODOPL_150	BOLD:AAA2218	658[0n]	12-Jun-2019	*Enallagma cyathigerum*	adult	Lodz	Grzegorz Tonczyk	Poland; 54.112, 23.043
MW490544	FBAQU1443-13	GBOL00287	BOLD:ACG0805	658[0n]	20-May-2012	*Epitheca bimaculata*	adult	SNSB-ZSM	Stefan Koch	Germany; 47.6328, 10.5381
HM901863	FBAQU491-10	BC ZSM AQU 00396	BOLD:AAL4439	627[0n]	18-Jun-2009	*Erythromma lindenii*	adult	SNSB-ZSM	S. V. Ober	Germany; 48.237, 11.506
MW490287	FBAQU562-10	BC ZSM AQU 00467	BOLD:AAL4439	658[0n]	29-Jun-2005	*Erythromma lindenii*	larvae	SNSB-ZSM	Monika Hess	Germany; 48.878, 11.159
MW490173	GBODO087-18	GBOL 20210	BOLD:AAA4234	318[2n]	12-Jul-2018	*Erythromma najas*	adult	SNSB-ZSM	Stefan Koch	Germany; 47.6668, 11.0506
HM901864	FBAQU492-10	BC ZSM AQU 00397	BOLD:AAA4234	600[0n]	25-May-2009	*Erythromma najas*	adult	SNSB-ZSM	S. V. Ober	Germany; 48.107, 11.458
MW490458	FBAQU563-10	BC ZSM AQU 00468	BOLD:AAA4234	658[0n]	18-Aug-1993	*Erythromma najas*	larvae	SNSB-ZSM	Monika Hess	Germany; 48.775, 13.005
MW490518	TRDOD020-14	TRD-ODO38	BOLD:AAA4234	658[0n]	05-Jun-2014	*Erythromma najas*	adult	NTNU	Jon K. Skei	Norway; 63.217, 10.307
MW490330	TRDOD065-14	TRD-ODO83	BOLD:AAA4234	658[0n]	09-Jul-2008	*Erythromma najas*	adult	NTNU	Jon K. Skei	Norway; 63.217, 10.307
MW490116	TRDOD066-14	TRD-ODO84	BOLD:AAA4234	658[0n]	09-Jul-2008	*Erythromma najas*	adult	NTNU	Jon K. Skei	Norway; 63.217, 10.307
MW490547	TRDOD060-14	TRD-ODO78	BOLD:AAA4234	658[0n]	24-Jul-2009	*Erythromma najas*	adult	NTNU	Jon K. Skei	Norway; 63.419, 10.655
MW490254	PLSW103-20	ODOPL_223	BOLD:AAA4234	658[0n]	07-May-2019	*Erythromma najas*	larvae	Lodz	Grzegorz Tonczyk	Poland; 51.551, 19.338
MW490156	ODOPL044-19	OdoPL44	BOLD:AAA4234	629[0n]	10-Oct-2018	*Erythromma najas*	larvae	Lodz	Grzegorz Tonczyk	Poland; 51.6184, 16.333
MW490342	ODOPL045-19	OdoPL45	BOLD:AAA4234	627[0n]	10-Oct-2018	*Erythromma najas*	larvae	Lodz	Grzegorz Tonczyk	Poland; 51.6184, 16.333
MW490430	ODOPL046-19	OdoPL46	BOLD:AAA4234	631[0n]	10-Oct-2018	*Erythromma najas*	larvae	Lodz	Grzegorz Tonczyk	Poland; 51.6184, 16.333
MW490485	ODOPL030-19	OdoPL30	BOLD:AAA4234	631[0n]	10-Oct-2018	*Erythromma najas*	larvae	Lodz	Grzegorz Tonczyk	Poland; 52.0552, 15.4302
MW490275	ODOPL031-19	OdoPL31	BOLD:AAA4234	627[0n]	10-Oct-2018	*Erythromma najas*	larvae	Lodz	Grzegorz Tonczyk	Poland; 52.0552, 15.4302
MW490262	ODOPL010-18	OdoPL10	BOLD:AAA4234	622[0n]	05-Oct-2018	*Erythromma najas*	larvae	Lodz	Grzegorz Tonczyk	Poland; 52.3855, 20.1954
MW490304	GBUPS192-14	GBOL02842	BOLD:AAL4437	662[2n]		*Erythromma viridulum*	adult	SNSB-ZSM	Lars Hendrich	Germany; ,
HM422048	FBAQU314-09	BC ZSM AQU 00314	BOLD:AAL4437	658[0n]	16-Jul-2009	*Erythromma viridulum*	adult	SNSB-ZSM	M. Schoen	Germany; 48.301, 11.814
MW490344	GBMIX949-14	GBOL10051	BOLD:AAL4437	630[0n]	23-Jul-2014	*Erythromma viridulum*		SNSB-ZSM	Michael Franzen	Germany; 48.386, 11.82
MW490243	GBMIX950-14	GBOL10052	BOLD:AAL4437	658[0n]	23-Jul-2014	*Erythromma viridulum*		SNSB-ZSM	Michael Franzen	Germany; 48.386, 11.82
MW490536	FBAQU565-10	BC ZSM AQU 00470	BOLD:AAL4437	658[0n]	21-May-1993	*Erythromma viridulum*	larvae	SNSB-ZSM	Monika Hess	Germany; 48.783, 13.015
MW490194	PLSW074-20	ODOPL_208	BOLD:AAL4437	658[0n]	19-Jul-2019	*Erythromma viridulum*	adult	Lodz	Grzegorz Tonczyk	Poland; 52.195, 18.58
MW490400	PLSW075-20	ODOPL_209	BOLD:AAL4437	658[0n]	19-Jul-2019	*Erythromma viridulum*	adult	Lodz	Grzegorz Tonczyk	Poland; 52.195, 18.58
MW490270	PLSW076-20	ODOPL_210	BOLD:AAL4437	637[0n]	19-Jul-2019	*Erythromma viridulum*	adult	Lodz	Grzegorz Tonczyk	Poland; 52.195, 18.58
#NV	ODBOL-1299	GBMIN88698-17	BOLD:ADC4839	657[0n]		*Gomphus pulchellus*		GenBank, NCBI		France
MT298460	ZPLOD825-20	MIB:ZPL:08625	BOLD:AAN0925	658[0n]		*Gomphus schneiderii*	Adult	Giacomo Assandri	Giacomo Assandri	Montenegro; 42.2, 19.1
MT298459	ZPLOD824-20	MIB:ZPL:08624	BOLD:AAN0925	658[0n]		*Gomphus schneiderii*	Adult	Giacomo Assandri	Giacomo Assandri	Montenegro; 42.3, 19.1

GenBank #	Process ID	Sample ID	BIN	Seq. Length	Collection Date	Identification	Life Stage	Institution (short)	Identifier	country; GPS
KX890970	GBMIN88703-17	KX890970	BOLD:ADK7683	657[0n]		*Gomphus simillimus*		GenBank, NCBI	0	other; ,
MW490388	FBAQU1445-13	GBOL00289	BOLD:AAN0925	566[0n]	26-May-2011	*Gomphus vulgatissimus*	adult	SNSB-ZSM	Michael Franzen	Germany; 48.36, 11.88
HQ563106	FBAQU534-10	BC ZSM AQU 00439	BOLD:AAN0925	658[0n]	31-Aug-2009	*Gomphus vulgatissimus*	larvae	SNSB-ZSM	Monika Hess	Germany; 49.038, 12.187
MW490189	GBMIX410-14	GBOL09702	BOLD:AAN0925	647[0n]	31-Jul-2013	*Gomphus vulgatissimus*	larvae	SNSB-ZSM	Christoph D. Schubart	Germany; 49.629, 11.804
MW490389	GBMIX414-14	GBOL09706	BOLD:AAN0925	658[0n]	31-Jul-2013	*Gomphus vulgatissimus*	larvae	SNSB-ZSM	Christoph D. Schubart	Germany; 49.63, 11.795
MW490208	GODO021-18	ZFMK-TIS-2010620	BOLD:AAN0925	658[0n]	30-May-2017	*Gomphus vulgatissimus*	adult	ZFMK	T. Sellmeier	Germany; 50.777, 7.365
MW490380	GBMIX668-14	GBOL09770	BOLD:AAN0925	658[0n]	13-Aug-2014	*Gomphus vulgatissimus*		SNSB-ZSM	Lars Hendrich	Germany; 51.364, 14.446
MW490530	GBEPT1040-14	GBOL06275	BOLD:AAN0925	658[0n]	05-Jun-2014	*Gomphus vulgatissimus*	larvae	SNSB-ZSM	Lars Hendrich	Germany; 51.503, 14.362
MW490224	GBEPT1041-14	GBOL06276	BOLD:AAN0925	640[0n]	05-Jun-2014	*Gomphus vulgatissimus*	larvae	SNSB-ZSM	Lars Hendrich	Germany; 51.503, 14.362
MW490545	GBEPT1731-14	GBOL09313	BOLD:AAN0925	657[0n]	13-Aug-2014	*Gomphus vulgatissimus*	larvae	SNSB-ZSM	Christoph D. Schubart	Germany; 51.589, 14.733
MW490360	ODOPL073-19	OdoPL73	BOLD:AAN0925	638[0n]	02-Nov-2018	*Gomphus vulgatissimus*	larvae	Lodz	Grzegorz Tonczyk	Poland; 51.3164, 19.9037
MW490453	PLSW044-20	ODOPL_178	BOLD:AAN0925	658[0n]	13-Jun-2019	*Gomphus vulgatissimus*	adult	Lodz	Grzegorz Tonczyk	Poland; 54.037, 23.203
MW490102	PLSW045-20	ODOPL_179	BOLD:AAN0925	658[0n]	13-Jun-2019	*Gomphus vulgatissimus*	adult	Lodz	Grzegorz Tonczyk	Poland; 54.037, 23.203
MW490241	PLSW046-20	ODOPL_180	BOLD:AAN0925	658[0n]	13-Jun-2019	*Gomphus vulgatissimus*	adult	Lodz	Grzegorz Tonczyk	Poland; 54.037, 23.203
MW490310	GBEPT943-14	GBOL05513	BOLD:AAE5570	658[0n]	12-Jun-2014	*Ischnura elegans*	adult	SNSB-ZSM	Michael Franzen	Austria; 47.782, 16.848
HM376192	FBAQU172-09	BC ZSM AQU 00172	BOLD:AAE5570	654[0n]	22-Jun-2009	*Ischnura elegans*	adult	SNSB-ZSM	Monika Hess	Germany; ,
MW490382	GBUPS191-14	GBOL02841	BOLD:AAE5570	684[0n]		*Ischnura elegans*	adult	SNSB-ZSM	Lars Hendrich	Germany; ,
MW490501	GBUPS193-14	GBOL02843	BOLD:AAE5570	683[1n]		*Ischnura elegans*	adult	SNSB-ZSM	Lars Hendrich	Germany; ,
MW490414	GBUPS194-14	GBOL02844	BOLD:AAE5570	657[1n]		*Ischnura elegans*	adult	SNSB-ZSM	Lars Hendrich	Germany; ,
MW490113	GBUPS195-14	GBOL02845	BOLD:AAE5570	668[0n]		*Ischnura elegans*	adult	SNSB-ZSM	Lars Hendrich	Germany; ,
MW490214	GBUPS197-14	GBOL02847	BOLD:AAE5570	669[0n]		*Ischnura elegans*	adult	SNSB-ZSM	Lars Hendrich	Germany; ,
MW490402	GBUPS201-14	GBOL02851	BOLD:AAE5570	646[0n]		*Ischnura elegans*	adult	SNSB-ZSM	Lars Hendrich	Germany; ,
MW490337	GBUPS202-14	GBOL02852	BOLD:AAE5570	674[0n]		*Ischnura elegans*	adult	SNSB-ZSM	Lars Hendrich	Germany; ,
MW490456	GBUPS205-14	GBOL02855	BOLD:AAE5570	574[0n]		*Ischnura elegans*	adult	SNSB-ZSM	Lars Hendrich	Germany; ,
MW490138	GBUPS206-14	GBOL02856	BOLD:AAE5570	615[0n]		*Ischnura elegans*	adult	SNSB-ZSM	Lars Hendrich	Germany; ,
MW490459	GBODO084-18	GBOL 20207	BOLD:AAE5570	658[0n]	12-Jul-2018	*Ischnura elegans*	adult	SNSB-ZSM	Stefan Koch	Germany; 47.6668, 11.0506
MW490420	GBEPT940-14	GBOL05510	BOLD:AAE5570	658[0n]	01-Jun-2014	*Ischnura elegans*	adult	SNSB-ZSM	Michael Franzen	Germany; 47.709, 10.852
MW490434	GBODO131-18	GBOL 20254	BOLD:AAE5570	658[1n]	17-Jun-2018	*Ischnura elegans*	adult	SNSB-ZSM	Stefan Koch	Germany; 47.7852, 11.3619
MW490413	GBODO021-18	GBOL 20144	BOLD:AAE5570	658[1n]	27-Jul-2018	*Ischnura elegans*	adult	SNSB-ZSM	Stefan Koch	Germany; 47.8252, 11.3012
HM901892	FBAQU536-10	BC ZSM AQU 00441	BOLD:AAE5570	588[0n]	16-Jul-2009	*Ischnura elegans*	adult	SNSB-ZSM	F. Weihrauch	Germany; 47.895, 12.167
HQ563104	FBAQU494-10	BC ZSM AQU 00399	BOLD:AAE5570	658[0n]	21-May-2009	*Ischnura elegans*	adult	SNSB-ZSM	S. V. Ober	Germany; 48.107, 11.458
HM901865	FBAQU495-10	BC ZSM AQU 00400	BOLD:AAE5570	601[0n]	18-Jun-2009	*Ischnura elegans*	adult	SNSB-ZSM	S. V. Ober	Germany; 48.237, 11.505
MW490215	GBODO061-18	GBOL 20184	BOLD:AAE5570	658[1n]	04-Jul-2018	*Ischnura elegans*	adult	SNSB-ZSM	Stefan Koch	Germany; 48.3305, 11.8047
MW490567	GBODO063-18	GBOL 20186	BOLD:AAE5570	658[2n]	04-Jul-2018	*Ischnura elegans*	adult	SNSB-ZSM	Stefan Koch	Germany; 48.3537, 11.775
MW490412	GBEPT938-14	GBOL05508	BOLD:AAE5570	658[0n]	22-May-2014	*Ischnura elegans*	adult	SNSB-ZSM	Michael Franzen	Germany; 48.378, 11.841
HM901891	FBAQU535-10	BC ZSM AQU 00440	BOLD:AAE5570	614[0n]	31-Jul-2009	*Ischnura elegans*	adult	SNSB-ZSM	F. Weihrauch	Germany; 48.72, 11.676
MW490486	FBAQU1611-13	BCZSM_ODO_0091	BOLD:AAE5570	407[0n]	05-May-2011	*Ischnura elegans*		SNSB-ZSM	Lars Hendrich	Germany; 52.12, 13.23
MW490302	GODO016-18	ZFMK-TIS-2556887	BOLD:AAE5570	658[0n]		*Ischnura elegans*		ZFMK	Bjoern Rulik	Germany; 53.4255, 12.7791
MW490135	GBEPT890-14	GBOL05460	BOLD:AAE5570	658[0n]	07-Jun-2014	*Ischnura elegans*	adult	SNSB-ZSM	Hans Muehle	Greece; 40.826, 25.99
MW490468	GBEPT891-14	GBOL05461	BOLD:AAE5570	658[0n]	10-Jun-2014	*Ischnura elegans*	adult	SNSB-ZSM	Hans Muehle	Greece; 40.846, 25.855
MW490169	TRDOD027-14	TRD-ODO45	BOLD:AAE5570	658[0n]	14-Jul-2014	*Ischnura elegans*	adult	NTNU	Jon K. Skei	Norway; 63.653, 11.093

GenBank #	Process ID	Sample ID	BIN	Seq. Length	Collection Date	Identification	Life Stage	Institution (short)	Identifier	country; GPS
MW490415	TRDOD090-14	TRD-ODO108	BOLD:AAE5570	658[0n]	30-Jul-2009	*Ischnura elegans*	adult	NTNU	Dag Dolmen	Norway; 63.679, 11.05
MW490515	TRDOD088-14	TRD-ODO106	BOLD:AAE5570	658[0n]	03-Jul-2008	*Ischnura elegans*	adult	NTNU	Dag Dolmen	Norway; 63.908, 11.588
MW490445	TRDOD089-14	TRD-ODO107	BOLD:AAE5570	658[0n]	31-Jul-2009	*Ischnura elegans*	adult	NTNU	Dag Dolmen	Norway; 64.572, 10.95
MT216297	ODOPL059-19	OdoPL59	BOLD:AAE5570	633[0n]	11-Oct-2018	*Ischnura elegans*	larvae	Lodz	Grzegorz Tonczyk	Poland; 50.4761, 17.9714
MT216291	ODOPL060-19	OdoPL60	BOLD:AAE5570	633[0n]	11-Oct-2018	*Ischnura elegans*	larvae	Lodz	Grzegorz Tonczyk	Poland; 50.4761, 17.9714
MT216299	ODOPL087-19	OdoPL87	BOLD:AAE5570	633[0n]	02-Nov-2018	*Ischnura elegans*	larvae	Lodz	Grzegorz Tonczyk	Poland; 51.3164, 19.9037
MT216286	ODOPL088-19	OdoPL88	BOLD:AAE5570	636[0n]	02-Nov-2018	*Ischnura elegans*	larvae	Lodz	Grzegorz Tonczyk	Poland; 51.3164, 19.9037
MT216295	ODOPL089-19	OdoPL89	BOLD:AAE5570	636[0n]	02-Nov-2018	*Ischnura elegans*	larvae	Lodz	Grzegorz Tonczyk	Poland; 51.3164, 19.9037
MT216293	ODOPL061-19	OdoPL61	BOLD:AAE5570	630[0n]	02-Nov-2018	*Ischnura elegans*	larvae	Lodz	Grzegorz Tonczyk	Poland; 51.3338, 19.8961
MT216292	ODOPL048-19	OdoPL48	BOLD:AAE5570	633[0n]	10-Oct-2018	*Ischnura elegans*	larvae	Lodz	Grzegorz Tonczyk	Poland; 51.6184, 16.333
MT216300	ODOPL049-19	OdoPL49	BOLD:AAE5570	629[0n]	10-Oct-2018	*Ischnura elegans*	larvae	Lodz	Grzegorz Tonczyk	Poland; 51.6184, 16.333
MW490409	ODOPL004-18	OdoPL4	BOLD:AAE5570	558[0n]	10-Oct-2018	*Ischnura elegans*	larvae	Lodz	Grzegorz Tonczyk	Poland; 51.7771, 19.4833
MT216289	ODOPL026-19	OdoPL26	BOLD:AAE5570	638[0n]	10-Oct-2018	*Ischnura elegans*	larvae	Lodz	Grzegorz Tonczyk	Poland; 52.0552, 15.4302
MT216294	ODOPL027-19	OdoPL27	BOLD:AAE5570	630[0n]	10-Oct-2018	*Ischnura elegans*	larvae	Lodz	Grzegorz Tonczyk	Poland; 52.0552, 15.4302
MT216287	ODOPL028-19	OdoPL28	BOLD:AAE5570	641[0n]	10-Oct-2018	*Ischnura elegans*	larvae	Lodz	Grzegorz Tonczyk	Poland; 52.0552, 15.4302
MT216298	ODOPL013-18	OdoPL13	BOLD:AAE5570	626[0n]	05-Oct-2018	*Ischnura elegans*	larvae	Lodz	Grzegorz Tonczyk	Poland; 52.3855, 20.1954
MT216296	ODOPL014-18	OdoPL14	BOLD:AAE5570	627[0n]	05-Oct-2018	*Ischnura elegans*	larvae	Lodz	Grzegorz Tonczyk	Poland; 52.3855, 20.1954
MT216288	ODOPL015-18	OdoPL15	BOLD:AAE5570	626[0n]	05-Oct-2018	*Ischnura elegans*	larvae	Lodz	Grzegorz Tonczyk	Poland; 52.836, 18.9037
MT216290	ODOPL016-18	OdoPL16	BOLD:AAE5570	627[0n]	05-Oct-2018	*Ischnura elegans*	larvae	Lodz	Grzegorz Tonczyk	Poland; 52.836, 18.9037
MW490314	PLSW013-20	ODOPL_147	BOLD:AAE5570	658[0n]	12-Jun-2019	*Ischnura elegans*	adult	Lodz	Grzegorz Tonczyk	Poland; 54.112, 23.043
MW490371	PLSW014-20	ODOPL_148	BOLD:AAE5570	658[0n]	12-Jun-2019	*Ischnura elegans*	adult	Lodz	Grzegorz Tonczyk	Poland; 54.112, 23.043
MW490463	PLSW018-20	ODOPL_152	BOLD:AAE5570	658[0n]	12-Jun-2019	*Ischnura elegans*	adult	Lodz	Grzegorz Tonczyk	Poland; 54.112, 23.043
MT298471	ZPLOD454-20	MIB:ZPL:08254	BOLD:AAE5570	658[0n]		*Ischnura genei*	Adult	Milano Bicocca	Giacomo Assandri	Italy; 37.1, 15.3
MT298472	ZPLOD457-20	MIB:ZPL:08257	BOLD:AAE5570	658[0n]		*Ischnura genei*	Adult	Milano Bicocca	Giacomo Assandri	Italy; 37.9, 12.7
MT298470	ZPLOD452-20	MIB:ZPL:08252	BOLD:AAE5570	658[0n]		*Ischnura genei*	Adult	Milano Bicocca	Giacomo Assandri	Italy; 40.7, 9
HM901893	FBAQU537-10	BC ZSM AQU 00442	BOLD:AAE5571	521[0n]	08-Dec-2009	*Ischnura pumilio*	adult	SNSB-ZSM	F. Weihrauch	Germany; 47.895, 12.167
HM901866	FBAQU496-10	BC ZSM AQU 00401	BOLD:AAE5571	614[0n]	24-Aug-2009	*Ischnura pumilio*	adult	SNSB-ZSM	S. V. Ober	Germany; 48.107, 11.458
HM422049	FBAQU315-09	BC ZSM AQU 00315	BOLD:AAE5571	658[0n]	20-May-2009	*Ischnura pumilio*	adult	SNSB-ZSM	M. Schoen	Germany; 48.302, 11.817
HM901894	FBAQU538-10	BC ZSM AQU 00443	BOLD:AAE5571	614[0n]	31-Jul-2009	*Ischnura pumilio*	adult	SNSB-ZSM	F. Weihrauch	Germany; 48.72, 11.676
MT298477	ZPLOD844-20	MIB:ZPL:08644	BOLD:AAE5570	658[0n]		*Ischnura saharensis*	Adult	Milano Bicocca	Giacomo Assandri	Morocco; 28.5, -10
MT298478	ZPLOD845-20	MIB:ZPL:08645	BOLD:AAE5570	658[0n]		*Ischnura saharensis*	Adult	Milano Bicocca	Giacomo Assandri	Morocco; 29.1, -10.3
MW490256	EDF015-18	Odon224	BOLD:ADC3442	658[0n]	07-Jun-2018	*Lestes barbarus*	adult	NHMW	Stephan Koblmueller	Austria; 47.719, 17.067
MW490253	EDF012-18	Odon191	BOLD:ADC3442	658[0n]	05-Jun-2018	*Lestes barbarus*	adult	NHMW	Stephan Koblmueller	Austria; 47.77, 16.88
MW490418	PLSW055-20	ODOPL_189	BOLD:ADC3442	658[0n]	15-Jul-2019	*Lestes barbarus*	adult	Lodz	Grzegorz Tonczyk	Poland; 51.717, 19.341
MW490081	RODI025-20	ROLE-LB-2	BOLD:ADC3442	632[0n]	18-Jul-2016	*Lestes barbarus*	adult	Babes-Bolyai	Anna Denes	Romania; 45.333, 29.489
MW490161	RODI026-20	ROLE-LB-3	BOLD:ADC3442	626[0n]	18-Jul-2016	*Lestes barbarus*	adult	Babes-Bolyai	Anna Denes	Romania; 45.333, 29.489
MW490496	PLSW056-20	ODOPL_190	BOLD:AEC4388	612[1n]	15-Jul-2019	*Lestes dryas*	adult	Lodz	Grzegorz Tonczyk	Poland; 51.717, 19.341
MW490127	PLSW057-20	ODOPL_191	BOLD:AEC4388	658[0n]	15-Jul-2019	*Lestes dryas*	adult	Lodz	Grzegorz Tonczyk	Poland; 51.717, 19.341
MW490338	PLSW058-20	ODOPL_192	BOLD:AEC4388	658[1n]	15-Jul-2019	*Lestes dryas*	adult	Lodz	Grzegorz Tonczyk	Poland; 51.717, 19.341
MW490319	EDF014-18	Odon222	BOLD:ADC3318	658[1n]	07-Jun-2018	*Lestes macrostigma*	adult	NHMW	Stephan Koblmueller	Austria; 47.719, 17.067
MW490249	EDF013-18	Odon197	BOLD:ADC3318	658[0n]	05-Jun-2018	*Lestes macrostigma*	adult	NHMW	Stephan Koblmueller	Austria; 47.77, 16.87

GenBank #	Process ID	Sample ID	BIN	Seq. Length	Collection Date	Identification	Life Stage	Institution (short)	Identifier	country; GPS
MW490393	GBEPT898-14	GBOL05468	BOLD:ACP4984	615[1n]	03-Jul-2014	*Lestes sponsa*	adult	SNSB-ZSM	Michael Franzen	Germany; 47.791, 12.112
MW490191	GBODO018-18	GBOL 20141	BOLD:ACP4984	314[1n]	27-Jul-2018	*Lestes sponsa*	adult	SNSB-ZSM	Stefan Koch	Germany; 47.8252, 11.3012
MW490095	GBODO133-18	GBOL 20256	BOLD:ACP4984	312[2n]	27-Jun-2018	*Lestes sponsa*	adult	SNSB-ZSM	Stefan Koch	Germany; 47.8252, 11.3012
MW490217	GBODO123-18	GBOL 20246	BOLD:ACP4984	318[2n]	19-Jul-2018	*Lestes sponsa*	adult	SNSB-ZSM	Stefan Koch	Germany; 47.9229, 11.5085
MW490229	TRDOD048-14	TRD-ODO66	BOLD:ACP4984	658[0n]	01-Aug-2014	*Lestes sponsa*	adult	NTNU	Jon K. Skei	Norway; 63.322, 10.652
MW490394	TRDOD049-14	TRD-ODO67	BOLD:ACP4984	658[0n]	01-Aug-2014	*Lestes sponsa*	adult	NTNU	Jon K. Skei	Norway; 63.322, 10.652
MW490108	TRDOD030-14	TRD-ODO48	BOLD:ACP4984	658[0n]	19-Jul-2014	*Lestes sponsa*	adult	NTNU	Jon K. Skei	Norway; 63.526, 11.051
MW490295	TRDOD031-14	TRD-ODO49	BOLD:ACP4984	658[0n]	22-Jul-2014	*Lestes sponsa*	adult	NTNU	Jon K. Skei	Norway; 63.548, 10.996
MW490407	PLSW059-20	ODOPL_193	BOLD:ACP4984	608[1n]	14-Jul-2019	*Lestes sponsa*	adult	Lodz	Grzegorz Tonczyk	Poland; 51.581, 19.219
MW490207	PLSW061-20	ODOPL_195	BOLD:ACP4984	658[0n]	14-Jul-2019	*Lestes sponsa*	adult	Lodz	Grzegorz Tonczyk	Poland; 51.581, 19.219
MW490092	GBODO039-18	GBOL 20162	BOLD:ACG0123	318[0n]	05-Sep-2018	*Lestes virens*	adult	SNSB-ZSM	Stefan Koch	Germany; 47.6668, 11.0506
MW490144	GBODO053-18	GBOL 20176	BOLD:ACG0123	318[0n]	05-Sep-2018	*Lestes virens*	adult	SNSB-ZSM	Stefan Koch	Germany; 47.6668, 11.0506
MW490519	GBODO073-18	GBOL 20196	BOLD:ACG0123	318[0n]	12-Jul-2018	*Lestes virens*	adult	SNSB-ZSM	Stefan Koch	Germany; 47.6668, 11.0506
MW490098	GBODO075-18	GBOL 20198	BOLD:ACG0123	318[0n]	12-Jul-2018	*Lestes virens*	adult	SNSB-ZSM	Stefan Koch	Germany; 47.6668, 11.0506
MW490391	GBODO090-18	GBOL 20213	BOLD:ACG0123	318[0n]	12-Jul-2018	*Lestes virens*	adult	SNSB-ZSM	Stefan Koch	Germany; 47.6668, 11.0506
MW490552	ODOPL017-19	OdoPL17	BOLD:ACG0123	634[0n]	14-Oct-2018	*Lestes virens*	adult	Lodz	Grzegorz Tonczyk	Poland; 51.5814, 19.2188
MW490534	ODOPL018-19	OdoPL18	BOLD:ACG0123	631[0n]	14-Oct-2018	*Lestes virens*	adult	Lodz	Grzegorz Tonczyk	Poland; 51.5814, 19.2188
MW490556	ODOPL019-19	OdoPL19	BOLD:ACG0123	636[0n]	14-Oct-2018	*Lestes virens*	adult	Lodz	Grzegorz Tonczyk	Poland; 51.5814, 19.2188
MW490566	FBAQU1427-13	GBOL00271	BOLD:ACG0123	620[0n]	23-Aug-2011	*Lestes virens vestalis*	adult	SNSB-ZSM	Stefan Koch	Germany; 47.6291, 11.1713
MW490326	GBEPT913-14	GBOL05483	BOLD:ACG0123	622[0n]	03-Jul-2014	*Lestes virens vestalis*	adult	SNSB-ZSM	Michael Franzen	Germany; 47.791, 12.112
MW490561	GBEPT914-14	GBOL05484	BOLD:ACG0123	619[0n]	03-Jul-2014	*Lestes virens vestalis*	adult	SNSB-ZSM	Michael Franzen	Germany; 47.791, 12.112
MW490348	EDF004-18	NOaS1-2019_Odo0182	BOLD:ADR0815	658[0n]	21-Jun-2018	*Leucorrhinia albifrons*	adult	NHMW	Iris Fischer	Austria; ,
MW490482	EDF005-18	NOaS1-2019_Odo0183	BOLD:ADR0815	658[0n]	21-Jun-2018	*Leucorrhinia albifrons*	adult	NHMW	Iris Fischer	Austria; ,
MW490080	PLSW021-20	ODOPL_155	BOLD:ADR0815	658[0n]	09-Jul-2019	*Leucorrhinia albifrons*	adult	Lodz	Grzegorz Tonczyk	Poland; 54.03, 23.052
MW490427	EDF006-18	NOaS1-2019_Odo0185	BOLD:ADC4475	658[0n]	26-Jun-2018	*Leucorrhinia caudalis*	adult	NHMW	Iris Fischer	Austria; ,
MW490479	EDF011-18	Odon176	BOLD:ADC4475	658[0n]	19-May-2018	*Leucorrhinia caudalis*	adult	NHMW	Gernot Kunz	Austria; 48.194, 16.484
MW490110	GBEPT900-14	GBOL05470	BOLD:AAJ2437	658[0n]	03-Jul-2014	*Leucorrhinia dubia*	adult	SNSB-ZSM	Michael Franzen	Germany; 47.791, 12.112
MW490483	GBEPT901-14	GBOL05471	BOLD:AAJ2437	654[1n]	03-Jul-2014	*Leucorrhinia dubia*	adult	SNSB-ZSM	Michael Franzen	Germany; 47.791, 12.112
MW490132	GBODO036-18	GBOL 20159	BOLD:AAJ2437	658[7n]	27-Jun-2018	*Leucorrhinia dubia*	adult	SNSB-ZSM	Stefan Koch	Germany; 47.8252, 11.3012
MW490549	GBODO136-18	GBOL 20259	BOLD:AAJ2437	318[0n]	27-Jun-2018	*Leucorrhinia dubia*	adult	SNSB-ZSM	Stefan Koch	Germany; 47.8252, 11.3012
HM901868	FBAQU501-10	BC ZSM AQU 00406	BOLD:AAJ2437	599[0n]	16-Jul-2009	*Leucorrhinia dubia*	adult	SNSB-ZSM	S. V. Ober	Germany; 47.909, 11.523
HM901867	FBAQU500-10	BC ZSM AQU 00405	BOLD:AAJ2437	600[0n]	20-May-2009	*Leucorrhinia dubia*	adult	SNSB-ZSM	S. V. Ober	Germany; 48.046, 11.467
MW490177	TRDOD009-14	TRD-ODO27	BOLD:AAJ2437	658[0n]	01-Jul-2014	*Leucorrhinia dubia*	adult	NTNU	Jon K. Skei	Norway; 63.375, 10.292
MW490171	TRDOD010-14	TRD-ODO28	BOLD:AAJ2437	658[0n]	07-Jul-2014	*Leucorrhinia dubia*	adult	NTNU	Jon K. Skei	Norway; 63.476, 11.244
MW490541	TRDOD012-14	TRD-ODO30	BOLD:AAJ2437	658[0n]	09-Jul-2014	*Leucorrhinia dubia*	adult	NTNU	Jon K. Skei	Norway; 63.54, 10.989
MW490251	TRDOD011-14	TRD-ODO29	BOLD:AAJ2437	658[0n]	27-Jun-2014	*Leucorrhinia dubia*	adult	NTNU	Jon K. Skei	Norway; 63.663, 10.943
MW490277	ODOPL129-19	OdoPL129	BOLD:AAJ2437	541[0n]	17-Nov-2018	*Leucorrhinia dubia*	larvae	Lodz	Grzegorz Tonczyk	Poland; 51.5799, 19.2154
MW490303	ODOPL099-19	OdoPL99	BOLD:AAJ2437	635[0n]	04-Nov-2018	*Leucorrhinia dubia*	larvae	Lodz	Grzegorz Tonczyk	Poland; 51.6201, 19.3112
MW490558	GBODO026-18	GBOL 20149	BOLD:ADC3719	318[1n]	27-Jul-2018	*Leucorrhinia pectoralis*	adult	SNSB-ZSM	Stefan Koch	Germany; 47.9229, 11.5085
MW490096	ODOPL024-19	OdoPL24	BOLD:ADC3719	625[0n]	14-Oct-2018	*Leucorrhinia pectoralis*	larvae	Lodz	Grzegorz Tonczyk	Poland; 51.5814, 19.2188
MW490297	PLSW051-20	ODOPL_185	BOLD:ADC3719	650[0n]	12-Jun-2019	*Leucorrhinia pectoralis*	adult	Lodz	Grzegorz Tonczyk	Poland; 54.112, 23.043

GenBank #	Process ID	Sample ID	BIN	Seq. Length	Collection Date	Identification	Life Stage	Institution (short)	Identifier	country; GPS
MW490443	PLSW052-20	ODOPL_186	BOLD:ADC3719	658[0n]	12-Jun-2019	*Leucorrhinia pectoralis*	adult	Lodz	Grzegorz Tonczyk	Poland; 54.112, 23.043
MW490500	PLSW053-20	ODOPL_187	BOLD:ADC3719	658[1n]	12-Jun-2019	*Leucorrhinia pectoralis*	adult	Lodz	Grzegorz Tonczyk	Poland; 54.112, 23.043
JN991193	GBA8708-12	JN991193	BOLD:ADC3719	467[0n]		*Leucorrhinia pectoralis*		GenBank, NCBI	0	Sweden; ,
MW490160	TRDOD013-14	TRD-ODO31	BOLD:ADC1709	658[0n]	09-Jul-2014	*Leucorrhinia rubicunda*	adult	NTNU	Jon K. Skei	Norway; 63.54, 10.989
HM901869	FBAQU502-10	BC ZSM AQU 00407	BOLD:AAJ2758	658[0n]	26-May-2009	*Libellula depressa*	adult	SNSB-ZSM	S. V. Ober	Germany; 48.046, 11.467
MW490234	GBODO145-18	GBOL 20268	BOLD:AAJ2758	658[0n]	12-May-2018	*Libellula depressa*	adult	SNSB-ZSM	Stefan Koch	Germany; 48.0616, 10.6428
MW490379	PLSW146-20	ODOPL_233	BOLD:AAJ2758	658[0n]	07-Aug-2019	*Libellula depressa*	larvae	Lodz	Grzegorz Tonczyk	Poland; 49.429, 21.859
MW490242	PLSW147-20	ODOPL_234	BOLD:AAJ2758	658[0n]	07-Aug-2019	*Libellula depressa*	larvae	Lodz	Grzegorz Tonczyk	Poland; 49.429, 21.859
MW490395	PLSW148-20	ODOPL_235	BOLD:AAJ2758	649[0n]	07-Aug-2019	*Libellula depressa*	larvae	Lodz	Grzegorz Tonczyk	Poland; 49.429, 21.859
MW490141	RODI027-20	ROLI-LD-1	BOLD:AAJ2758	658[0n]	11-Jul-2016	*Libellula depressa*	adult	Babes-Bolyai	Anna Denes	Romania; 46.63, 25.591
MW490258	GBEPT944-14	GBOL05514	BOLD:ACP3530	658[0n]	13-Jun-2014	*Libellula fulva*	adult	SNSB-ZSM	Michael Franzen	Austria; 47.931, 16.746
MW490378	GBODO006-18	GBOL 20129	BOLD:ACP3530	318[0n]	21-May-2018	*Libellula fulva*	adult	SNSB-ZSM	Stefan Koch	Germany; 47.7561, 11.3698
MW490376	PLSW033-20	ODOPL_167	BOLD:ACP3530	658[0n]	09-Jul-2019	*Libellula fulva*	adult	Lodz	Grzegorz Tonczyk	Poland; 54.03, 23.052
MW490523	PLSW034-20	ODOPL_168	BOLD:ACP3530	658[0n]	09-Jul-2019	*Libellula fulva*	adult	Lodz	Grzegorz Tonczyk	Poland; 54.03, 23.052
MW490333	PLSW035-20	ODOPL_169	BOLD:ACP3530	658[0n]	09-Jul-2019	*Libellula fulva*	adult	Lodz	Grzegorz Tonczyk	Poland; 54.03, 23.052
MW490361	GBODO001-18	GBOL 20124	BOLD:AAB5337	321[1n]	21-May-2018	*Libellula quadrimaculata*	adult	SNSB-ZSM	Stefan Koch	Germany; 47.7561, 11.3698
MW490439	GBODO002-18	GBOL 20125	BOLD:AAB5337	318[0n]	21-May-2018	*Libellula quadrimaculata*	adult	SNSB-ZSM	Stefan Koch	Germany; 47.7561, 11.3698
MW490546	GBODO011-18	GBOL 20134	BOLD:AAB5337	309[0n]	27-Jul-2018	*Libellula quadrimaculata*	adult	SNSB-ZSM	Stefan Koch	Germany; 47.8252, 11.3012
HM901870	FBAQU503-10	BC ZSM AQU 00408	BOLD:AAB5337	618[0n]	25-May-2009	*Libellula quadrimaculata*	adult	SNSB-ZSM	S. V. Ober	Germany; 48.107, 11.458
MW490356	GODO043-19	ZFMK-TIS-2616203	BOLD:AAB5337	658[1n]	30-May-2018	*Libellula quadrimaculata*	adult	Potsdam	Udo Rothe	Germany; 52.4559, 12.693
MW490231	GODO044-19	ZFMK-TIS-2616215	BOLD:AAB5337	658[0n]	01-Jun-1992	*Libellula quadrimaculata*	adult	Potsdam	0	Germany; 53.2962, 12.0863
MW490170	TRDOD057-14	TRD-ODO75	BOLD:AAB5337	658[0n]	24-Jul-2010	*Libellula quadrimaculata*	adult	NTNU	Jon K. Skei	Norway; 63.322, 10.652
MW490398	TRDOD051-14	TRD-ODO69	BOLD:AAB5337	658[0n]	03-Aug-2014	*Libellula quadrimaculata*	adult	NTNU	Jon K. Skei	Norway; 63.536, 11.005
MW490404	TRDOD028-14	TRD-ODO46	BOLD:AAB5337	658[0n]	20-Jul-2014	*Libellula quadrimaculata*	adult	NTNU	Jon K. Skei	Norway; 63.549, 11.038
MW490163	TRDOD003-14	TRD-ODO21	BOLD:AAB5337	658[0n]	27-Jun-2014	*Libellula quadrimaculata*	adult	NTNU	Jon K. Skei	Norway; 63.603, 11.159
MW490083	ODOPL122-19	OdoPL122	BOLD:AAB5337	637[0n]	17-Nov-2018	*Libellula quadrimaculata*	larvae	Lodz	Grzegorz Tonczyk	Poland; 51.5799, 19.2154
MW490349	ODOPL096-19	OdoPL96	BOLD:AAB5337	644[0n]	04-Nov-2018	*Libellula quadrimaculata*	larvae	Lodz	Grzegorz Tonczyk	Poland; 51.6201, 19.3112
MW490317	ODOPL097-19	OdoPL97	BOLD:AAB5337	643[0n]	04-Nov-2018	*Libellula quadrimaculata*	larvae	Lodz	Grzegorz Tonczyk	Poland; 51.6201, 19.3112
MW490542	ODOPL098-19	OdoPL98	BOLD:AAB5337	639[0n]	04-Nov-2018	*Libellula quadrimaculata*	larvae	Lodz	Grzegorz Tonczyk	Poland; 51.6201, 19.3112
MW490564	PLSW030-20	ODOPL_164	BOLD:AAB5337	658[0n]	09-Jul-2019	*Libellula quadrimaculata*	adult	Lodz	Grzegorz Tonczyk	Poland; 54.03, 23.052
MW490365	PLSW031-20	ODOPL_165	BOLD:AAB5337	658[0n]	09-Jul-2019	*Libellula quadrimaculata*	adult	Lodz	Grzegorz Tonczyk	Poland; 54.03, 23.052
MW490353	PLSW032-20	ODOPL_166	BOLD:AAB5337	658[0n]	09-Jul-2019	*Libellula quadrimaculata*	adult	Lodz	Grzegorz Tonczyk	Poland; 54.03, 23.052
MT298523	ZPLOD556-20	MIB:ZPL:08356	BOLD:ACW0675	658[0n]		*Lindenia tetraphylla*	adult	Milano Bicocca	Giacomo Assandri	Italy; 37.7, 12.8
MT298525	ZPLOD550-20	MIB:ZPL:08350	BOLD:ACW0675	658[0n]		*Lindenia tetraphylla*	adult	Giacomo Assandri	Giacomo Assandri	Italy; 40, 8.5
MT298527	ZPLOD555-20	MIB:ZPL:08355	BOLD:ACW0675	658[0n]		*Lindenia tetraphylla*	adult	Milano Bicocca	Giacomo Assandri	Italy; 41.6, 14.9
MT298526	ZPLOD553-20	MIB:ZPL:08353	BOLD:ACW0675	658[0n]		*Lindenia tetraphylla*	adult	Milano Bicocca	Giacomo Assandri	Italy; 42, 14.8
MT298524	ZPLOD552-20	MIB:ZPL:08352	BOLD:ACW0675	658[0n]		*Lindenia tetraphylla*	adult	Milano Bicocca	Giacomo Assandri	Italy; 43.1, 12.2
MW490213	FBAQU1428-13	GBOL00272	BOLD:AAC3125	651[0n]	28-Jun-2011	*Nehalennia speciosa*	adult	SNSB-ZSM	Stefan Koch	Germany; 47.6328, 10.5381
MW490182	GBEPT895-14	GBOL05465	BOLD:AAC3125	658[0n]	03-Jul-2014	*Nehalennia speciosa*	adult	SNSB-ZSM	Michael Franzen	Germany; 47.791, 12.112
MW490563	GBEPT896-14	GBOL05466	BOLD:AAC3125	658[0n]	03-Jul-2014	*Nehalennia speciosa*	adult	SNSB-ZSM	Michael Franzen	Germany; 47.791, 12.112
MW490511	GBEPT897-14	GBOL05467	BOLD:AAC3125	658[0n]	03-Jul-2014	*Nehalennia speciosa*	adult	SNSB-ZSM	Michael Franzen	Germany; 47.791, 12.112

GenBank #	Process ID	Sample ID	BIN	Seq. Length	Collection Date	Identification	Life Stage	Institution (short)	Identifier	country; GPS
MW490088	GBEPT942-14	GBOL05512	BOLD:AAC3125	658[0n]	03-Jul-2014	*Nehalennia speciosa*	adult	SNSB-ZSM	Michael Franzen	Germany; 47.791, 12.112
HM901895	FBAQU541-10	BC ZSM AQU 00446	BOLD:AAC3125	610[0n]	16-Jul-2009	*Nehalennia speciosa*	adult	SNSB-ZSM	F. Weihrauch	Germany; 47.895, 12.167
MW490435	PLSW004-20	ODOPL_138	BOLD:AAC3125	658[0n]	14-Jun-2019	*Nehalennia speciosa*	adult	Lodz	Grzegorz Tonczyk	Poland; 54.089, 23.022
MW490425	PLSW005-20	ODOPL_139	BOLD:AAC3125	658[0n]	14-Jun-2019	*Nehalennia speciosa*	adult	Lodz	Grzegorz Tonczyk	Poland; 54.089, 23.022
MW490157	PLSW006-20	ODOPL_140	BOLD:AAC3125	658[0n]	14-Jun-2019	*Nehalennia speciosa*	adult	Lodz	Grzegorz Tonczyk	Poland; 54.089, 23.022
MW490255	GBEPT922-14	GBOL05492	BOLD:AAE5061	593[2n]	11-Jun-2014	*Onychogomphus forcipatus*	adult	SNSB-ZSM	Michael Franzen	Austria; 48.019, 16.166
HM901898	FBAQU544-10	BC ZSM AQU 00449	BOLD:AAE5061	614[0n]	31-Jul-2009	*Onychogomphus forcipatus*	larvae	SNSB-ZSM	Monika Hess	Germany; 47.741, 11.12
HM901897	FBAQU543-10	BC ZSM AQU 00448	BOLD:AAE5061	536[1n]	04-Nov-2009	*Onychogomphus forcipatus*	larvae	SNSB-ZSM	Monika Hess	Germany; 48.043, 12.798
HM901871	FBAQU504-10	BC ZSM AQU 00409	BOLD:AAE5061	614[0n]	18-Jun-2009	*Onychogomphus forcipatus*	adult	SNSB-ZSM	S. V. Ober	Germany; 48.236, 11.505
HM901896	FBAQU542-10	BC ZSM AQU 00447	BOLD:AAE5061	569[0n]	31-Aug-2009	*Onychogomphus forcipatus*	larvae	SNSB-ZSM	Monika Hess	Germany; 49.038, 12.187
MW459447	GODO002-18	ZFMK-TIS-2534021	BOLD:AAE5061	658[0n]	10-Aug-2016	*Onychogomphus forcipatus*		ZFMK	Simone Chapuis	Germany; 50.8, 7.17
MW459820	GODO003-18	ZFMK-TIS-2534116	BOLD:AAE5061	658[0n]	10-Aug-2016	*Onychogomphus forcipatus*		ZFMK	Simone Chapuis	Germany; 50.8, 7.32
MW459607	GODO004-18	ZFMK-TIS-2534156	BOLD:AAE5061	658[0n]	10-Aug-2016	*Onychogomphus forcipatus*		ZFMK	Simone Chapuis	Germany; 50.8, 7.32
MT298542	ZPLOD564-20	MIB:ZPL:08364	BOLD:AAE5061	658[0n]		*Onychogomphus forcipatus*	adult	Milano Bicocca	Giacomo Assandri	Italy; 38, 15.3
MT298543	ZPLOD565-20	MIB:ZPL:08365	BOLD:AAE5061	658[0n]		*Onychogomphus forcipatus*	adult	Milano Bicocca	Giacomo Assandri	Italy; 38, 15.3
MT298536	ZPLOD578-20	MIB:ZPL:08378	BOLD:AAE5061	658[0n]		*Onychogomphus forcipatus*	adult	Giacomo Assandri	Giacomo Assandri	Italy; 40.6, 16.8
MT298538	ZPLOD580-20	MIB:ZPL:08380	BOLD:AAE5061	658[0n]		*Onychogomphus forcipatus*	adult	Milano Bicocca	Giacomo Assandri	Italy; 41.9, 15
MT298537	ZPLOD579-20	MIB:ZPL:08379	BOLD:AAE5061	658[0n]		*Onychogomphus forcipatus*	adult	Giacomo Assandri	Giacomo Assandri	Italy; 44.5, 10.9
MT298535	ZPLOD576-20	MIB:ZPL:08376	BOLD:AAE5061	658[0n]		*Onychogomphus forcipatus*	adult	Giacomo Assandri	Giacomo Assandri	Italy; 45.8, 9.4
MT298532	ZPLOD569-20	MIB:ZPL:08369	BOLD:AAE5061	658[0n]		*Onychogomphus forcipatus*	adult	Giacomo Assandri	Giacomo Assandri	Italy; 45.9, 11
MT298531	ZPLOD568-20	MIB:ZPL:08368	BOLD:AAE5061	658[0n]		*Onychogomphus forcipatus*	adult	Giacomo Assandri	Giacomo Assandri	Italy; 46, 11.3
MT298540	ZPLOD563-20	MIB:ZPL:08363	BOLD:AAE5061	658[0n]		*Onychogomphus forcipatus*	adult	Milano Bicocca	Giacomo Assandri	Italy; 46, 13.6
MT298533	ZPLOD570-20	MIB:ZPL:08370	BOLD:AAE5061	658[0n]		*Onychogomphus forcipatus*	adult	Giacomo Assandri	Giacomo Assandri	Italy; 46.1, 11.1
MT298534	ZPLOD571-20	MIB:ZPL:08371	BOLD:AAE5061	658[0n]		*Onychogomphus forcipatus*	adult	Giacomo Assandri	Giacomo Assandri	Italy; 46.1, 11.1
MT298544	ZPLOD566-20	MIB:ZPL:08366	BOLD:AAE5061	658[0n]		*Onychogomphus forcipatus*	adult	Giacomo Assandri	Giacomo Assandri	Italy; 46.1, 11.2
MT298541	ZPLOD562-20	MIB:ZPL:08362	BOLD:AAE5061	658[0n]		*Onychogomphus forcipatus*	adult	Milano Bicocca	Giacomo Assandri	Italy; 46.2, 12.9
MW490489	PLSW041-20	ODOPL_175	BOLD:AAE5061	652[0n]	13-Jun-2019	*Onychogomphus forcipatus*	adult	Lodz	Grzegorz Tonczyk	Poland; 54.097, 23.063
MW490192	PLSW042-20	ODOPL_176	BOLD:AAE5061	658[0n]	13-Jun-2019	*Onychogomphus forcipatus*	adult	Lodz	Grzegorz Tonczyk	Poland; 54.097, 23.063
MW490097	PLSW043-20	ODOPL_177	BOLD:AAE5061	658[0n]	13-Jun-2019	*Onychogomphus forcipatus*	adult	Lodz	Grzegorz Tonczyk	Poland; 54.097, 23.063
MT298548	ZPLOD581-20	MIB:ZPL:08381	BOLD:ADC3114	658[0n]		*Onychogomphus uncatus*	adult	Milano Bicocca	Giacomo Assandri	Italy; 37.1, 15
MT298545	ZPLOD587-20	MIB:ZPL:08387	BOLD:ADC3114	658[0n]		*Onychogomphus uncatus*	adult	Milano Bicocca	Giacomo Assandri	Italy; 41.6, 14.6
MT298547	ZPLOD582-20	MIB:ZPL:08382	BOLD:ADC3114	658[0n]		*Onychogomphus uncatus*	adult	Milano Bicocca	Giacomo Assandri	Italy; 44.5, 8.8
MT298546	ZPLOD585-20	MIB:ZPL:08385	BOLD:ADC3114	658[0n]		*Onychogomphus uncatus*	adult	Giacomo Assandri	Giacomo Assandri	Italy; 45.3, 9
MW490320	GBEPT949-14	GBOL05519	BOLD:ACP4340	658[0n]	14-Jun-2014	*Ophiogomphus cecilia*	adult	SNSB-ZSM	Michael Franzen	Austria; 48.132, 16.95
MW490155	GBEPT1622-14	GBOL-08729	BOLD:ACP4340	658[0n]	17-Jul-2013	*Ophiogomphus cecilia*	larvae	SNSB-ZSM	Tobias Windmaisser	Germany; 49.072, 12.297
MW490377	GBMIX417-14	GBOL09709	BOLD:ACP4340	409[0n]	17-Jul-2013	*Ophiogomphus cecilia*	larvae	SNSB-ZSM	Christoph D. Schubart	Germany; 49.072, 12.297
MW490239	GBMIX418-14	GBOL09710	BOLD:ACP4340	658[0n]	17-Jul-2013	*Ophiogomphus cecilia*	larvae	SNSB-ZSM	Christoph D. Schubart	Germany; 49.072, 12.297
MW490100	GBMIX667-14	GBOL09769	BOLD:ACP4340	409[0n]	13-Aug-2014	*Ophiogomphus cecilia*		SNSB-ZSM	Lars Hendrich	Germany; 51.364, 14.446
MW490505	GBEPT1043-14	GBOL06278	BOLD:ACP4340	658[0n]	05-Jun-2014	*Ophiogomphus cecilia*	larvae	SNSB-ZSM	Lars Hendrich	Germany; 51.503, 14.362
MW490087	ODOPL076-19	OdoPL76	BOLD:ACP4340	636[0n]	02-Nov-2018	*Ophiogomphus cecilia*	larvae	Lodz	Grzegorz Tonczyk	Poland; 51.3164, 19.9037
MW490115	ODOPL078-19	OdoPL78	BOLD:ACP4340	644[0n]	02-Nov-2018	*Ophiogomphus cecilia*	larvae	Lodz	Grzegorz Tonczyk	Poland; 51.3164, 19.9037

GenBank #	Process ID	Sample ID	BIN	Seq. Length	Collection Date	Identification	Life Stage	Institution (short)	Identifier	country; GPS
MW490201	ODOPL107-19	OdoPL107	BOLD:ACP4340	642[0n]	11-Nov-2018	*Ophiogomphus cecilia*	larvae	Lodz	Grzegorz Tonczyk	Poland; 51.4938, 18.9853
MW490278	ODOPL039-19	OdoPL39	BOLD:ACP4340	641[0n]	10-Oct-2018	*Ophiogomphus cecilia*	larvae	Lodz	Grzegorz Tonczyk	Poland; 51.6184, 16.333
MW490238	EDF010-18	Odon32	BOLD:ACQ8102	658[0n]	05-Jun-2017	*Orthetrum albistylum*	adult	NHMW	Stephan Koblmueller	Austria; 47.07, 15.134
MW490175	GBODO059-18	GBOL 20182	BOLD:AAK5997	658[0n]	04-Jul-2018	*Orthetrum brunneum*	adult	SNSB-ZSM	Stefan Koch	Germany; 48.3305, 11.8047
MW490205	GBODO060-18	GBOL 20183	BOLD:AAK5997	658[2n]	04-Jul-2018	*Orthetrum brunneum*	adult	SNSB-ZSM	Stefan Koch	Germany; 48.3305, 11.8047
HM422050	FBAQU319-09	BC ZSM AQU 00319	BOLD:AAK5997	658[0n]	13-Jun-2009	*Orthetrum brunneum*	adult	SNSB-ZSM	M. Schoen	Germany; 48.339, 11.799
MW490085	PLSW062-20	ODOPL_196	BOLD:AAK5997	658[0n]	14-Jul-2019	*Orthetrum brunneum*	adult	Lodz	Grzegorz Tonczyk	Poland; 51.598, 19.272
MW490122	PLSW063-20	ODOPL_197	BOLD:AAK5997	658[0n]	14-Jul-2019	*Orthetrum brunneum*	adult	Lodz	Grzegorz Tonczyk	Poland; 51.598, 19.272
HM901872	FBAQU505-10	BC ZSM AQU 00410	BOLD:AAK5996	646[0n]	25-May-2009	*Orthetrum cancellatum*	adult	SNSB-ZSM	S. V. Ober	Germany; 48.107, 11.458
MW490392	GODO038-18	ZFMK-TIS-2531806	BOLD:AAK5996	658[0n]	15-Jun-2014	*Orthetrum cancellatum*	adult	ZFMK	Bjoern Rulik	Germany; 53.4048, 12.866
MW490293	ODOPL051-19	OdoPL51	BOLD:AAK5996	633[0n]	10-Oct-2018	*Orthetrum cancellatum*	larvae	Lodz	Grzegorz Tonczyk	Poland; 50.4118, 18.1075
MW490555	ODOPL052-19	OdoPL52	BOLD:AAK5996	633[0n]	11-Oct-2018	*Orthetrum cancellatum*	larvae	Lodz	Grzegorz Tonczyk	Poland; 50.4118, 18.1075
MW490506	ODOPL127-19	OdoPL127	BOLD:AAK5996	635[0n]	17-Nov-2018	*Orthetrum cancellatum*	larvae	Lodz	Grzegorz Tonczyk	Poland; 51.5799, 19.2154
MW490091	ODOPL128-19	OdoPL128	BOLD:AAK5996	623[0n]	17-Nov-2018	*Orthetrum cancellatum*	larvae	Lodz	Grzegorz Tonczyk	Poland; 51.5799, 19.2154
MW490246	PLSW027-20	ODOPL_161	BOLD:AAK5996	658[0n]	10-Jun-2019	*Orthetrum cancellatum*	adult	Lodz	Grzegorz Tonczyk	Poland; 54.022, 23.053
MW490292	PLSW028-20	ODOPL_162	BOLD:AAK5996	658[0n]	10-Jun-2019	*Orthetrum cancellatum*	adult	Lodz	Grzegorz Tonczyk	Poland; 54.022, 23.053
MW490129	PLSW029-20	ODOPL_163	BOLD:AAK5996	658[0n]	10-Jun-2019	*Orthetrum cancellatum*	adult	Lodz	Grzegorz Tonczyk	Poland; 54.022, 23.053
MT298569	ZPLOD846-20	MIB:ZPL:08646	BOLD:ABA9336	658[0n]		*Orthetrum chrysostigma*	adult	Milano Bicocca	Giacomo Assandri	Morocco; 28.5, -10
MW490285	GBODO033-18	GBOL 20156	BOLD:AAI2353	658[0n]	21-Jun-2018	*Orthetrum coerulescens*	adult	SNSB-ZSM	Stefan Koch	Germany; 48.1197, 10.4546
HM901873	FBAQU506-10	BC ZSM AQU 00411	BOLD:AAI2353	641[0n]	18-Jun-2009	*Orthetrum coerulescens*	adult	SNSB-ZSM	S. V. Ober	Germany; 48.236, 11.505
MW490386	GBODO057-18	GBOL 20180	BOLD:AAI2353	592[2n]	04-Jul-2018	*Orthetrum coerulescens*	adult	SNSB-ZSM	Stefan Koch	Germany; 48.3537, 11.775
HM422051	FBAQU320-09	BC ZSM AQU 00320	BOLD:AAI2353	658[0n]	17-Jun-2009	*Orthetrum coerulescens*	adult	SNSB-ZSM	M. Schoen	Germany; 48.376, 11.842
MW490473	GBEPT937-14	GBOL05507	BOLD:AAI2353	658[0n]	08-Jun-2014	*Orthetrum coerulescens*	adult	SNSB-ZSM	Michael Franzen	Germany; 48.378, 11.841
MW490263	GBEPT941-14	GBOL05511	BOLD:AAI2353	658[0n]	08-Jun-2014	*Orthetrum coerulescens*	adult	SNSB-ZSM	Michael Franzen	Germany; 48.378, 11.841
MW490525	ZMBN329-16	ZMBN-Ent14	BOLD:AAI2353	658[0n]	23-Jun-2016	*Orthetrum coerulescens*		Bergen	Steffen Roth	Norway; 59.7137, 5.2176
MW490508	PLSW047-20	ODOPL_181	BOLD:AAI2353	658[0n]	13-Jun-2019	*Orthetrum coerulescens*	adult	Lodz	Grzegorz Tonczyk	Poland; 54.103, 23.032
MW490384	PLSW048-20	ODOPL_182	BOLD:AAI2353	658[0n]	13-Jun-2019	*Orthetrum coerulescens*	adult	Lodz	Grzegorz Tonczyk	Poland; 54.103, 23.032
MW490520	PLSW049-20	ODOPL_183	BOLD:AAI2353	658[0n]	13-Jun-2019	*Orthetrum coerulescens*	adult	Lodz	Grzegorz Tonczyk	Poland; 54.103, 23.032
MT298585	ZPLOD663-20	MIB:ZPL:08463	BOLD:AEC4264	658[0n]	13-Jul-2019	*Orthetrum nitidinerve*	adult	Giacomo Assandri	Giacomo Assandri	Italy; 39.8, 9
MT298584	ZPLOD661-20	MIB:ZPL:08461	BOLD:AEC4264	658[0n]	09-Jul-2019	*Orthetrum nitidinerve*	adult	Giacomo Assandri	Giacomo Assandri	Italy; 40.2, 8.9
MT298588	ZPLOD667-20	MIB:ZPL:08467	BOLD:ABA9397	658[0n]		*Orthetrum trinacria*	adult	Milano Bicocca	Giacomo Assandri	Italy; 37.5, 14.9
MT298587	ZPLOD668-20	MIB:ZPL:08468	BOLD:ABA9397	658[0n]		*Orthetrum trinacria*	adult	Milano Bicocca	Giacomo Assandri	Italy; 37.7, 12.8
MT298586	ZPLOD665-20	MIB:ZPL:08465	BOLD:ABA9397	658[0n]		*Orthetrum trinacria*	adult	Giacomo Assandri	Giacomo Assandri	Italy; 40.8, 8.5
KX241515	GBMIN88646-17	KX241515	BOLD:ADC4889	692[0n]		*Oxygastra curtisii*		GenBank, NCBI	0	Italy; ,
MT298591	ZPLOD676-20	MIB:ZPL:08476	BOLD:ABW0140	658[0n]		*Paragomphus genei*	adult	Milano Bicocca	Giacomo Assandri	Italy; 37.6, 13.1
MT298592	ZPLOD675-20	MIB:ZPL:08475	BOLD:ABW0140	658[0n]		*Paragomphus genei*	adult	Giacomo Assandri	Giacomo Assandri	Italy; 40.3, 9.5
MT298593	ZPLOD674-20	MIB:ZPL:08474	BOLD:ABW0140	658[0n]		*Paragomphus genei*	adult	Giacomo Assandri	Giacomo Assandri	Italy; 40.7, 9
MW490186	GBODO161-18	GBOL 20284		318[1n]	12-May-2018	*Platycnemis pennipes*	adult	SNSB-ZSM	Stefan Koch	Germany; 48.0616, 10.6428
MW490179	GBODO055-18	GBOL 20178		315[1n]	04-Jul-2018	*Platycnemis pennipes*	adult	SNSB-ZSM	Stefan Koch	Germany; 48.3537, 11.775
MW490449	GODO023-18	ZFMK-TIS-2010623	BOLD:ACG0515	658[0n]	30-May-2017	*Platycnemis pennipes*	adult	ZFMK	T. Sellmeier	Germany; 50.777, 7.365
MW490180	GODO027-18	ZFMK-TIS-2010635	BOLD:ACG0515	658[0n]	27-Jun-2017	*Platycnemis pennipes*	adult	ZFMK	T. Sellmeier	Germany; 50.8, 7.585

GenBank #	Process ID	Sample ID	BIN	Seq. Length	Collection Date	Identification	Life Stage	Institution (short)	Identifier	country; GPS
MW490514	GODO030-18	ZFMK-TIS-2010638	BOLD:ACG0515	658[0n]	11-Jul-2017	*Platycnemis pennipes*	adult	ZFMK	T. Sellmeier	Germany; 50.8, 7.585
KF369498	ODOPH289-13	RMNH.INS.228274	BOLD:ACG0515	658[0n]		*Platycnemis pennipes*	adult	Leiden	0	Netherlands; ,
MW490280	PLSW079-20	ODOPL_213	BOLD:ACG0515	585[2n]	07-Aug-2019	*Platycnemis pennipes*	adult	Lodz	Grzegorz Tonczyk	Poland; 49.429, 21.859
MW490153	PLSW080-20	ODOPL_214	BOLD:ACG0515	643[2n]	07-Aug-2019	*Platycnemis pennipes*	adult	Lodz	Grzegorz Tonczyk	Poland; 49.429, 21.859
MW490351	PLSW015-20	ODOPL_149	BOLD:ACG0515	658[0n]	12-Jun-2019	*Platycnemis pennipes*	adult	Lodz	Grzegorz Tonczyk	Poland; 54.112, 23.043
MW490494	PLSW050-20	ODOPL_184	BOLD:ACG0515	658[0n]	12-Jun-2019	*Platycnemis pennipes*	adult	Lodz	Grzegorz Tonczyk	Poland; 54.112, 23.043
MW490465	GBUPS203-14	GBOL02853	BOLD:AAD5734	683[0n]		*Pyrrhosoma nymphula*	adult	SNSB-ZSM	Lars Hendrich	Germany; ,
MW490106	GBUPS204-14	GBOL02854	BOLD:AAD5734	684[0n]		*Pyrrhosoma nymphula*	adult	SNSB-ZSM	Lars Hendrich	Germany; ,
MW490308	GBODO098-18	GBOL 20221	BOLD:AAD5734	658[0n]	12-Jul-2018	*Pyrrhosoma nymphula*	adult	SNSB-ZSM	Stefan Koch	Germany; 47.6668, 11.0506
MW490339	GBODO152-18	GBOL 20275	BOLD:AAD5734	658[0n]	21-May-2018	*Pyrrhosoma nymphula*	adult	SNSB-ZSM	Stefan Koch	Germany; 47.7561, 11.3698
HM901876	FBAQU511-10	BC ZSM AQU 00416	BOLD:AAD5734	612[0n]	29-Jul-2009	*Pyrrhosoma nymphula*	adult	SNSB-ZSM	S. V. Ober	Germany; 47.766, 12.816
HM901899	FBAQU547-10	BC ZSM AQU 00452	BOLD:AAD5734	612[0n]	16-Sep-2009	*Pyrrhosoma nymphula*	larvae	SNSB-ZSM	Monika Hess	Germany; 47.896, 12.166
HM901875	FBAQU510-10	BC ZSM AQU 00415	BOLD:AAD5734	612[0n]	16-Jul-2009	*Pyrrhosoma nymphula*	adult	SNSB-ZSM	S. V. Ober	Germany; 47.909, 11.523
HM901874	FBAQU509-10	BC ZSM AQU 00414	BOLD:AAD5734	612[0n]	25-May-2009	*Pyrrhosoma nymphula*	adult	SNSB-ZSM	S. V. Ober	Germany; 48.107, 11.458
GU682172	FBAQU321-09	BC ZSM AQU 00321	BOLD:AAD5734	658[0n]	20-May-2009	*Pyrrhosoma nymphula*	adult	SNSB-ZSM	M. Schoen	Germany; 48.302, 11.817
MW490341	GBEPT916-14	GBOL05486	BOLD:AAD5734	658[0n]	22-May-2014	*Pyrrhosoma nymphula*	adult	SNSB-ZSM	Michael Franzen	Germany; 48.378, 11.841
MW490499	GBEPT917-14	GBOL05487	BOLD:AAD5734	658[0n]	22-May-2014	*Pyrrhosoma nymphula*	adult	SNSB-ZSM	Michael Franzen	Germany; 48.378, 11.841
GU682191	FBAQU202-09	BC ZSM AQU 00202	BOLD:AAD5734	658[0n]	07-Aug-2009	*Pyrrhosoma nymphula*	larvae	SNSB-ZSM	Monika Hess	Germany; 49.099, 13.155
MW490401	GODO026-18	ZFMK-TIS-2010626	BOLD:AAD5734	658[0n]	30-May-2017	*Pyrrhosoma nymphula*	adult	ZFMK	T. Sellmeier	Germany; 50.8, 7.585
MW490431	TRDOD015-14	TRD-ODO33	BOLD:AAD5734	658[0n]	05-Jun-2014	*Pyrrhosoma nymphula*	adult	NTNU	Jon K. Skei	Norway; 63.217, 10.307
MW490357	TRDOD014-14	TRD-ODO32	BOLD:AAD5734	658[0n]	01-Jul-2014	*Pyrrhosoma nymphula*	adult	NTNU	Jon K. Skei	Norway; 63.374, 10.286
MW490504	TRDOD070-14	TRD-ODO88	BOLD:AAD5734	658[0n]	12-Jul-2014	*Pyrrhosoma nymphula*	adult	NTNU	Jon K. Skei	Norway; 63.543, 11.033
MW490373	ODTRI002-14	TRD-ODO2	BOLD:AAD5734	621[0n]	29-Jul-2010	*Pyrrhosoma nymphula*	adult	NTNU	Jon K. Skei	Norway; 63.548, 10.996
MW490089	ODTRI003-14	TRD-ODO3	BOLD:AAD5734	624[0n]	15-Jun-2010	*Pyrrhosoma nymphula*	adult	NTNU	Jon K. Skei	Norway; 63.548, 10.996
MW490345	TRDOD071-14	TRD-ODO89	BOLD:AAD5734	658[0n]	22-Jul-2014	*Pyrrhosoma nymphula*	adult	NTNU	Jon K. Skei	Norway; 63.548, 10.996
MT298608	ZPLOD700-20	MIB:ZPL:08500	BOLD:AAE9139	658[0n]		*Selysiothemis nigra*	adult	Milano Bicocca	Giacomo Assandri	Italy; 37.5, 14.9
MT298607	ZPLOD703-20	MIB:ZPL:08503	BOLD:AAE9139	658[0n]		*Selysiothemis nigra*	adult	Milano Bicocca	Giacomo Assandri	Italy; 41.8, 15
MT298609	ZPLOD701-20	MIB:ZPL:08501	BOLD:AAE9139	658[0n]		*Selysiothemis nigra*	adult	Giacomo Assandri	Giacomo Assandri	Italy; 44.7, 12.2
MT298611	ZPLOD709-20	MIB:ZPL:08509	BOLD:ACP5227	658[0n]		*Somatochlora alpestris*	adult	Giacomo Assandri	Giacomo Assandri	Italy; 46.1, 9.6
MT298610	ZPLOD710-20	MIB:ZPL:08510	BOLD:ACP5227	658[0n]		*Somatochlora alpestris*	adult	Milano Bicocca	Giacomo Assandri	Italy; 46.4, 9.4
MT298613	ZPLOD706-20	MIB:ZPL:08506	BOLD:ACP5227	658[0n]		*Somatochlora alpestris*	adult	Milano Bicocca	Giacomo Assandri	Italy; 46.5, 11.1
MT298612	ZPLOD707-20	MIB:ZPL:08507	BOLD:ACP5227	658[0n]		*Somatochlora alpestris*	adult	Milano Bicocca	Giacomo Assandri	Italy; 46.5, 12.7
MW490105	TRDOD064-14	TRD-ODO82	BOLD:ACP7013	658[0n]	24-Jul-2010	*Somatochlora arctica*	adult	NTNU	Jon K. Skei	Norway; 63.321, 10.638
MW490328	TRDOD045-14	TRD-ODO63	BOLD:ACP7013	658[0n]	02-Aug-2014	*Somatochlora arctica*	adult	NTNU	Jon K. Skei	Norway; 63.541, 11.033
MW490527	TRDOD046-14	TRD-ODO64	BOLD:ACP7013	618[0n]	02-Aug-2014	*Somatochlora arctica*	adult	NTNU	Jon K. Skei	Norway; 63.541, 11.033
MW490146	TRDOD047-14	TRD-ODO65	BOLD:ACP7013	658[0n]	02-Aug-2014	*Somatochlora arctica*	adult	NTNU	Jon K. Skei	Norway; 63.541, 11.033
MW490131	GBODO081-18	GBOL 20204	BOLD:AEC6167	315[0n]	12-Jul-2018	*Somatochlora flavomaculata*	adult	SNSB-ZSM	Stefan Koch	Germany; 47.6668, 11.0506
MW490298	GBODO022-18	GBOL 20145	BOLD:AEC6167	315[0n]	27-Jul-2018	*Somatochlora flavomaculata*	adult	SNSB-ZSM	Stefan Koch	Germany; 47.8252, 11.3012
HM901900	FBAQU548-10	BC ZSM AQU 00453	BOLD:AEC6167	513[0n]	16-Jul-2009	*Somatochlora flavomaculata*	adult	SNSB-ZSM	F. Weihrauch	Germany; 47.895, 12.167
MW490082	GBODO027-18	GBOL 20150	BOLD:AEC6167	311[0n]	27-Jul-2018	*Somatochlora flavomaculata*	adult	SNSB-ZSM	Stefan Koch	Germany; 47.9229, 11.5085
MW490441	GBODO107-18	GBOL 20230	BOLD:AEC6167	315[0n]	19-Jul-2018	*Somatochlora flavomaculata*	adult	SNSB-ZSM	Stefan Koch	Germany; 47.9229, 11.5085

GenBank #	Process ID	Sample ID	BIN	Seq. Length	Collection Date	Identification	Life Stage	Institution (short)	Identifier	country; GPS
MW490367	GBODO121-18	GBOL 20244	BOLD:AEC6167	312[0n]	19-Jul-2018	*Somatochlora flavomaculata*	adult	SNSB-ZSM	Stefan Koch	Germany; 47.9229, 11.5085
MW490086	PLSW020-20	ODOPL_154	BOLD:AEC6167	658[0n]	21-Jun-2019	*Somatochlora flavomaculata*	adult	Lodz	Grzegorz Tonczyk	Poland; 50.015, 20.281
MW490211	PLSW084-20	ODOPL_218	BOLD:AEC6167	654[0n]	30-Jun-2019	*Somatochlora flavomaculata*	adult	Lodz	Grzegorz Tonczyk	Poland; 54.613, 18.51
MW490461	PLSW085-20	ODOPL_219	BOLD:AEC6167	658[0n]	30-Jun-2019	*Somatochlora flavomaculata*	adult	Lodz	Grzegorz Tonczyk	Poland; 54.613, 18.51
MT298621	ZPLOD720-20	MIB:ZPL:08520	BOLD:ABW6681	658[0n]		*Somatochlora meridionalis*	adult	Milano Bicocca	Giacomo Assandri	Italy; 41.5, 13.3
MT298623	ZPLOD722-20	MIB:ZPL:08522	BOLD:ABW6681	658[0n]		*Somatochlora meridionalis*	adult	Milano Bicocca	Giacomo Assandri	Italy; 42.9, 12.2
MT298622	ZPLOD721-20	MIB:ZPL:08521	BOLD:ABW6681	658[0n]		*Somatochlora meridionalis*	adult	Milano Bicocca	Giacomo Assandri	Italy; 43.3, 13.5
MT298626	ZPLOD725-20	MIB:ZPL:08525	BOLD:ABW6681	658[0n]		*Somatochlora meridionalis*	adult	Giacomo Assandri	Giacomo Assandri	Italy; 44.4, 8.1
MT298627	ZPLOD726-20	MIB:ZPL:08526	BOLD:ABW6681	658[0n]		*Somatochlora meridionalis*	adult	Giacomo Assandri	Giacomo Assandri	Italy; 44.4, 8.1
MT298628	ZPLOD727-20	MIB:ZPL:08527	BOLD:ABW6681	658[0n]		*Somatochlora meridionalis*	adult	Milano Bicocca	Giacomo Assandri	Italy; 44.4, 8.1
MT298624	ZPLOD723-20	MIB:ZPL:08523	BOLD:ABW6681	658[0n]		*Somatochlora meridionalis*	adult	Milano Bicocca	Giacomo Assandri	Italy; 46, 13.6
MT298625	ZPLOD724-20	MIB:ZPL:08524	BOLD:ABW6681	658[0n]		*Somatochlora meridionalis*	adult	Milano Bicocca	Giacomo Assandri	Italy; 46.2, 13.3
MT298620	ZPLOD837-20	MIB:ZPL:08637	BOLD:ABW6681	658[0n]		*Somatochlora meridionalis*	adult	Giacomo Assandri	Giacomo Assandri	Montenegro; 42.2, 19.1
MW490094	GBODO071-18	GBOL 20194	BOLD:ABW6681	318[0n]	12-Jul-2018	*Somatochlora metallica*	adult	SNSB-ZSM	Stefan Koch	Germany; 47.6668, 11.0506
MW490335	HETFI055-11	FinHet55	BOLD:ABW6681	658[0n]	30-Jul-2010	*Somatochlora metallica*	adult	Bergen	Steffen Roth	Norway; 69.2133, 29.1536
MW490093	HETFI056-11	FinHet56	BOLD:ABW6681	658[0n]	31-Jul-2010	*Somatochlora metallica*	adult	Bergen	Steffen Roth	Norway; 69.231, 29.1609
MW490197	HETFI053-11	FinHet53	BOLD:ABW6681	658[0n]	26-Jul-2010	*Somatochlora metallica*	adult	Bergen	Steffen Roth	Norway; 70.1322, 24.7669
AB708931	GBMIN24739-13	AB708931	BOLD:ABW6681	451[0n]	31-Jul-2001	*Somatochlora metallica*		GenBank, NCBI	0	Sweden; ,
MT298633	ZPLOD733-20	MIB:ZPL:08533	BOLD:ADC2840	658[0n]		*Stylurus flavipes*	adult	Giacomo Assandri	Giacomo Assandri	Italy; 45.3, 9
MW490267	FBAQU1426-13	GBOL00270	BOLD:AAK1032	658[0n]	02-Aug-2011	*Sympecma fusca*	adult	SNSB-ZSM	Stefan Koch	Germany; 47.7952, 11.3061
MW490497	GBODO158-18	GBOL 20281	BOLD:AAK1032	658[0n]	14-Apr-2018	*Sympecma fusca*	adult	SNSB-ZSM	Stefan Koch	Germany; 48.0616, 10.6428
MW490474	GBODO159-18	GBOL 20282	BOLD:AAK1032	658[0n]	12-May-2018	*Sympecma fusca*	adult	SNSB-ZSM	Stefan Koch	Germany; 48.0616, 10.6428
MW490235	GBODO162-18	GBOL 20285	BOLD:AAK1032	658[0n]	12-May-2018	*Sympecma fusca*	adult	SNSB-ZSM	Stefan Koch	Germany; 48.0616, 10.6428
MW490101	GBODO163-18	GBOL 20286	BOLD:AAK1032	450[4n]	12-May-2018	*Sympecma fusca*	adult	SNSB-ZSM	Stefan Koch	Germany; 48.0616, 10.6428
MW490264	GBODO164-18	GBOL 20287	BOLD:AAK1032	658[0n]	12-May-2018	*Sympecma fusca*	adult	SNSB-ZSM	Stefan Koch	Germany; 48.0616, 10.6428
MW490128	GBODO185-18	GBOL 20308	BOLD:AAK1032	658[0n]	14-Apr-2018	*Sympecma fusca*	adult	SNSB-ZSM	Stefan Koch	Germany; 48.0616, 10.6428
HM901877	FBAQU515-10	BC ZSM AQU 00420	BOLD:AAK1032	658[0n]	21-May-2009	*Sympecma fusca*	adult	SNSB-ZSM	S. V. Ober	Germany; 48.107, 11.458
HM422052	FBAQU322-09	BC ZSM AQU 00322	BOLD:AAK1032	658[0n]	23-May-2009	*Sympecma fusca*	adult	SNSB-ZSM	M. Schoen	Germany; 48.338, 11.803
MW490387	GBEPT935-14	GBOL05505	BOLD:AAK1032	658[0n]	22-May-2014	*Sympecma fusca*	adult	SNSB-ZSM	Michael Franzen	Germany; 48.378, 11.841
MW490457	GBEPT936-14	GBOL05506	BOLD:AAK1032	658[0n]	22-May-2014	*Sympecma fusca*	adult	SNSB-ZSM	Michael Franzen	Germany; 48.378, 11.841
MW490162	FBAQU1444-13	GBOL00288	BOLD:ACG0335	655[0n]	20-May-2012	*Sympecma paedisca*	adult	SNSB-ZSM	Stefan Koch	Germany; 47.6328, 10.5381
MW490560	GBEPT930-14	GBOL05500	BOLD:ACG0335	658[0n]	01-Jun-2014	*Sympecma paedisca*	adult	SNSB-ZSM	Michael Franzen	Germany; 47.709, 10.852
MW490423	GBEPT931-14	GBOL05501	BOLD:ACG0335	658[0n]	01-Jun-2014	*Sympecma paedisca*	adult	SNSB-ZSM	Michael Franzen	Germany; 47.709, 10.852
MW490366	GBEPT932-14	GBOL05502	BOLD:ACG0335	658[0n]	01-Jun-2014	*Sympecma paedisca*	adult	SNSB-ZSM	Michael Franzen	Germany; 47.709, 10.852
MW490220	GBEPT933-14	GBOL05503	BOLD:ACG0335	658[0n]	01-Jun-2014	*Sympecma paedisca*	adult	SNSB-ZSM	Michael Franzen	Germany; 47.709, 10.852
MW490346	GBEPT934-14	GBOL05504	BOLD:ACG0335	658[0n]	01-Jun-2014	*Sympecma paedisca*	adult	SNSB-ZSM	Michael Franzen	Germany; 47.709, 10.852
MW490223	PLSW173-20	ODOPL_238	BOLD:ACG0335	658[0n]	11-Jul-2019	*Sympecma paedisca*	larvae	Lodz	Grzegorz Tonczyk	Poland; 54.042, 23.057
MW490421	PLSW174-20	ODOPL_239	BOLD:ACG0335	653[0n]	11-Jul-2019	*Sympecma paedisca*	larvae	Lodz	Grzegorz Tonczyk	Poland; 54.042, 23.057
MW490470	PLSW175-20	ODOPL_240	BOLD:ACG0335	620[2n]	11-Jul-2019	*Sympecma paedisca*	larvae	Lodz	Grzegorz Tonczyk	Poland; 54.042, 23.057
MW490471	GBODO016-18	GBOL 20139	BOLD:AAA3766	318[0n]	27-Jul-2018	*Sympetrum danae*	adult	SNSB-ZSM	Stefan Koch	Germany; 47.8252, 11.3012
HM901878	FBAQU516-10	BC ZSM AQU 00421	BOLD:AAA3766	643[0n]	16-Jul-2009	*Sympetrum danae*	adult	SNSB-ZSM	S. V. Ober	Germany; 47.909, 11.522

GenBank #	Process ID	Sample ID	BIN	Seq. Length	Collection Date	Identification	Life Stage	Institution (short)	Identifier	country; GPS
MW490244	GBODO014-18	GBOL 20137	BOLD:AAA3766	318[1n]	27-Jul-2018	*Sympetrum danae*	adult	SNSB-ZSM	Stefan Koch	Germany; 47.9229, 11.5085
MW490334	PLSW001-20	ODOPL_135	BOLD:AAA3766	658[0n]	11-Jul-2019	*Sympetrum danae*	adult	Lodz	Grzegorz Tonczyk	Poland; 54.042, 23.063
MW490119	PLSW002-20	ODOPL_136	BOLD:AAA3766	658[0n]	11-Jul-2019	*Sympetrum danae*	adult	Lodz	Grzegorz Tonczyk	Poland; 54.042, 23.063
MW490531	PLSW003-20	ODOPL_137	BOLD:AAA3766	653[0n]	11-Jul-2019	*Sympetrum danae*	adult	Lodz	Grzegorz Tonczyk	Poland; 54.042, 23.063
MT298645	ZPLOD749-20	MIB:ZPL:08549	BOLD:ACQ1493	658[0n]		*Sympetrum depressiusculum*	adult	Milano Bicocca	Giacomo Assandri	Italy; 45.1, 7.2
MT298644	ZPLOD752-20	MIB:ZPL:08552	BOLD:ACQ1493	658[0n]		*Sympetrum depressiusculum*	adult	Giacomo Assandri	Giacomo Assandri	Italy; 45.7, 9.4
MT298647	ZPLOD758-20	MIB:ZPL:08558	BOLD:ADI4820	658[0n]		*Sympetrum flaveolum*	adult	Milano Bicocca	Giacomo Assandri	Italy; 42.4, 13.7
MT298646	ZPLOD757-20	MIB:ZPL:08557	BOLD:ADI4820	658[0n]		*Sympetrum flaveolum*	adult	Milano Bicocca	Giacomo Assandri	Italy; 44.6, 9.5
MT298649	ZPLOD753-20	MIB:ZPL:08553	BOLD:ADI4820	658[0n]		*Sympetrum flaveolum*	adult	Milano Bicocca	Giacomo Assandri	Italy; 45.8, 7.6
HM901901	FBAQU552-10	BC ZSM AQU 00457	BOLD:AAI0218	626[0n]	19-Sep-2009	*Sympetrum fonscolombii*	adult	SNSB-ZSM	K. Burbach	Germany; 47.808, 11.458
HM901879	FBAQU517-10	BC ZSM AQU 00422	BOLD:AAI0218	606[0n]	25-May-2009	*Sympetrum fonscolombii*	adult	SNSB-ZSM	S. V. Ober	Germany; 48.107, 11.458
MW490426	PLSW086-20	ODOPL_220	BOLD:AAI0218	658[0n]	01-Jul-2019	*Sympetrum fonscolombii*	adult	Lodz	Grzegorz Tonczyk	Poland; 54.788, 18.427
MW490444	PLSW087-20	ODOPL_221	BOLD:AAI0218	658[0n]	01-Jul-2019	*Sympetrum fonscolombii*	adult	Lodz	Grzegorz Tonczyk	Poland; 54.788, 18.427
MW490374	PLSW088-20	ODOPL_222	BOLD:AAI0218	658[0n]	01-Jul-2019	*Sympetrum fonscolombii*	adult	Lodz	Grzegorz Tonczyk	Poland; 54.788, 18.427
MW490559	GBEPT950-14	GBOL05520	BOLD:AAK1015	651[0n]	14-Jun-2014	*Sympetrum meridionale*	adult	SNSB-ZSM	Michael Franzen	Austria; 48.132, 16.95
HM901902	FBAQU553-10	BC ZSM AQU 00458	BOLD:AAK1022	586[1n]	09-Jan-2009	*Sympetrum pedemontanum*	adult	SNSB-ZSM	F. Weihrauch	Germany; 47.895, 12.167
MW490257	FBAQU323-09	BC ZSM AQU 00323	BOLD:AAK1022	487[0n]	15-Aug-2008	*Sympetrum pedemontanum*	adult	SNSB-ZSM	M. Schoen	Germany; 48.339, 11.799
MW490478	FBAQU554-10	BC ZSM AQU 00459	BOLD:AAB2237	658[2n]	09-Jan-2009	*Sympetrum sanguineum*	adult	SNSB-ZSM	F. Weihrauch	Germany; 47.895, 12.167
HM901880	FBAQU518-10	BC ZSM AQU 00423	BOLD:AAB2237	631[0n]	16-Jul-2009	*Sympetrum sanguineum*	adult	SNSB-ZSM	S. V. Ober	Germany; 47.908, 11.523
MW490210	FBAQU324-09	BC ZSM AQU 00324	BOLD:AAB2237	428[0n]	16-Jul-2009	*Sympetrum sanguineum*	adult	SNSB-ZSM	M. Schoen	Germany; 48.314, 11.818
MW490516	PLSW066-20	ODOPL_200	BOLD:AAB2237	658[0n]	25-Aug-2019	*Sympetrum sanguineum*	adult	Lodz	Grzegorz Tonczyk	Poland; 51.757, 19.4
MW490476	PLSW067-20	ODOPL_201	BOLD:AAB2237	632[0n]	25-Aug-2019	*Sympetrum sanguineum*	adult	Lodz	Grzegorz Tonczyk	Poland; 51.757, 19.4
MW490150	PLSW068-20	ODOPL_202	BOLD:AAB2237	658[0n]	25-Aug-2019	*Sympetrum sanguineum*	adult	Lodz	Grzegorz Tonczyk	Poland; 51.757, 19.4
MW490284	GBMIX3758-18	GBOL19826	BOLD:AAB2236	312[1n]	24-Sep-2017	*Sympetrum striolatum*	adult	SNSB-ZSM	Lars Hendrich	Germany; 48.0342, 10.3834
HM901881	FBAQU519-10	BC ZSM AQU 00424	BOLD:AAB2236	641[0n]	09-Aug-2009	*Sympetrum striolatum*	adult	SNSB-ZSM	S. V. Ober	Germany; 48.073, 11.541
GU682171	FBAQU325-09	BC ZSM AQU 00325	BOLD:AAB2236	658[0n]	02-Aug-2009	*Sympetrum striolatum*	adult	SNSB-ZSM	M. Schoen	Germany; 48.34, 11.807
MW490209	ZMBN328-16	ZMBN-Ent13	BOLD:AAB2236	658[0n]	23-Jun-2016	*Sympetrum striolatum*		Bergen	Steffen Roth	Norway; 59.7137, 5.2176
MW490551	TRDOD085-14	TRD-ODO103	BOLD:AAB2236	429[0n]	30-Jul-2004	*Sympetrum striolatum*	adult	NTNU	Dag Dolmen	Norway; 65.092, 11.911
MW490084	TRDOD086-14	TRD-ODO104	BOLD:AAB2236	658[5n]	30-Jul-2004	*Sympetrum striolatum*	adult	NTNU	Dag Dolmen	Norway; 65.092, 11.911
HM901903	FBAQU555-10	BC ZSM AQU 00460	BOLD:AAE2658	599[0n]	16-Jul-2009	*Sympetrum vulgatum*	adult	SNSB-ZSM	F. Weihrauch	Germany; 47.895, 12.167
MW490543	GBODO113-18	GBOL 20236	BOLD:AAE2658	658[0n]	19-Jul-2018	*Sympetrum vulgatum*	adult	SNSB-ZSM	Stefan Koch	Germany; 47.9229, 11.5085
HM901882	FBAQU520-10	BC ZSM AQU 00425	BOLD:AAE2658	658[0n]	15-Aug-2009	*Sympetrum vulgatum*	adult	SNSB-ZSM	S. V. Ober	Germany; 48.2, 11.485
GU682170	FBAQU326-09	BC ZSM AQU 00326	BOLD:AAE2658	658[0n]	16-Jul-2009	*Sympetrum vulgatum*	adult	SNSB-ZSM	M. Schoen	Germany; 48.298, 11.822
MW490266	PLSW081-20	ODOPL_215	BOLD:AAE2658	658[0n]	07-Aug-2019	*Sympetrum vulgatum*	adult	Lodz	Grzegorz Tonczyk	Poland; 49.429, 21.859
MW490405	PLSW082-20	ODOPL_216	BOLD:AAE2658	658[0n]	07-Aug-2019	*Sympetrum vulgatum*	adult	Lodz	Grzegorz Tonczyk	Poland; 49.429, 21.859
MW490462	PLSW083-20	ODOPL_217	BOLD:AAE2658	658[0n]	07-Aug-2019	*Sympetrum vulgatum*	adult	Lodz	Grzegorz Tonczyk	Poland; 49.429, 21.859
MT298674	ZPLOD811-20	MIB:ZPL:08611	BOLD:ABA9471	658[0n]		*Trithemis annulata*	adult	Milano Bicocca	Giacomo Assandri	Italy; 37.7, 12.8
MT298673	ZPLOD809-20	MIB:ZPL:08609	BOLD:ABA9471	658[0n]		*Trithemis annulata*	adult	Giacomo Assandri	Giacomo Assandri	Italy; 45.4, 9.1
MT298677	ZPLOD848-20	MIB:ZPL:08648	BOLD:ABA9387	658[0n]		*Trithemis kirbyi*	adult	Milano Bicocca	Giacomo Assandri	Morocco; 28.5, -10
MT298678	ZPLOD847-20	MIB:ZPL:08647	BOLD:ABA9387	658[0n]		*Trithemis kirbyi*	adult	Giacomo Assandri	Giacomo Assandri	Morocco; 29.1, -10.3
MT298679	ZPLOD812-20	MIB:ZPL:08612	BOLD:AAZ4163	658[0n]		*Zygonyx torridus*	adult	Milano Bicocca	Giacomo Assandri	Italy; 37.7, 12.9
