# Supplementary material for: Coverage and quality of DNA barcode references for Central and Northern European Odonata
Source: PeerJ. 2021 May 3;9:e11192. doi: 10.7717/peerj.11192 (PMC8101477; doi:10.7717/peerj.11192)
Supplement: Supplemental Information 2 [file peerj-09-11192-s002.pdf]

Family	Species	# individuals	countries	Mean Intra-Sp	Max Intra-Sp	Nearest Species	Nearest Neighbour	Distance to NN
Aeshnidae	*Aeshna affinis*	5	3	0.18	0.31	*Aeshna mixta*	FBAQU480-10	7.49
Aeshnidae	*Aeshna caerulea*	5	2	0.15	0.32	*Aeshna subarctica*	GBODO045-18	5.88
Aeshnidae	*Aeshna cyanea*	7	3	0.26	0.47	*Aeshna mixta*	GODO046-19	6.22
Aeshnidae	*Aeshna grandis*	9	3	0.19	0.33	*Aeshna viridis*	GBMIN88539-17	2.08
Aeshnidae	*Aeshna isoceles*	5	3	0.44	1.1	*Aeshna caerulea*	TRDOD067-14	8.26
Aeshnidae	*Aeshna juncea*	9	2	0.38	0.93	*Aeshna subarctica*	TRDOD062-14	3.01
Aeshnidae	*Aeshna mixta*	4	2	0.28	0.48	*Aeshna subarctica*	GBMIN88537-17	6.1
Aeshnidae	*Aeshna subarctica*	9	2	0.2	0.5	*Aeshna juncea*	GBODO009-18	3.01
Aeshnidae	*Aeshna viridis*	2	1	0.34	0.34	*Aeshna grandis*	ODOPL066-19	2.08
Aeshnidae	*Anax ephippiger*	2	1	1.24	1.24	*Anax imperator*	FBAQU481-10	6.35
Aeshnidae	*Anax imperator*	12	2	0.35	0.93	*Anax parthenope*	ODOPL134-19	0.55
Aeshnidae	*Anax parthenope*	6	3	0.56	0.77	*Anax imperator*	FBAQU481-10	0.55
Aeshnidae	*Boyeria irene*	1	1	N/A	0	*Aeshna subarctica*	GBMIN88537-17	8.1
Aeshnidae	*Brachytron pratense*	8	3	0.16	0.66	*Caliaeschna microstigma*	ZPLOD815-20	7.9
Aeshnidae	*Caliaeschna microstigma*	1	1	N/A	0	*Aeshna mixta*	GODO046-19	7.39
Calopterygidae	*Calopteryx haemorrhoidalis*	1	1	N/A	0	*Calopteryx splendens*	ODOPL034-19	15.37
Calopterygidae	*Calopteryx splendens*	16	2	0.5	1.31	*Calopteryx xanthostoma*	ZPLOD124-20	0
Calopterygidae	*Calopteryx virgo*	4	2	0	0	*Calopteryx splendens*	GODO025-18	9.33
Calopterygidae	*Calopteryx xanthostoma*	2	1	0	0	*Calopteryx splendens*	GODO022-18	0
Coenagrionidae	*Ceriagrion tenellum*	7	1	0.18	0.31	*Pyrrhosoma nymphula*	ODTRI003-14	16.22
Coenagrionidae	*Coenagrion armatum*	4	1	0	0	*Coenagrion hastulatum*	PLSW010-20	9.28
Coenagrionidae	*Coenagrion caerulescens*	8	1	1.44	2.34	*Coenagrion scitulum*	GBEPT907-14	9.21
Coenagrionidae	*Coenagrion hastulatum*	9	3	0.17	0.68	*Coenagrion armatum*	TRDOD019-14	9.28
Coenagrionidae	*Coenagrion johanssoni*	5	1	0	0	*Coenagrion armatum*	TRDOD019-14	10.36
Coenagrionidae	*Coenagrion mercuriale*	2	1	0	0	*Coenagrion pulchellum*	ODTRI008-14	9.38
Coenagrionidae	*Coenagrion ornatum*	5	1	0.06	0.15	*Coenagrion puella*	ODOPL104-19	0
Coenagrionidae	*Coenagrion puella*	26	2	0.08	0.49	*Coenagrion ornatum*	FBAQU310-09	0
Coenagrionidae	*Coenagrion pulchellum*	27	3	0.65	2.79	*Coenagrion puella*	ODOPL104-19	0
Coenagrionidae	*Coenagrion scitulum*	4	2	0.08	0.15	*Coenagrion caerulescens*	ZPLOD197-20	9.21
Coenagrionidae	*Enallagma cyathigerum*	36	3	0.21	1.17	*Ischnura elegans*	ODOPL087-19	13.86
Coenagrionidae	*Erythromma lindenii*	2	1	0	0	*Erythromma najas*	FBAQU492-10	16.04
Coenagrionidae	*Erythromma najas*	14	3	0.21	0.66	*Erythromma viridulum*	GBMIX949-14	15.06
Coenagrionidae	*Erythromma viridulum*	8	2	0.46	1.87	*Erythromma najas*	FBAQU563-10	15.06
Coenagrionidae	*Ischnura elegans*	49	5	0.18	1.83	*Ischnura saharensis*	ZPLOD844-20	0
Coenagrionidae	*Ischnura genei*	3	1	0.2	0.31	*Ischnura elegans*	TRDOD090-14	0
Coenagrionidae	*Ischnura pumilio*	4	1	0	0	*Ischnura elegans*	TRDOD090-14	14.35
Coenagrionidae	*Ischnura saharensis*	2	1	0.31	0.31	*Ischnura elegans*	ODOPL088-19	0
Coenagrionidae	*Nehalennia speciosa*	9	2	0	0	*Pyrrhosoma nymphula*	FBAQU510-10	15.72
Coenagrionidae	*Pyrrhosoma nymphula*	19	2	0.16	0.66	*Leucorrhinia albifrons*	EDF004-18	14.65
Cordulegastridae	*Cordulegaster bidentata*	1	1	N/A	0	*Cordulegaster trinacriae*	ZPLOD315-20	8.31
Cordulegastridae	*Cordulegaster boltonii*	8	3	0	0	*Cordulegaster trinacriae*	ZPLOD314-20	5.37
Cordulegastridae	*Cordulegaster heros*	3	2	0.15	0.15	*Cordulegaster picta*	GBMIN88645-17	6.67
Cordulegastridae	*Cordulegaster picta*	1	1	N/A	0	*Cordulegaster heros*	EDF003-18	6.67
Cordulegastridae	*Cordulegaster trinacriae*	2	1	0.15	0.15	*Cordulegaster boltonii*	FBAQU531-10	5.37
Corduliidae	*Cordulia aenea*	16	4	2.4	4.69	*Somatochlora alpestris*	ZPLOD706-20	10.73
Corduliidae	*Epitheca bimaculata*	1	1	N/A	0	*Somatochlora alpestris*	ZPLOD706-20	12.7
Corduliidae	*Somatochlora alpestris*	4	1	0.08	0.15	*Somatochlora arctica*	TRDOD064-14	4.27
Corduliidae	*Somatochlora arctica*	4	1	0.24	0.49	*Somatochlora alpestris*	ZPLOD706-20	4.27
Corduliidae	*Somatochlora flavomaculata*	9	2	0.44	0.99	*Somatochlora arctica*	TRDOD064-14	7.48
Corduliidae	*Somatochlora meridionalis*	9	2	0.52	0.92	*Somatochlora metallica*	HETFI055-11	0.31
Corduliidae	*Somatochlora metallica*	5	3	0	0	*Somatochlora meridionalis*	ZPLOD723-20	0.31
Gomphidae	*Gomphus pulchellus*	1	1	N/A	0	*Ophiogomphus cecilia*	ODOPL039-19	13.97
Gomphidae	*Gomphus schneiderii*	2	1	0.15	0.15	*Gomphus vulgatissimus*	FBAQU1445-13	0.72
Gomphidae	*Gomphus simillimus*	1	1	N/A	0	*Gomphus vulgatissimus*	GBMIX410-14	6.62
Gomphidae	*Gomphus vulgatissimus*	13	2	0.22	0.79	*Gomphus schneiderii*	ZPLOD824-20	0.72
Gomphidae	*Lindenia tetraphylla*	5	1	0.09	0.15	*Paragomphus genei*	ZPLOD676-20	16.16
Gomphidae	*Onychogomphus forcipatus*	24	4	1.44	2.97	*Caliaeschna microstigma*	ZPLOD815-20	11.01
Gomphidae	*Onychogomphus uncatus*	4	1	0.08	0.15	*Ophiogomphus cecilia*	GBMIX418-14	12.52
Gomphidae	*Ophiogomphus cecilia*	10	3	0.36	0.98	*Onychogomphus uncatus*	ZPLOD581-20	12.52
Gomphidae	*Paragomphus genei*	3	1	0.31	0.46	*Anax parthenope*	ODOPL134-19	14.72
Gomphidae	*Stylurus flavipes*	1	1	N/A	0	*Onychogomphus forcipatus*	ZPLOD562-20	15.99
Lestidae	*Chalcolestes parvidens*	5	2	0.06	0.15	*Chalcolestes viridis*	ZPLOD167-20	8.92
Lestidae	*Chalcolestes viridis*	12	2	0.25	0.77	*Chalcolestes parvidens*	ZPLOD143-20	8.92
Lestidae	*Lestes barbarus*	5	3	0	0	*Lestes sponsa*	PLSW061-20	12.04
Lestidae	*Lestes dryas*	3	1	0.05	0.15	*Lestes sponsa*	PLSW061-20	4.93
Lestidae	*Lestes macrostigma*	2	1	0.46	0.46	*Lestes sponsa*	TRDOD030-14	14.04
Lestidae	*Lestes sponsa*	10	3	0.19	0.33	*Lestes dryas*	PLSW057-20	4.93
Lestidae	*Lestes virens*	11	2	0.3	0.65	*Lestes dryas*	PLSW056-20	12.41

Family	Species	# individuals	countries	Mean Intra-Sp	Max Intra-Sp	Nearest Species	Nearest Neighbour	Distance to NN
Lestidae	*Sympecma fusca*	11	1	0.25	1.08	*Sympecma paedisca*	GBEPT934-14	8.14
Lestidae	*Sympecma paedisca*	9	2	0.64	1.09	*Sympecma fusca*	GBODO158-18	8.14
Libellulidae	*Brachythemis impartita*	3	1	0	0	*Leucorrhinia albifrons*	EDF004-18	14.09
Libellulidae	*Crocothemis erythraea*	6	3	0.56	0.96	*Leucorrhinia rubicunda*	TRDOD013-14	13.8
Libellulidae	*Diplacodes lefebvrii*	2	1	0.46	0.46	*Libellula quadrimaculata*	FBAQU503-10	17.13
Libellulidae	*Leucorrhinia albifrons*	3	2	0	0	*Leucorrhinia caudalis*	EDF011-18	10.07
Libellulidae	*Leucorrhinia caudalis*	2	1	0	0	*Leucorrhinia albifrons*	EDF004-18	10.07
Libellulidae	*Leucorrhinia dubia*	12	3	0.17	0.65	*Leucorrhinia rubicunda*	TRDOD013-14	2.23
Libellulidae	*Leucorrhinia pectoralis*	6	3	0	0	*Leucorrhinia dubia*	FBAQU501-10	4.72
Libellulidae	*Leucorrhinia rubicunda*	1	1	N/A	0	*Leucorrhinia dubia*	FBAQU501-10	2.23
Libellulidae	*Libellula depressa*	6	3	0	0	*Libellula fulva*	GBEPT944-14	12.84
Libellulidae	*Libellula fulva*	5	3	0.08	0.15	*Libellula depressa*	RODI027-20	12.84
Libellulidae	*Libellula quadrimaculata*	17	3	0.3	0.81	*Orthetrum coerulescens*	PLSW049-20	12.47
Libellulidae	*Orthetrum albistylum*	1	1	N/A	0	*Orthetrum cancellatum*	PLSW029-20	5.92
Libellulidae	*Orthetrum brunneum*	5	2	0	0	*Orthetrum nitidinerve*	ZPLOD661-20	9.44
Libellulidae	*Orthetrum cancellatum*	9	2	0.78	1.55	*Orthetrum albistylum*	EDF010-18	5.92
Libellulidae	*Orthetrum chrysostigma*	1	1	N/A	0	*Orthetrum albistylum*	EDF010-18	8.67
Libellulidae	*Orthetrum coerulescens*	10	3	0.35	1.39	*Orthetrum brunneum*	GBODO060-18	9.87
Libellulidae	*Orthetrum nitidinerve*	2	1	0	0	*Orthetrum brunneum*	GBODO060-18	9.44
Libellulidae	*Orthetrum trinacria*	3	1	0.93	1.39	*Orthetrum cancellatum*	PLSW029-20	12.11
Libellulidae	*Selysiothemis nigra*	3	1	0.31	0.46	*Trithemis kirbyi*	ZPLOD848-20	14.49
Libellulidae	*Sympetrum danae*	6	2	0	0	*Sympetrum depressiusculum*	ZPLOD752-20	9.88
Libellulidae	*Sympetrum depressiusculum*	2	1	0	0	*Sympetrum danae*	PLSW002-20	9.88
Libellulidae	*Sympetrum flaveolum*	3	1	0.51	0.77	*Sympetrum sanguineum*	PLSW068-20	12.76
Libellulidae	*Sympetrum fonscolombii*	5	2	0.26	0.48	*Sympetrum depressiusculum*	ZPLOD752-20	14.47
Libellulidae	*Sympetrum meridionale*	1	1	N/A	0	*Sympetrum sanguineum*	FBAQU518-10	9.65
Libellulidae	*Sympetrum pedemontanum*	2	1	N/A	0	*Sympetrum vulgatum*	GBODO113-18	10.61
Libellulidae	*Sympetrum sanguineum*	6	2	0.44	0.8	*Sympetrum meridionale*	GBEPT950-14	9.65
Libellulidae	*Sympetrum striolatum*	6	2	0.23	0.46	*Sympetrum vulgatum*	GBODO113-18	9.63
Libellulidae	*Sympetrum vulgatum*	7	2	0.09	0.31	*Sympetrum striolatum*	ZMBN328-16	9.63
Libellulidae	*Trithemis annulata*	2	1	0.15	0.15	*Trithemis kirbyi*	ZPLOD848-20	11.56
Libellulidae	*Trithemis kirbyi*	2	1	0.62	0.62	*Trithemis annulata*	ZPLOD811-20	11.56
Libellulidae	*Zygonyx torridus*	1	1	N/A	0	*Libellula quadrimaculata*	FBAQU503-10	17.59
Platycnemididae	*Platycnemis pennipes*	10	3	0	0	*Erythromma najas*	FBAQU492-10	17.9
Synthemistidae	*Oxygastra curtisii*	1	1	N/A	0	*Orthetrum cancellatum*	ODOPL128-19	14.71
