# Supplementary material for: Coverage and quality of DNA barcode references for Central and Northern European Odonata
Source: PeerJ. 2021 May 3;9:e11192. doi: 10.7717/peerj.11192 (PMC8101477; doi:10.7717/peerj.11192)
Supplement: Supplemental Information 3 — Cases of haplotype sharing are highlighted by red frames, details for tree reconstruction method and sample composition are given in the pdf tree file. [file peerj-09-11192-s003.pdf]

BOLD TaxonID Tree

Title	:	Tree Result - DS-ODOGER
Date	:	15-May-2020
Data Type	:	Nucleotide
Distance Model	:	Kimura 2 Parameter
Marker	:	COI-5P
Colourization	:	Barcode Cluster (BIN)
Label	:	Process ID
Label	:	Taxon
Label	:	Life Stage
Label	:	Country
Label	:	Province/State
Label	:	Sequence Length
Filter	:	length > 200bp only
Filter	:	exclude records flagged as misidentifications
Filter	:	exclude records with stop codons
Filter	:	exclude contaminants
Sequence Count	:	697
Species count	:	103
Genus count	:	38
Family count	:	10
Unidentified	:	2
BIN Count	:	96

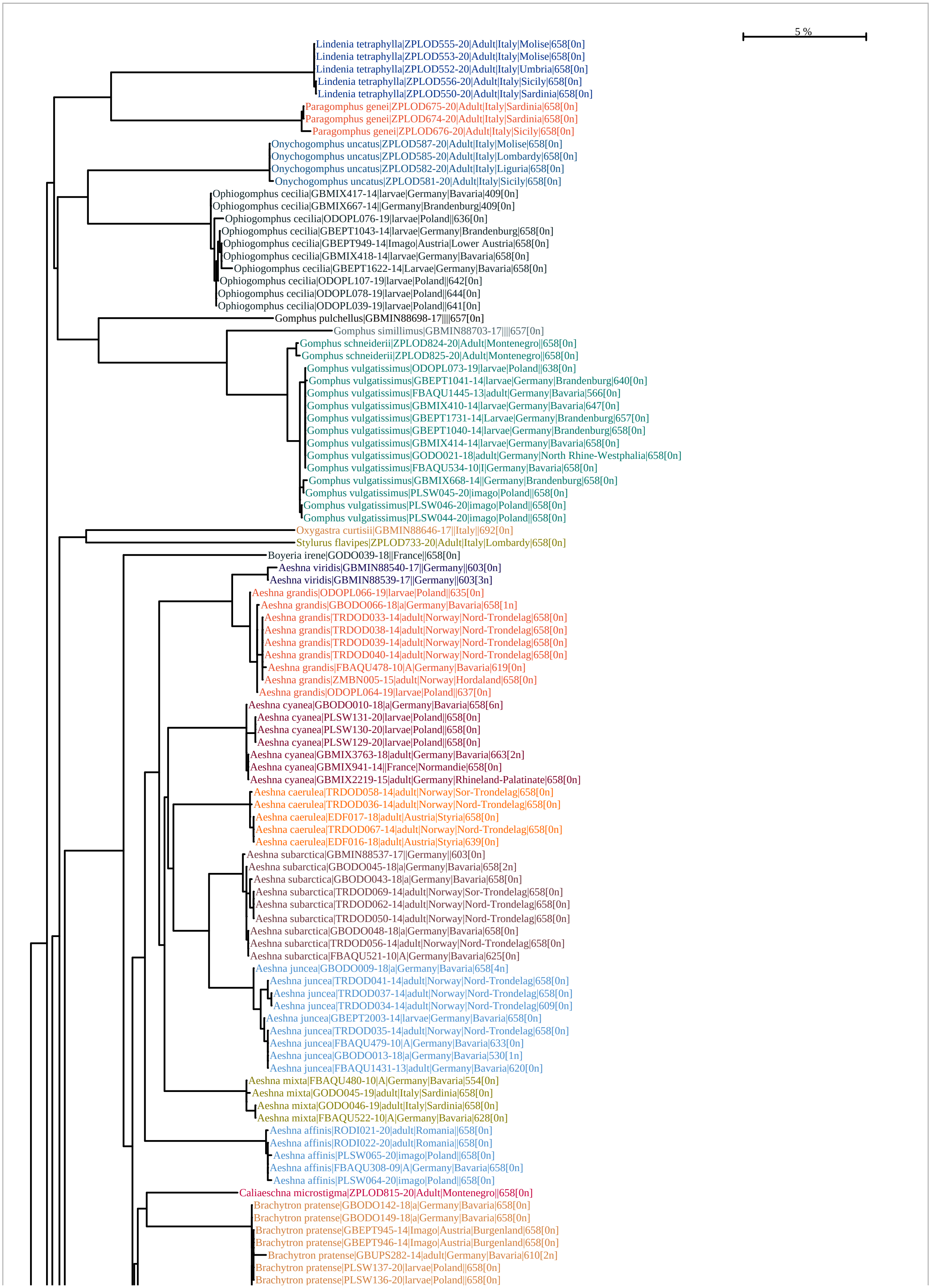
5 %
Lindenia tetraphylla|ZPLOD555-20|Adult|Italy|Molise|658[0n]
Lindenia tetraphylla|ZPLOD553-20|Adult|Italy|Molise|658[0n]
Lindenia tetraphylla|ZPLOD552-20|Adult|Italy|Umbria|658[0n]
Lindenia tetraphylla|ZPLOD556-20|Adult|Italy|Sicily|658[0n]
Lindenia tetraphylla|ZPLOD550-20|Adult|Italy|Sardinia|658[0n]
Paragomphus genei|ZPLOD675-20|Adult|Italy|Sardinia|658[0n]
Paragomphus genei|ZPLOD674-20|Adult|Italy|Sardinia|658[0n]
Paragomphus genei|ZPLOD676-20|Adult|Italy|Sicily|658[0n]
Onychogomphus uncatus|ZPLOD587-20|Adult|Italy|Molise|658[0n]
Onychogomphus uncatus|ZPLOD585-20|Adult|Italy|Lombardy|658[0n]
Onychogomphus uncatus|ZPLOD582-20|Adult|Italy|Liguria|658[0n]
Onychogomphus uncatus|ZPLOD581-20|Adult|Italy|Sicily|658[0n]
Ophiogomphus cecilia|GBMIX417-14|larvae|Germany|Bavaria|409[0n]
Ophiogomphus cecilia|GBMIX667-14||Germany|Brandenburg|409[0n]
Ophiogomphus cecilia|ODOPL076-19|larvae|Poland||636[0n]
Ophiogomphus cecilia|GBEPT1043-14|larvae|Germany|Brandenburg|658[0n]
Ophiogomphus cecilia|GBEPT949-14|Imago|Austria|Lower Austria|658[0n]
Ophiogomphus cecilia|GBMIX418-14|larvae|Germany|Bavaria|658[0n]
Ophiogomphus cecilia|GBEPT1622-14|Larvae|Germany|Bavaria|658[0n]
Ophiogomphus cecilia|ODOPL107-19|larvae|Poland||642[0n]
Ophiogomphus cecilia|ODOPL078-19|larvae|Poland||644[0n]
Ophiogomphus cecilia|ODOPL039-19|larvae|Poland||641[0n]
Gomphus pulchellus|GBMIN88698-17||||657[0n]
Gomphus simillimus|GBMIN88703-17||||657[0n]
Gomphus schneiderii|ZPLOD824-20|Adult|Montenegro||658[0n]
Gomphus schneiderii|ZPLOD825-20|Adult|Montenegro||658[0n]
Gomphus vulgatissimus|ODOPL073-19|larvae|Poland||638[0n]
Gomphus vulgatissimus|GBEPT1041-14|larvae|Germany|Brandenburg|640[0n]
Gomphus vulgatissimus|FBAQU1445-13|adult|Germany|Bavaria|566[0n]
Gomphus vulgatissimus|GBMIX410-14|larvae|Germany|Bavaria|647[0n]
Gomphus vulgatissimus|GBEPT1731-14|Larvae|Germany|Brandenburg|657[0n]
Gomphus vulgatissimus|GBEPT1040-14|larvae|Germany|Brandenburg|658[0n]
Gomphus vulgatissimus|GBMIX414-14|larvae|Germany|Bavaria|658[0n]
Gomphus vulgatissimus|GODO021-18|adult|Germany|North Rhine-Westphalia|658[0n]
Gomphus vulgatissimus|FBAQU534-10|I|Germany|Bavaria|658[0n]
Gomphus vulgatissimus|GBMIX668-14||Germany|Brandenburg|658[0n]
Gomphus vulgatissimus|PLSW045-20|imago|Poland||658[0n]
Gomphus vulgatissimus|PLSW046-20|imago|Poland||658[0n]
Gomphus vulgatissimus|PLSW044-20|imago|Poland||658[0n]
Oxygastra curtisii|GBMIN88646-17||Italy||692[0n]
Stylurus flavipes|ZPLOD733-20|Adult|Italy|Lombardy|658[0n]
Boyeria irene|GODO039-18||France||658[0n]
Aeshna viridis|GBMIN88540-17||Germany||603[0n]
Aeshna viridis|GBMIN88539-17||Germany||603[3n]
Aeshna grandis|ODOPL066-19|larvae|Poland||635[0n]
Aeshna grandis|GBODO066-18|a|Germany|Bavaria|658[1n]
Aeshna grandis|TRDOD033-14|adult|Norway|Nord-Trondelag|658[0n]
Aeshna grandis|TRDOD038-14|adult|Norway|Nord-Trondelag|658[0n]
Aeshna grandis|TRDOD039-14|adult|Norway|Nord-Trondelag|658[0n]
Aeshna grandis|TRDOD040-14|adult|Norway|Nord-Trondelag|658[0n]
Aeshna grandis|FBAQU478-10|A|Germany|Bavaria|619[0n]
Aeshna grandis|ZMBN005-15|adult|Norway|Hordaland|658[0n]
Aeshna grandis|ODOPL064-19|larvae|Poland||637[0n]
Aeshna cyanea|GBODO010-18|a|Germany|Bavaria|658[6n]
Aeshna cyanea|PLSW131-20|larvae|Poland||658[0n]
Aeshna cyanea|PLSW130-20|larvae|Poland||658[0n]
Aeshna cyanea|PLSW129-20|larvae|Poland||658[0n]
Aeshna cyanea|GBMIX3763-18|adult|Germany|Bavaria|663[2n]
Aeshna cyanea|GBMIX941-14||France|Normandie|658[0n]
Aeshna cyanea|GBMIX2219-15|adult|Germany|Rhineland-Palatinate|658[0n]
Aeshna caerulea|TRDOD058-14|adult|Norway|Sor-Trondelag|658[0n]
Aeshna caerulea|TRDOD036-14|adult|Norway|Nord-Trondelag|658[0n]
Aeshna caerulea|EDF017-18|adult|Austria|Styria|658[0n]
Aeshna caerulea|TRDOD067-14|adult|Norway|Nord-Trondelag|658[0n]
Aeshna caerulea|EDF016-18|adult|Austria|Styria|639[0n]
Aeshna subarctica|GBMIN88537-17||Germany||603[0n]
Aeshna subarctica|GBODO045-18|a|Germany|Bavaria|658[2n]
Aeshna subarctica|GBODO043-18|a|Germany|Bavaria|658[0n]
Aeshna subarctica|TRDOD069-14|adult|Norway|Sor-Trondelag|658[0n]
Aeshna subarctica|TRDOD062-14|adult|Norway|Nord-Trondelag|658[0n]
Aeshna subarctica|TRDOD050-14|adult|Norway|Nord-Trondelag|658[0n]
Aeshna subarctica|GBODO048-18|a|Germany|Bavaria|658[0n]
Aeshna subarctica|TRDOD056-14|adult|Norway|Nord-Trondelag|658[0n]
Aeshna subarctica|FBAQU521-10|A|Germany|Bavaria|625[0n]
Aeshna juncea|GBODO009-18|a|Germany|Bavaria|658[4n]
Aeshna juncea|TRDOD041-14|adult|Norway|Nord-Trondelag|658[0n]
Aeshna juncea|TRDOD037-14|adult|Norway|Nord-Trondelag|658[0n]
Aeshna juncea|TRDOD034-14|adult|Norway|Nord-Trondelag|609[0n]
Aeshna juncea|GBEPT2003-14|larvae|Germany|Bavaria|658[0n]
Aeshna juncea|TRDOD035-14|adult|Norway|Nord-Trondelag|658[0n]
Aeshna juncea|FBAQU479-10|A|Germany|Bavaria|633[0n]
Aeshna juncea|GBODO013-18|a|Germany|Bavaria|530[1n]
Aeshna juncea|FBAQU1431-13|adult|Germany|Bavaria|620[0n]
Aeshna mixta|FBAQU480-10|A|Germany|Bavaria|554[0n]
Aeshna mixta|GODO045-19|adult|Italy|Sardinia|658[0n]
Aeshna mixta|GODO046-19|adult|Italy|Sardinia|658[0n]
Aeshna mixta|FBAQU522-10|A|Germany|Bavaria|628[0n]
Aeshna affinis|RODI021-20|adult|Romania||658[0n]
Aeshna affinis|RODI022-20|adult|Romania||658[0n]
Aeshna affinis|PLSW065-20|imago|Poland||658[0n]
Aeshna affinis|FBAQU308-09|A|Germany|Bavaria|658[0n]
Aeshna affinis|PLSW064-20|imago|Poland||658[0n]
Caliaeschna microstigma|ZPLOD815-20|Adult|Montenegro||658[0n]
Brachytron pratense|GBODO142-18|a|Germany|Bavaria|658[0n]
Brachytron pratense|GBODO149-18|a|Germany|Bavaria|658[0n]
Brachytron pratense|GBEPT945-14|Imago|Austria|Burgenland|658[0n]
Brachytron pratense|GBEPT946-14|Imago|Austria|Burgenland|658[0n]
Brachytron pratense|GBUPS282-14|adult|Germany|Bavaria|610[2n]
Brachytron pratense|PLSW137-20|larvae|Poland||658[0n]
Brachytron pratense|PLSW136-20|larvae|Poland||658[0n]

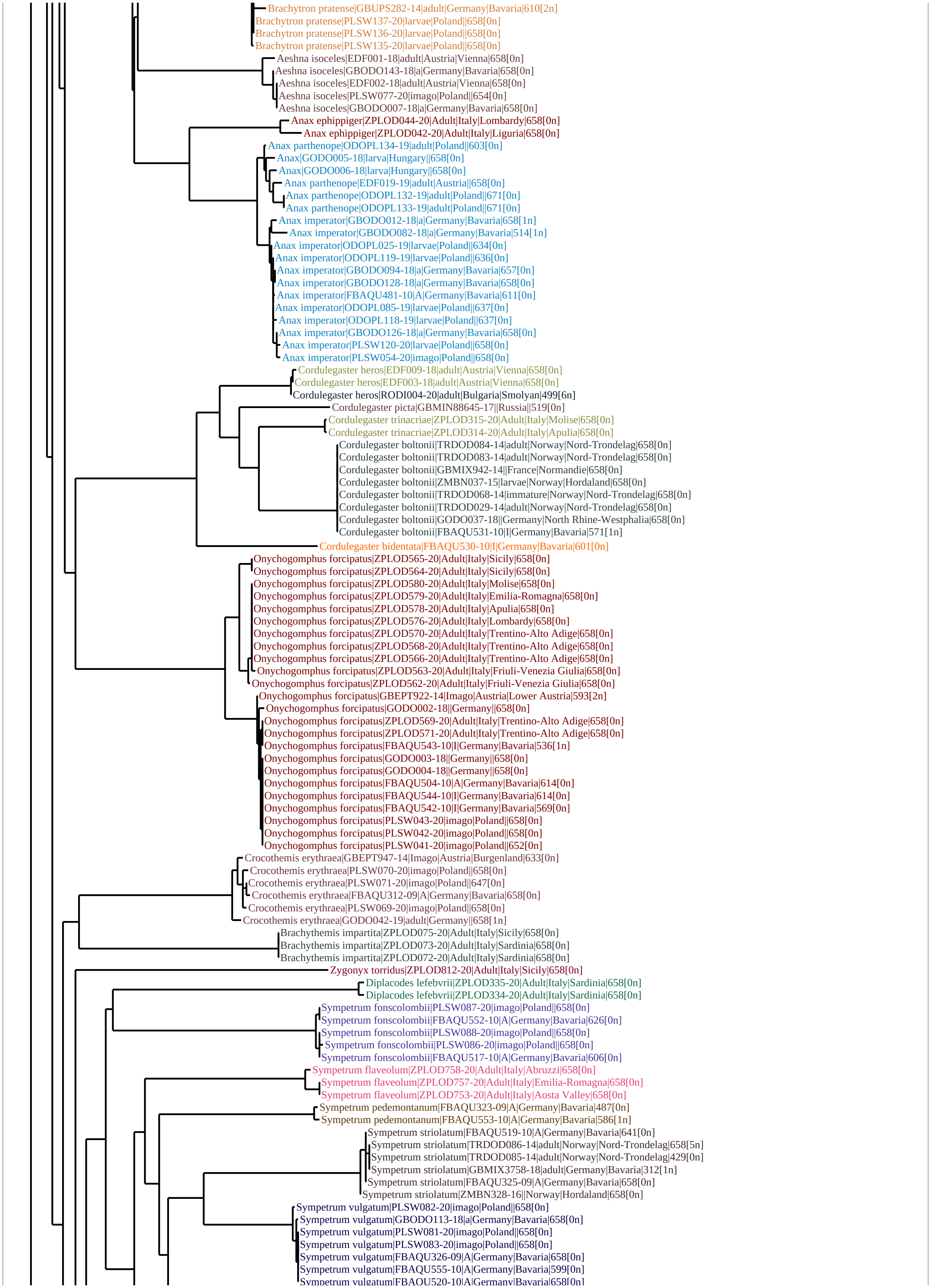
Brachytron pratense|GBUPS282-14|adult|Germany|Bavaria|610[2n]
Brachytron pratense|PLSW137-20|larvae|Poland||658[0n]
Brachytron pratense|PLSW136-20|larvae|Poland||658[0n]
Brachytron pratense|PLSW135-20|larvae|Poland||658[0n]
Aeshna isoceles|EDF001-18|adult|Austria|Vienna|658[0n]
Aeshna isoceles|GBODO143-18|a|Germany|Bavaria|658[0n]
Aeshna isoceles|EDF002-18|adult|Austria|Vienna|658[0n]
Aeshna isoceles|PLSW077-20|imago|Poland||654[0n]
Aeshna isoceles|GBODO007-18|a|Germany|Bavaria|658[0n]
Anax ephippiger|ZPLOD044-20|Adult|Italy|Lombardy|658[0n]
Anax ephippiger|ZPLOD042-20|Adult|Italy|Liguria|658[0n]
Anax parthenope|ODOPL134-19|adult|Poland||603[0n]
Anax|GODO005-18|larva|Hungary||658[0n]
Anax|GODO006-18|larva|Hungary||658[0n]
Anax parthenope|EDF019-19|adult|Austria||658[0n]
Anax parthenope|ODOPL132-19|adult|Poland||671[0n]
Anax parthenope|ODOPL133-19|adult|Poland||671[0n]
Anax imperator|GBODO012-18|a|Germany|Bavaria|658[1n]
Anax imperator|GBODO082-18|a|Germany|Bavaria|514[1n]
Anax imperator|ODOPL025-19|larvae|Poland||634[0n]
Anax imperator|ODOPL119-19|larvae|Poland||636[0n]
Anax imperator|GBODO094-18|a|Germany|Bavaria|657[0n]
Anax imperator|GBODO128-18|a|Germany|Bavaria|658[0n]
Anax imperator|FBAQU481-10|A|Germany|Bavaria|611[0n]
Anax imperator|ODOPL085-19|larvae|Poland||637[0n]
Anax imperator|ODOPL118-19|larvae|Poland||637[0n]
Anax imperator|GBODO126-18|a|Germany|Bavaria|658[0n]
Anax imperator|PLSW120-20|larvae|Poland||658[0n]
Anax imperator|PLSW054-20|imago|Poland||658[0n]
Cordulegaster heros|EDF009-18|adult|Austria|Vienna|658[0n]
Cordulegaster heros|EDF003-18|adult|Austria|Vienna|658[0n]
Cordulegaster heros|RODI004-20|adult|Bulgaria|Smolyan|499[6n]
Cordulegaster picta|GBMIN88645-17||Russia||519[0n]
Cordulegaster trinacriae|ZPLOD315-20|Adult|Italy|Molise|658[0n]
Cordulegaster trinacriae|ZPLOD314-20|Adult|Italy|Apulia|658[0n]
Cordulegaster boltonii|TRDOD084-14|adult|Norway|Nord-Trondelag|658[0n]
Cordulegaster boltonii|TRDOD083-14|adult|Norway|Nord-Trondelag|658[0n]
Cordulegaster boltonii|GBMIX942-14||France|Normandie|658[0n]
Cordulegaster boltonii|ZMBN037-15|larvae|Norway|Hordaland|658[0n]
Cordulegaster boltonii|TRDOD068-14|immature|Norway|Nord-Trondelag|658[0n]
Cordulegaster boltonii|TRDOD029-14|adult|Norway|Nord-Trondelag|658[0n]
Cordulegaster boltonii|GODO037-18||Germany|North Rhine-Westphalia|658[0n]
Cordulegaster boltonii|FBAQU531-10|I|Germany|Bavaria|571[1n]
Cordulegaster bidentata|FBAQU530-10|I|Germany|Bavaria|601[0n]
Onychogomphus forcipatus|ZPLOD565-20|Adult|Italy|Sicily|658[0n]
Onychogomphus forcipatus|ZPLOD564-20|Adult|Italy|Sicily|658[0n]
Onychogomphus forcipatus|ZPLOD580-20|Adult|Italy|Molise|658[0n]
Onychogomphus forcipatus|ZPLOD579-20|Adult|Italy|Emilia-Romagna|658[0n]
Onychogomphus forcipatus|ZPLOD578-20|Adult|Italy|Apulia|658[0n]
Onychogomphus forcipatus|ZPLOD576-20|Adult|Italy|Lombardy|658[0n]
Onychogomphus forcipatus|ZPLOD570-20|Adult|Italy|Trentino-Alto Adige|658[0n]
Onychogomphus forcipatus|ZPLOD568-20|Adult|Italy|Trentino-Alto Adige|658[0n]
Onychogomphus forcipatus|ZPLOD566-20|Adult|Italy|Trentino-Alto Adige|658[0n]
Onychogomphus forcipatus|ZPLOD563-20|Adult|Italy|Friuli-Venezia Giulia|658[0n]
Onychogomphus forcipatus|ZPLOD562-20|Adult|Italy|Friuli-Venezia Giulia|658[0n]
Onychogomphus forcipatus|GBEPT922-14|Imago|Austria|Lower Austria|593[2n]
Onychogomphus forcipatus|GODO002-18||Germany||658[0n]
Onychogomphus forcipatus|ZPLOD569-20|Adult|Italy|Trentino-Alto Adige|658[0n]
Onychogomphus forcipatus|ZPLOD571-20|Adult|Italy|Trentino-Alto Adige|658[0n]
Onychogomphus forcipatus|FBAQU543-10|I|Germany|Bavaria|536[1n]
Onychogomphus forcipatus|GODO003-18||Germany||658[0n]
Onychogomphus forcipatus|GODO004-18||Germany||658[0n]
Onychogomphus forcipatus|FBAQU504-10|A|Germany|Bavaria|614[0n]
Onychogomphus forcipatus|FBAQU544-10|I|Germany|Bavaria|614[0n]
Onychogomphus forcipatus|FBAQU542-10|I|Germany|Bavaria|569[0n]
Onychogomphus forcipatus|PLSW043-20|imago|Poland||658[0n]
Onychogomphus forcipatus|PLSW042-20|imago|Poland||658[0n]
Onychogomphus forcipatus|PLSW041-20|imago|Poland||652[0n]
Crocothemis erythraea|GBEPT947-14|Imago|Austria|Burgenland|633[0n]
Crocothemis erythraea|PLSW070-20|imago|Poland||658[0n]
Crocothemis erythraea|PLSW071-20|imago|Poland||647[0n]
Crocothemis erythraea|FBAQU312-09|A|Germany|Bavaria|658[0n]
Crocothemis erythraea|PLSW069-20|imago|Poland||658[0n]
Crocothemis erythraea|GODO042-19|adult|Germany||658[1n]
Brachythemis impartita|ZPLOD075-20|Adult|Italy|Sicily|658[0n]
Brachythemis impartita|ZPLOD073-20|Adult|Italy|Sardinia|658[0n]
Brachythemis impartita|ZPLOD072-20|Adult|Italy|Sardinia|658[0n]
Zygonyx torridus|ZPLOD812-20|Adult|Italy|Sicily|658[0n]
Diplacodes lefebvrii|ZPLOD335-20|Adult|Italy|Sardinia|658[0n]
Diplacodes lefebvrii|ZPLOD334-20|Adult|Italy|Sardinia|658[0n]
Sympetrum fonscolombii|PLSW087-20|imago|Poland||658[0n]
Sympetrum fonscolombii|FBAQU552-10|A|Germany|Bavaria|626[0n]
Sympetrum fonscolombii|PLSW088-20|imago|Poland||658[0n]
Sympetrum fonscolombii|PLSW086-20|imago|Poland||658[0n]
Sympetrum fonscolombii|FBAQU517-10|A|Germany|Bavaria|606[0n]
Sympetrum flaveolum|ZPLOD758-20|Adult|Italy|Abruzzi|658[0n]
Sympetrum flaveolum|ZPLOD757-20|Adult|Italy|Emilia-Romagna|658[0n]
Sympetrum flaveolum|ZPLOD753-20|Adult|Italy|Aosta Valley|658[0n]
Sympetrum pedemontanum|FBAQU323-09|A|Germany|Bavaria|487[0n]
Sympetrum pedemontanum|FBAQU553-10|A|Germany|Bavaria|586[1n]
Sympetrum striolatum|FBAQU519-10|A|Germany|Bavaria|641[0n]
Sympetrum striolatum|TRDOD086-14|adult|Norway|Nord-Trondelag|658[5n]
Sympetrum striolatum|TRDOD085-14|adult|Norway|Nord-Trondelag|429[0n]
Sympetrum striolatum|GBMIX3758-18|adult|Germany|Bavaria|312[1n]
Sympetrum striolatum|FBAQU325-09|A|Germany|Bavaria|658[0n]
Sympetrum striolatum|ZMBN328-16||Norway|Hordaland|658[0n]
Sympetrum vulgatum|PLSW082-20|imago|Poland||658[0n]
Sympetrum vulgatum|GBODO113-18|a|Germany|Bavaria|658[0n]
Sympetrum vulgatum|PLSW081-20|imago|Poland||658[0n]
Sympetrum vulgatum|PLSW083-20|imago|Poland||658[0n]
Sympetrum vulgatum|FBAQU326-09|A|Germany|Bavaria|658[0n]
Sympetrum vulgatum|FBAQU555-10|A|Germany|Bavaria|599[0n]
Sympetrum vulgatum|FBAQU520-10|A|Germany|Bavaria|658[0n]

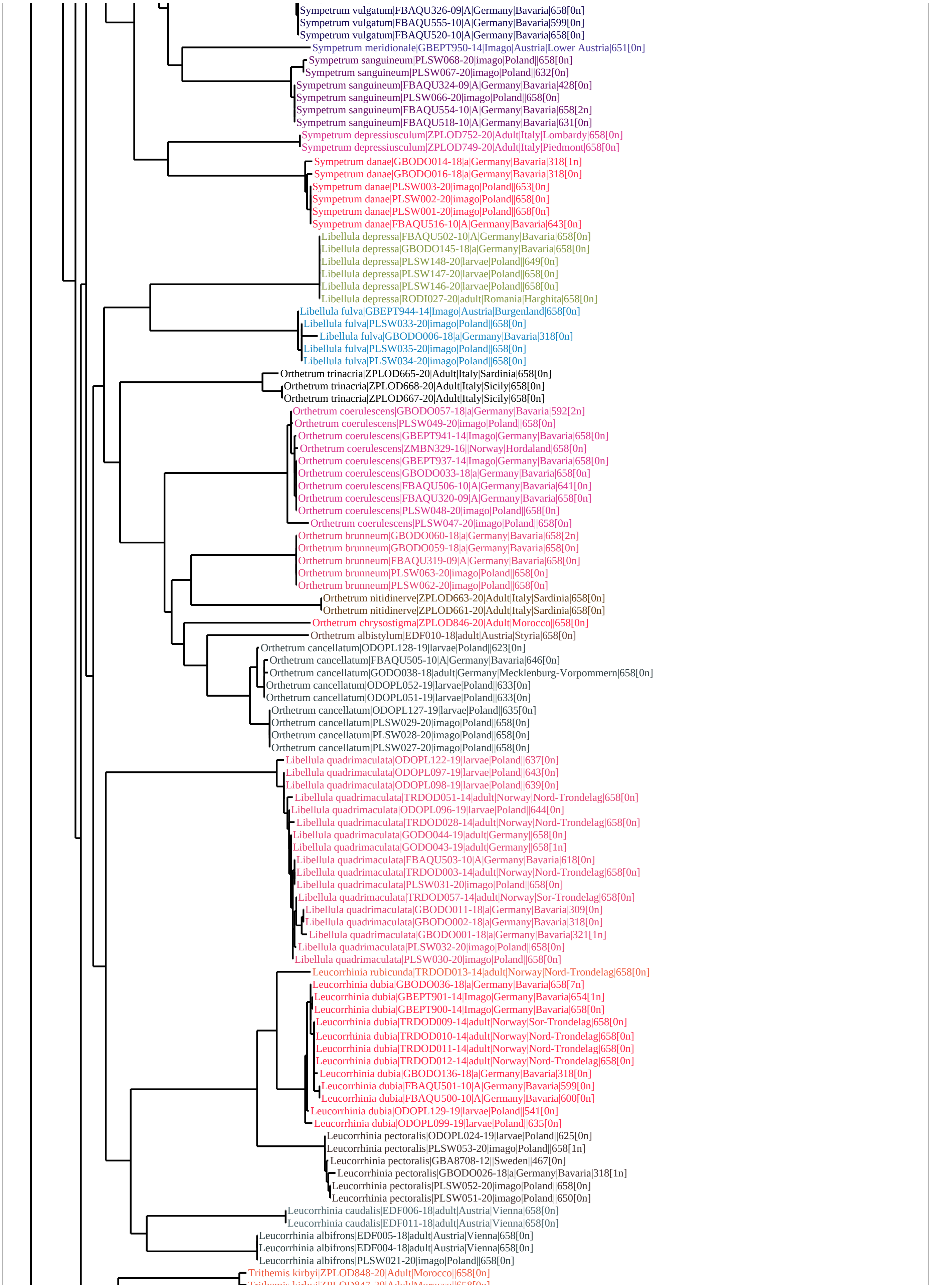
Sympetrum vulgatum|FBAQU326-09|A|Germany|Bavaria|658[0n]
Sympetrum vulgatum|FBAQU555-10|A|Germany|Bavaria|599[0n]
Sympetrum vulgatum|FBAQU520-10|A|Germany|Bavaria|658[0n]
Sympetrum meridionale|GBEPT950-14|Imago|Austria|Lower Austria|651[0n]
Sympetrum sanguineum|PLSW068-20|imago|Poland||658[0n]
Sympetrum sanguineum|PLSW067-20|imago|Poland||632[0n]
Sympetrum sanguineum|FBAQU324-09|A|Germany|Bavaria|428[0n]
Sympetrum sanguineum|PLSW066-20|imago|Poland||658[0n]
Sympetrum sanguineum|FBAQU554-10|A|Germany|Bavaria|658[2n]
Sympetrum sanguineum|FBAQU518-10|A|Germany|Bavaria|631[0n]
Sympetrum depressiusculum|ZPLOD752-20|Adult|Italy|Lombardy|658[0n]
Sympetrum depressiusculum|ZPLOD749-20|Adult|Italy|Piedmont|658[0n]
Sympetrum danae|GBODO014-18|a|Germany|Bavaria|318[1n]
Sympetrum danae|GBODO016-18|a|Germany|Bavaria|318[0n]
Sympetrum danae|PLSW003-20|imago|Poland||653[0n]
Sympetrum danae|PLSW002-20|imago|Poland||658[0n]
Sympetrum danae|PLSW001-20|imago|Poland||658[0n]
Sympetrum danae|FBAQU516-10|A|Germany|Bavaria|643[0n]
Libellula depressa|FBAQU502-10|A|Germany|Bavaria|658[0n]
Libellula depressa|GBODO145-18|a|Germany|Bavaria|658[0n]
Libellula depressa|PLSW148-20|larvae|Poland||649[0n]
Libellula depressa|PLSW147-20|larvae|Poland||658[0n]
Libellula depressa|PLSW146-20|larvae|Poland||658[0n]
Libellula depressa|RODI027-20|adult|Romania|Harghita|658[0n]
Libellula fulva|GBEPT944-14|Imago|Austria|Burgenland|658[0n]
Libellula fulva|PLSW033-20|imago|Poland||658[0n]
Libellula fulva|GBODO006-18|a|Germany|Bavaria|318[0n]
Libellula fulva|PLSW035-20|imago|Poland||658[0n]
Libellula fulva|PLSW034-20|imago|Poland||658[0n]
Orthetrum trinacria|ZPLOD665-20|Adult|Italy|Sardinia|658[0n]
Orthetrum trinacria|ZPLOD668-20|Adult|Italy|Sicily|658[0n]
Orthetrum trinacria|ZPLOD667-20|Adult|Italy|Sicily|658[0n]
Orthetrum coerulescens|GBODO057-18|a|Germany|Bavaria|592[2n]
Orthetrum coerulescens|PLSW049-20|imago|Poland||658[0n]
Orthetrum coerulescens|GBEPT941-14|Imago|Germany|Bavaria|658[0n]
Orthetrum coerulescens|ZMBN329-16||Norway|Hordaland|658[0n]
Orthetrum coerulescens|GBEPT937-14|Imago|Germany|Bavaria|658[0n]
Orthetrum coerulescens|GBODO033-18|a|Germany|Bavaria|658[0n]
Orthetrum coerulescens|FBAQU506-10|A|Germany|Bavaria|641[0n]
Orthetrum coerulescens|FBAQU320-09|A|Germany|Bavaria|658[0n]
Orthetrum coerulescens|PLSW048-20|imago|Poland||658[0n]
Orthetrum coerulescens|PLSW047-20|imago|Poland||658[0n]
Orthetrum brunneum|GBODO060-18|a|Germany|Bavaria|658[2n]
Orthetrum brunneum|GBODO059-18|a|Germany|Bavaria|658[0n]
Orthetrum brunneum|FBAQU319-09|A|Germany|Bavaria|658[0n]
Orthetrum brunneum|PLSW063-20|imago|Poland||658[0n]
Orthetrum brunneum|PLSW062-20|imago|Poland||658[0n]
Orthetrum nitidinerve|ZPLOD663-20|Adult|Italy|Sardinia|658[0n]
Orthetrum nitidinerve|ZPLOD661-20|Adult|Italy|Sardinia|658[0n]
Orthetrum chrysostigma|ZPLOD846-20|Adult|Morocco||658[0n]
Orthetrum albistylum|EDF010-18|adult|Austria|Styria|658[0n]
Orthetrum cancellatum|ODOPL128-19|larvae|Poland||623[0n]
Orthetrum cancellatum|FBAQU505-10|A|Germany|Bavaria|646[0n]
Orthetrum cancellatum|GODO038-18|adult|Germany|Mecklenburg-Vorpommern|658[0n]
Orthetrum cancellatum|ODOPL052-19|larvae|Poland||633[0n]
Orthetrum cancellatum|ODOPL051-19|larvae|Poland||633[0n]
Orthetrum cancellatum|ODOPL127-19|larvae|Poland||635[0n]
Orthetrum cancellatum|PLSW029-20|imago|Poland||658[0n]
Orthetrum cancellatum|PLSW028-20|imago|Poland||658[0n]
Orthetrum cancellatum|PLSW027-20|imago|Poland||658[0n]
Libellula quadrimaculata|ODOPL122-19|larvae|Poland||637[0n]
Libellula quadrimaculata|ODOPL097-19|larvae|Poland||643[0n]
Libellula quadrimaculata|ODOPL098-19|larvae|Poland||639[0n]
Libellula quadrimaculata|TRDOD051-14|adult|Norway|Nord-Trondelag|658[0n]
Libellula quadrimaculata|ODOPL096-19|larvae|Poland||644[0n]
Libellula quadrimaculata|TRDOD028-14|adult|Norway|Nord-Trondelag|658[0n]
Libellula quadrimaculata|GODO044-19|adult|Germany||658[0n]
Libellula quadrimaculata|GODO043-19|adult|Germany||658[1n]
Libellula quadrimaculata|FBAQU503-10|A|Germany|Bavaria|618[0n]
Libellula quadrimaculata|TRDOD003-14|adult|Norway|Nord-Trondelag|658[0n]
Libellula quadrimaculata|PLSW031-20|imago|Poland||658[0n]
Libellula quadrimaculata|TRDOD057-14|adult|Norway|Sor-Trondelag|658[0n]
Libellula quadrimaculata|GBODO011-18|a|Germany|Bavaria|309[0n]
Libellula quadrimaculata|GBODO002-18|a|Germany|Bavaria|318[0n]
Libellula quadrimaculata|GBODO001-18|a|Germany|Bavaria|321[1n]
Libellula quadrimaculata|PLSW032-20|imago|Poland||658[0n]
Libellula quadrimaculata|PLSW030-20|imago|Poland||658[0n]
Leucorrhinia rubicunda|TRDOD013-14|adult|Norway|Nord-Trondelag|658[0n]
Leucorrhinia dubia|GBODO036-18|a|Germany|Bavaria|658[7n]
Leucorrhinia dubia|GBEPT901-14|Imago|Germany|Bavaria|654[1n]
Leucorrhinia dubia|GBEPT900-14|Imago|Germany|Bavaria|658[0n]
Leucorrhinia dubia|TRDOD009-14|adult|Norway|Sor-Trondelag|658[0n]
Leucorrhinia dubia|TRDOD010-14|adult|Norway|Nord-Trondelag|658[0n]
Leucorrhinia dubia|TRDOD011-14|adult|Norway|Nord-Trondelag|658[0n]
Leucorrhinia dubia|TRDOD012-14|adult|Norway|Nord-Trondelag|658[0n]
Leucorrhinia dubia|GBODO136-18|a|Germany|Bavaria|318[0n]
Leucorrhinia dubia|FBAQU501-10|A|Germany|Bavaria|599[0n]
Leucorrhinia dubia|FBAQU500-10|A|Germany|Bavaria|600[0n]
Leucorrhinia dubia|ODOPL129-19|larvae|Poland||541[0n]
Leucorrhinia dubia|ODOPL099-19|larvae|Poland||635[0n]
Leucorrhinia pectoralis|ODOPL024-19|larvae|Poland||625[0n]
Leucorrhinia pectoralis|PLSW053-20|imago|Poland||658[1n]
Leucorrhinia pectoralis|GBA8708-12||Sweden||467[0n]
Leucorrhinia pectoralis|GBODO026-18|a|Germany|Bavaria|318[1n]
Leucorrhinia pectoralis|PLSW052-20|imago|Poland||658[0n]
Leucorrhinia pectoralis|PLSW051-20|imago|Poland||650[0n]
Leucorrhinia caudalis|EDF006-18|adult|Austria|Vienna|658[0n]
Leucorrhinia caudalis|EDF011-18|adult|Austria|Vienna|658[0n]
Leucorrhinia albifrons|EDF005-18|adult|Austria|Vienna|658[0n]
Leucorrhinia albifrons|EDF004-18|adult|Austria|Vienna|658[0n]
Leucorrhinia albifrons|PLSW021-20|imago|Poland||658[0n]
Trithemis kirbyi|ZPLOD848-20|Adult|Morocco||658[0n]

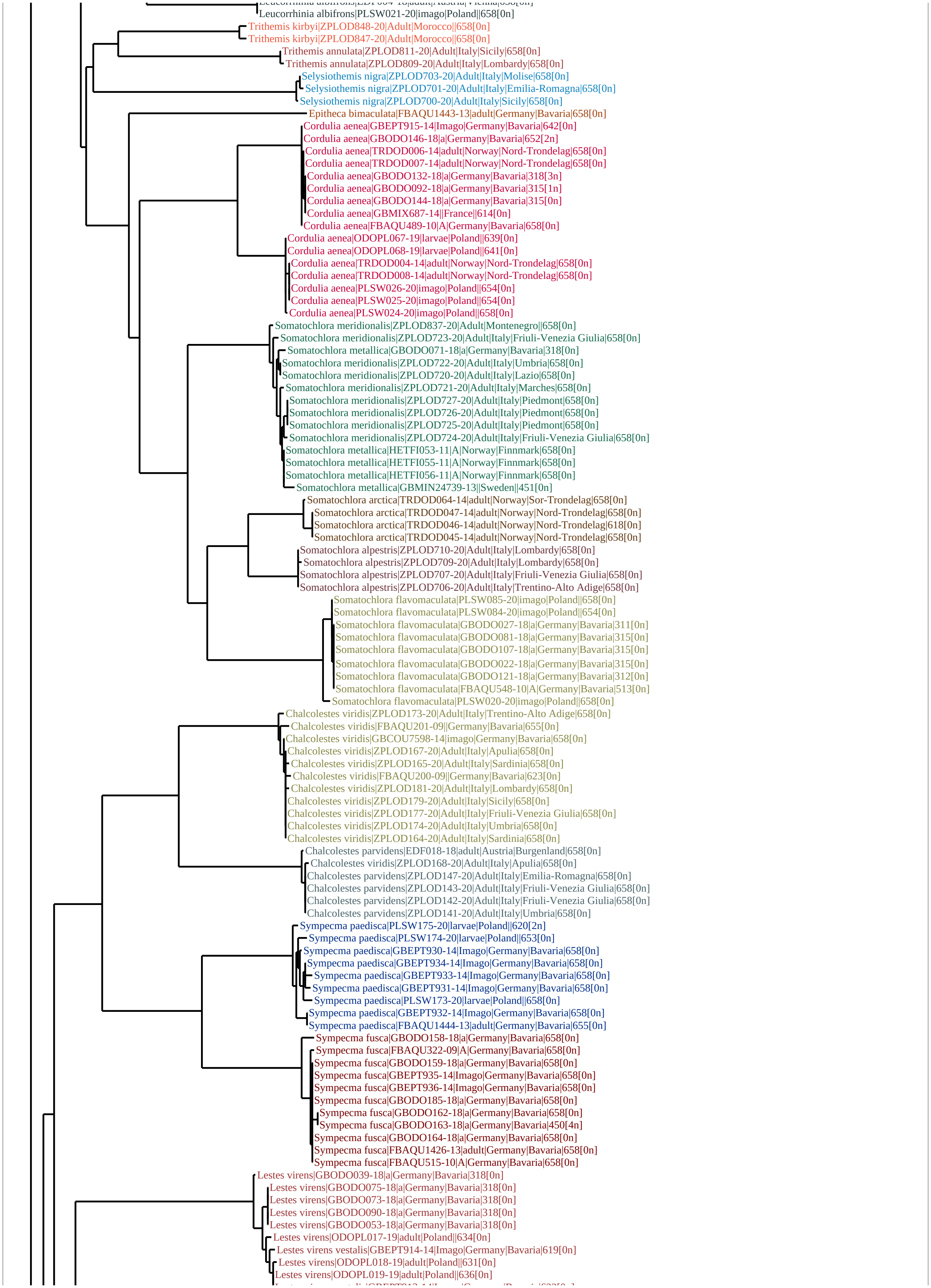
Leucorrhinia albifrons|PLSW021-20|imago|Poland||658[0n]
Trithemis kirbyi|ZPLOD848-20|Adult|Morocco||658[0n]
Trithemis kirbyi|ZPLOD847-20|Adult|Morocco||658[0n]
Trithemis annulata|ZPLOD811-20|Adult|Italy|Sicily|658[0n]
Trithemis annulata|ZPLOD809-20|Adult|Italy|Lombardy|658[0n]
Selysiothemis nigra|ZPLOD703-20|Adult|Italy|Molise|658[0n]
Selysiothemis nigra|ZPLOD701-20|Adult|Italy|Emilia-Romagna|658[0n]
Selysiothemis nigra|ZPLOD700-20|Adult|Italy|Sicily|658[0n]
Epitheca bimaculata|FBAQU1443-13|adult|Germany|Bavaria|658[0n]
Cordulia aenea|GBEPT915-14|Imago|Germany|Bavaria|642[0n]
Cordulia aenea|GBODO146-18|a|Germany|Bavaria|652[2n]
Cordulia aenea|TRDOD006-14|adult|Norway|Nord-Trondelag|658[0n]
Cordulia aenea|TRDOD007-14|adult|Norway|Nord-Trondelag|658[0n]
Cordulia aenea|GBODO132-18|a|Germany|Bavaria|318[3n]
Cordulia aenea|GBODO092-18|a|Germany|Bavaria|315[1n]
Cordulia aenea|GBODO144-18|a|Germany|Bavaria|315[0n]
Cordulia aenea|GBMIX687-14||France||614[0n]
Cordulia aenea|FBAQU489-10|A|Germany|Bavaria|658[0n]
Cordulia aenea|ODOPL067-19|larvae|Poland||639[0n]
Cordulia aenea|ODOPL068-19|larvae|Poland||641[0n]
Cordulia aenea|TRDOD004-14|adult|Norway|Nord-Trondelag|658[0n]
Cordulia aenea|TRDOD008-14|adult|Norway|Nord-Trondelag|658[0n]
Cordulia aenea|PLSW026-20|imago|Poland||654[0n]
Cordulia aenea|PLSW025-20|imago|Poland||654[0n]
Cordulia aenea|PLSW024-20|imago|Poland||658[0n]
Somatochlora meridionalis|ZPLOD837-20|Adult|Montenegro||658[0n]
Somatochlora meridionalis|ZPLOD723-20|Adult|Italy|Friuli-Venezia Giulia|658[0n]
Somatochlora metallica|GBODO071-18|a|Germany|Bavaria|318[0n]
Somatochlora meridionalis|ZPLOD722-20|Adult|Italy|Umbria|658[0n]
Somatochlora meridionalis|ZPLOD720-20|Adult|Italy|Lazio|658[0n]
Somatochlora meridionalis|ZPLOD721-20|Adult|Italy|Marches|658[0n]
Somatochlora meridionalis|ZPLOD727-20|Adult|Italy|Piedmont|658[0n]
Somatochlora meridionalis|ZPLOD726-20|Adult|Italy|Piedmont|658[0n]
Somatochlora meridionalis|ZPLOD725-20|Adult|Italy|Piedmont|658[0n]
Somatochlora meridionalis|ZPLOD724-20|Adult|Italy|Friuli-Venezia Giulia|658[0n]
Somatochlora metallica|HETFI053-11|A|Norway|Finnmark|658[0n]
Somatochlora metallica|HETFI055-11|A|Norway|Finnmark|658[0n]
Somatochlora metallica|HETFI056-11|A|Norway|Finnmark|658[0n]
Somatochlora metallica|GBMIN24739-13||Sweden||451[0n]
Somatochlora arctica|TRDOD064-14|adult|Norway|Sor-Trondelag|658[0n]
Somatochlora arctica|TRDOD047-14|adult|Norway|Nord-Trondelag|658[0n]
Somatochlora arctica|TRDOD046-14|adult|Norway|Nord-Trondelag|618[0n]
Somatochlora arctica|TRDOD045-14|adult|Norway|Nord-Trondelag|658[0n]
Somatochlora alpestris|ZPLOD710-20|Adult|Italy|Lombardy|658[0n]
Somatochlora alpestris|ZPLOD709-20|Adult|Italy|Lombardy|658[0n]
Somatochlora alpestris|ZPLOD707-20|Adult|Italy|Friuli-Venezia Giulia|658[0n]
Somatochlora alpestris|ZPLOD706-20|Adult|Italy|Trentino-Alto Adige|658[0n]
Somatochlora flavomaculata|PLSW085-20|imago|Poland||658[0n]
Somatochlora flavomaculata|PLSW084-20|imago|Poland||654[0n]
Somatochlora flavomaculata|GBODO027-18|a|Germany|Bavaria|311[0n]
Somatochlora flavomaculata|GBODO081-18|a|Germany|Bavaria|315[0n]
Somatochlora flavomaculata|GBODO107-18|a|Germany|Bavaria|315[0n]
Somatochlora flavomaculata|GBODO022-18|a|Germany|Bavaria|315[0n]
Somatochlora flavomaculata|GBODO121-18|a|Germany|Bavaria|312[0n]
Somatochlora flavomaculata|FBAQU548-10|A|Germany|Bavaria|513[0n]
Somatochlora flavomaculata|PLSW020-20|imago|Poland||658[0n]
Chalcolestes viridis|ZPLOD173-20|Adult|Italy|Trentino-Alto Adige|658[0n]
Chalcolestes viridis|FBAQU201-09||Germany|Bavaria|655[0n]
Chalcolestes viridis|GBCOU7598-14|imago|Germany|Bavaria|658[0n]
Chalcolestes viridis|ZPLOD167-20|Adult|Italy|Apulia|658[0n]
Chalcolestes viridis|ZPLOD165-20|Adult|Italy|Sardinia|658[0n]
Chalcolestes viridis|FBAQU200-09||Germany|Bavaria|623[0n]
Chalcolestes viridis|ZPLOD181-20|Adult|Italy|Lombardy|658[0n]
Chalcolestes viridis|ZPLOD179-20|Adult|Italy|Sicily|658[0n]
Chalcolestes viridis|ZPLOD177-20|Adult|Italy|Friuli-Venezia Giulia|658[0n]
Chalcolestes viridis|ZPLOD174-20|Adult|Italy|Umbria|658[0n]
Chalcolestes viridis|ZPLOD164-20|Adult|Italy|Sardinia|658[0n]
Chalcolestes parvidens|EDF018-18|adult|Austria|Burgenland|658[0n]
Chalcolestes viridis|ZPLOD168-20|Adult|Italy|Apulia|658[0n]
Chalcolestes parvidens|ZPLOD147-20|Adult|Italy|Emilia-Romagna|658[0n]
Chalcolestes parvidens|ZPLOD143-20|Adult|Italy|Friuli-Venezia Giulia|658[0n]
Chalcolestes parvidens|ZPLOD142-20|Adult|Italy|Friuli-Venezia Giulia|658[0n]
Chalcolestes parvidens|ZPLOD141-20|Adult|Italy|Umbria|658[0n]
Sympecma paedisca|PLSW175-20|larvae|Poland||620[2n]
Sympecma paedisca|PLSW174-20|larvae|Poland||653[0n]
Sympecma paedisca|GBEPT930-14|Imago|Germany|Bavaria|658[0n]
Sympecma paedisca|GBEPT934-14|Imago|Germany|Bavaria|658[0n]
Sympecma paedisca|GBEPT933-14|Imago|Germany|Bavaria|658[0n]
Sympecma paedisca|GBEPT931-14|Imago|Germany|Bavaria|658[0n]
Sympecma paedisca|PLSW173-20|larvae|Poland||658[0n]
Sympecma paedisca|GBEPT932-14|Imago|Germany|Bavaria|658[0n]
Sympecma paedisca|FBAQU1444-13|adult|Germany|Bavaria|655[0n]
Sympecma fusca|GBODO158-18|a|Germany|Bavaria|658[0n]
Sympecma fusca|FBAQU322-09|A|Germany|Bavaria|658[0n]
Sympecma fusca|GBODO159-18|a|Germany|Bavaria|658[0n]
Sympecma fusca|GBEPT935-14|Imago|Germany|Bavaria|658[0n]
Sympecma fusca|GBEPT936-14|Imago|Germany|Bavaria|658[0n]
Sympecma fusca|GBODO185-18|a|Germany|Bavaria|658[0n]
Sympecma fusca|GBODO162-18|a|Germany|Bavaria|658[0n]
Sympecma fusca|GBODO163-18|a|Germany|Bavaria|450[4n]
Sympecma fusca|GBODO164-18|a|Germany|Bavaria|658[0n]
Sympecma fusca|FBAQU1426-13|adult|Germany|Bavaria|658[0n]
Sympecma fusca|FBAQU515-10|A|Germany|Bavaria|658[0n]
Lestes virens|GBODO039-18|a|Germany|Bavaria|318[0n]
Lestes virens|GBODO075-18|a|Germany|Bavaria|318[0n]
Lestes virens|GBODO073-18|a|Germany|Bavaria|318[0n]
Lestes virens|GBODO090-18|a|Germany|Bavaria|318[0n]
Lestes virens|GBODO053-18|a|Germany|Bavaria|318[0n]
Lestes virens|ODOPL017-19|adult|Poland||634[0n]
Lestes virens vestalis|GBEPT914-14|Imago|Germany|Bavaria|619[0n]
Lestes virens|ODOPL018-19|adult|Poland||631[0n]
Lestes virens|ODOPL019-19|adult|Poland||636[0n]

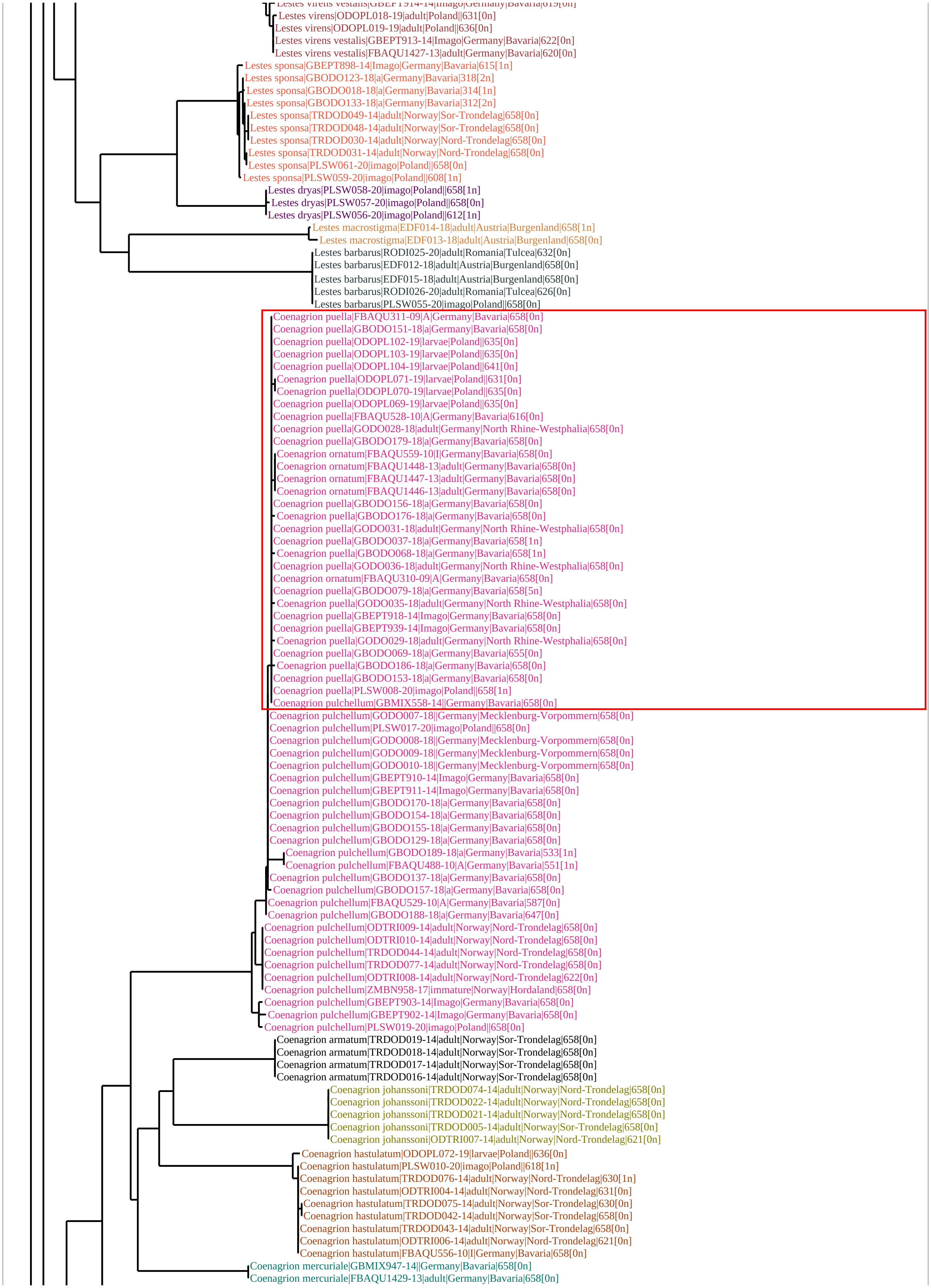
Lestes virens vestalis|GBEPT914-14|Imago|Germany|Bavaria|619[0n]
Lestes virens|ODOPL018-19|adult|Poland||631[0n]
Lestes virens|ODOPL019-19|adult|Poland||636[0n]
Lestes virens vestalis|GBEPT913-14|Imago|Germany|Bavaria|622[0n]
Lestes virens vestalis|FBAQU1427-13|adult|Germany|Bavaria|620[0n]
Lestes sponsa|GBEPT898-14|Imago|Germany|Bavaria|615[1n]
Lestes sponsa|GBODO123-18|a|Germany|Bavaria|318[2n]
Lestes sponsa|GBODO018-18|a|Germany|Bavaria|314[1n]
Lestes sponsa|GBODO133-18|a|Germany|Bavaria|312[2n]
Lestes sponsa|TRDOD049-14|adult|Norway|Sor-Trondelag|658[0n]
Lestes sponsa|TRDOD048-14|adult|Norway|Sor-Trondelag|658[0n]
Lestes sponsa|TRDOD030-14|adult|Norway|Nord-Trondelag|658[0n]
Lestes sponsa|TRDOD031-14|adult|Norway|Nord-Trondelag|658[0n]
Lestes sponsa|PLSW061-20|imago|Poland||658[0n]
Lestes sponsa|PLSW059-20|imago|Poland||608[1n]
Lestes dryas|PLSW058-20|imago|Poland||658[1n]
Lestes dryas|PLSW057-20|imago|Poland||658[0n]
Lestes dryas|PLSW056-20|imago|Poland||612[1n]
Lestes macrostigma|EDF014-18|adult|Austria|Burgenland|658[1n]
Lestes macrostigma|EDF013-18|adult|Austria|Burgenland|658[0n]
Lestes barbarus|RODI025-20|adult|Romania|Tulcea|632[0n]
Lestes barbarus|EDF012-18|adult|Austria|Burgenland|658[0n]
Lestes barbarus|EDF015-18|adult|Austria|Burgenland|658[0n]
Lestes barbarus|RODI026-20|adult|Romania|Tulcea|626[0n]
Lestes barbarus|PLSW055-20|imago|Poland||658[0n]
Coenagrion puella|FBAQU311-09|A|Germany|Bavaria|658[0n]
Coenagrion puella|GBODO151-18|a|Germany|Bavaria|658[0n]
Coenagrion puella|ODOPL102-19|larvae|Poland||635[0n]
Coenagrion puella|ODOPL103-19|larvae|Poland||635[0n]
Coenagrion puella|ODOPL104-19|larvae|Poland||641[0n]
Coenagrion puella|ODOPL071-19|larvae|Poland||631[0n]
Coenagrion puella|ODOPL070-19|larvae|Poland||635[0n]
Coenagrion puella|ODOPL069-19|larvae|Poland||635[0n]
Coenagrion puella|FBAQU528-10|A|Germany|Bavaria|616[0n]
Coenagrion puella|GODO028-18|adult|Germany|North Rhine-Westphalia|658[0n]
Coenagrion puella|GBODO179-18|a|Germany|Bavaria|658[0n]
Coenagrion ornatum|FBAQU559-10|I|Germany|Bavaria|658[0n]
Coenagrion ornatum|FBAQU1448-13|adult|Germany|Bavaria|658[0n]
Coenagrion ornatum|FBAQU1447-13|adult|Germany|Bavaria|658[0n]
Coenagrion ornatum|FBAQU1446-13|adult|Germany|Bavaria|658[0n]
Coenagrion puella|GBODO156-18|a|Germany|Bavaria|658[0n]
Coenagrion puella|GBODO176-18|a|Germany|Bavaria|658[0n]
Coenagrion puella|GODO031-18|adult|Germany|North Rhine-Westphalia|658[0n]
Coenagrion puella|GBODO037-18|a|Germany|Bavaria|658[1n]
Coenagrion puella|GBODO068-18|a|Germany|Bavaria|658[1n]
Coenagrion puella|GODO036-18|adult|Germany|North Rhine-Westphalia|658[0n]
Coenagrion ornatum|FBAQU310-09|A|Germany|Bavaria|658[0n]
Coenagrion puella|GBODO079-18|a|Germany|Bavaria|658[5n]
Coenagrion puella|GODO035-18|adult|Germany|North Rhine-Westphalia|658[0n]
Coenagrion puella|GBEPT918-14|Imago|Germany|Bavaria|658[0n]
Coenagrion puella|GBEPT939-14|Imago|Germany|Bavaria|658[0n]
Coenagrion puella|GODO029-18|adult|Germany|North Rhine-Westphalia|658[0n]
Coenagrion puella|GBODO069-18|a|Germany|Bavaria|655[0n]
Coenagrion puella|GBODO186-18|a|Germany|Bavaria|658[0n]
Coenagrion puella|GBODO153-18|a|Germany|Bavaria|658[0n]
Coenagrion puella|PLSW008-20|imago|Poland||658[1n]
Coenagrion pulchellum|GBMIX558-14||Germany|Bavaria|658[0n]
Coenagrion pulchellum|GODO007-18||Germany|Mecklenburg-Vorpommern|658[0n]
Coenagrion pulchellum|PLSW017-20|imago|Poland||658[0n]
Coenagrion pulchellum|GODO008-18||Germany|Mecklenburg-Vorpommern|658[0n]
Coenagrion pulchellum|GODO009-18||Germany|Mecklenburg-Vorpommern|658[0n]
Coenagrion pulchellum|GODO010-18||Germany|Mecklenburg-Vorpommern|658[0n]
Coenagrion pulchellum|GBEPT910-14|Imago|Germany|Bavaria|658[0n]
Coenagrion pulchellum|GBEPT911-14|Imago|Germany|Bavaria|658[0n]
Coenagrion pulchellum|GBODO170-18|a|Germany|Bavaria|658[0n]
Coenagrion pulchellum|GBODO154-18|a|Germany|Bavaria|658[0n]
Coenagrion pulchellum|GBODO155-18|a|Germany|Bavaria|658[0n]
Coenagrion pulchellum|GBODO129-18|a|Germany|Bavaria|658[0n]
Coenagrion pulchellum|GBODO189-18|a|Germany|Bavaria|533[1n]
Coenagrion pulchellum|FBAQU488-10|A|Germany|Bavaria|551[1n]
Coenagrion pulchellum|GBODO137-18|a|Germany|Bavaria|658[0n]
Coenagrion pulchellum|GBODO157-18|a|Germany|Bavaria|658[0n]
Coenagrion pulchellum|FBAQU529-10|A|Germany|Bavaria|587[0n]
Coenagrion pulchellum|GBODO188-18|a|Germany|Bavaria|647[0n]
Coenagrion pulchellum|ODTRI009-14|adult|Norway|Nord-Trondelag|658[0n]
Coenagrion pulchellum|ODTRI010-14|adult|Norway|Nord-Trondelag|658[0n]
Coenagrion pulchellum|TRDOD044-14|adult|Norway|Nord-Trondelag|658[0n]
Coenagrion pulchellum|TRDOD077-14|adult|Norway|Nord-Trondelag|658[0n]
Coenagrion pulchellum|ODTRI008-14|adult|Norway|Nord-Trondelag|622[0n]
Coenagrion pulchellum|ZMBN958-17|immature|Norway|Hordaland|658[0n]
Coenagrion pulchellum|GBEPT903-14|Imago|Germany|Bavaria|658[0n]
Coenagrion pulchellum|GBEPT902-14|Imago|Germany|Bavaria|658[0n]
Coenagrion pulchellum|PLSW019-20|imago|Poland||658[0n]
Coenagrion armatum|TRDOD019-14|adult|Norway|Sor-Trondelag|658[0n]
Coenagrion armatum|TRDOD018-14|adult|Norway|Sor-Trondelag|658[0n]
Coenagrion armatum|TRDOD017-14|adult|Norway|Sor-Trondelag|658[0n]
Coenagrion armatum|TRDOD016-14|adult|Norway|Sor-Trondelag|658[0n]
Coenagrion johanssoni|TRDOD074-14|adult|Norway|Nord-Trondelag|658[0n]
Coenagrion johanssoni|TRDOD022-14|adult|Norway|Nord-Trondelag|658[0n]
Coenagrion johanssoni|TRDOD021-14|adult|Norway|Nord-Trondelag|658[0n]
Coenagrion johanssoni|TRDOD005-14|adult|Norway|Sor-Trondelag|658[0n]
Coenagrion johanssoni|ODTRI007-14|adult|Norway|Nord-Trondelag|621[0n]
Coenagrion hastulatum|ODOPL072-19|larvae|Poland||636[0n]
Coenagrion hastulatum|PLSW010-20|imago|Poland||618[1n]
Coenagrion hastulatum|TRDOD076-14|adult|Norway|Nord-Trondelag|630[1n]
Coenagrion hastulatum|ODTRI004-14|adult|Norway|Nord-Trondelag|631[0n]
Coenagrion hastulatum|TRDOD075-14|adult|Norway|Sor-Trondelag|630[0n]
Coenagrion hastulatum|TRDOD042-14|adult|Norway|Sor-Trondelag|658[0n]
Coenagrion hastulatum|TRDOD043-14|adult|Norway|Sor-Trondelag|658[0n]
Coenagrion hastulatum|ODTRI006-14|adult|Norway|Nord-Trondelag|621[0n]
Coenagrion hastulatum|FBAQU556-10|I|Germany|Bavaria|658[0n]
Coenagrion mercuriale|GBMIX947-14||Germany|Bavaria|658[0n]
Coenagrion mercuriale|FBAQU1429-13|adult|Germany|Bavaria|658[0n]

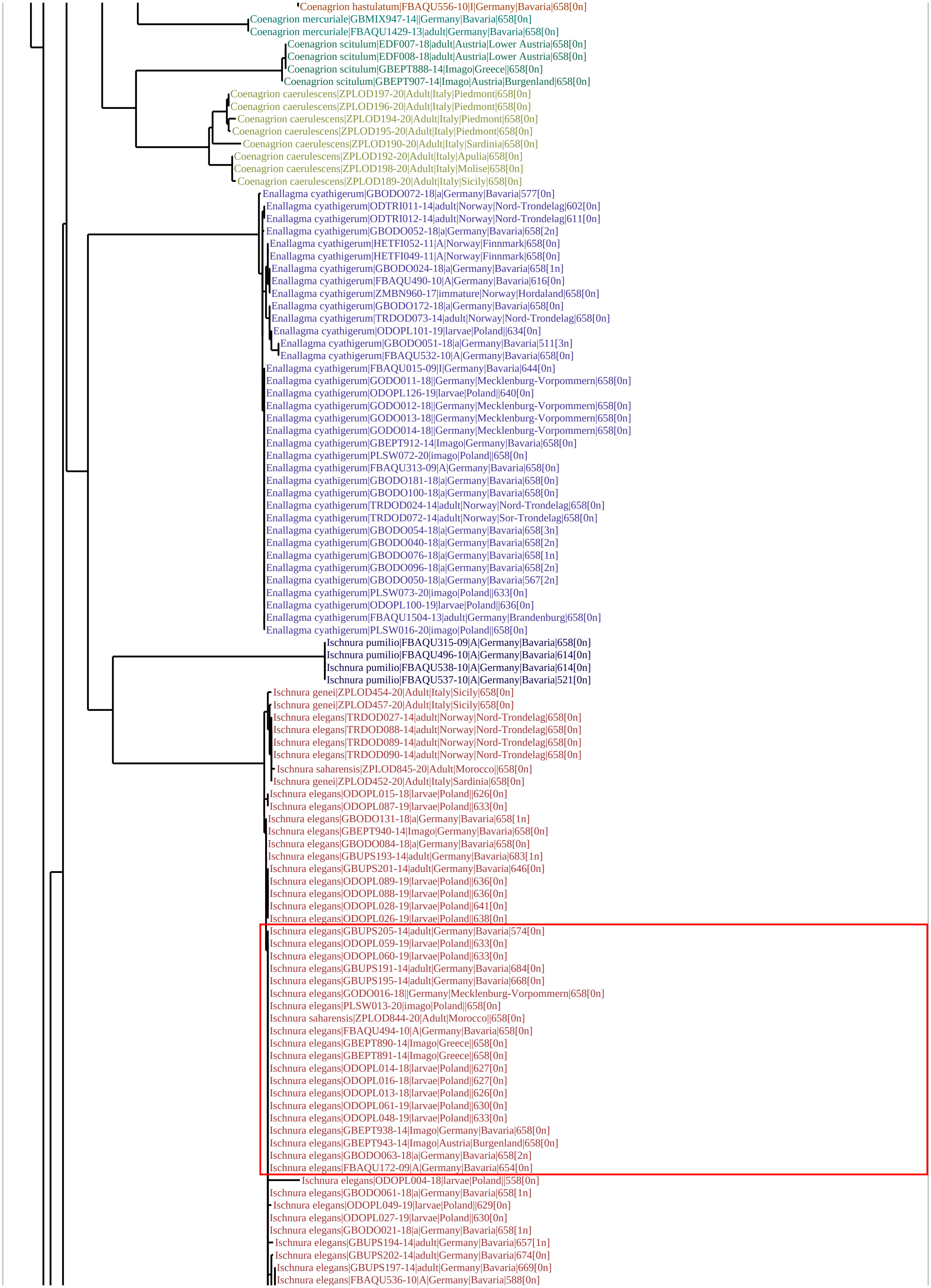

Coenagrion hastulatum|FBAQU556-10|I|Germany|Bavaria|658[0n]
Coenagrion mercuriale|GBMIX947-14||Germany|Bavaria|658[0n]
Coenagrion mercuriale|FBAQU1429-13|adult|Germany|Bavaria|658[0n]
Coenagrion scitulum|EDF007-18|adult|Austria|Lower Austria|658[0n]
Coenagrion scitulum|EDF008-18|adult|Austria|Lower Austria|658[0n]
Coenagrion scitulum|GBEPT888-14|Imago|Greece||658[0n]
Coenagrion scitulum|GBEPT907-14|Imago|Austria|Burgenland|658[0n]
Coenagrion caerulescens|ZPLOD197-20|Adult|Italy|Piedmont|658[0n]
Coenagrion caerulescens|ZPLOD196-20|Adult|Italy|Piedmont|658[0n]
Coenagrion caerulescens|ZPLOD194-20|Adult|Italy|Piedmont|658[0n]
Coenagrion caerulescens|ZPLOD195-20|Adult|Italy|Piedmont|658[0n]
Coenagrion caerulescens|ZPLOD190-20|Adult|Italy|Sardinia|658[0n]
Coenagrion caerulescens|ZPLOD192-20|Adult|Italy|Apulia|658[0n]
Coenagrion caerulescens|ZPLOD198-20|Adult|Italy|Molise|658[0n]
Coenagrion caerulescens|ZPLOD189-20|Adult|Italy|Sicily|658[0n]
Enallagma cyathigerum|GBODO072-18|a|Germany|Bavaria|577[0n]
Enallagma cyathigerum|ODTRI011-14|adult|Norway|Nord-Trondelag|602[0n]
Enallagma cyathigerum|ODTRI012-14|adult|Norway|Nord-Trondelag|611[0n]
Enallagma cyathigerum|GBODO052-18|a|Germany|Bavaria|658[2n]
Enallagma cyathigerum|HETFI052-11|A|Norway|Finnmark|658[0n]
Enallagma cyathigerum|HETFI049-11|A|Norway|Finnmark|658[0n]
Enallagma cyathigerum|GBODO024-18|a|Germany|Bavaria|658[1n]
Enallagma cyathigerum|FBAQU490-10|A|Germany|Bavaria|616[0n]
Enallagma cyathigerum|ZMBN960-17|immature|Norway|Hordaland|658[0n]
Enallagma cyathigerum|GBODO172-18|a|Germany|Bavaria|658[0n]
Enallagma cyathigerum|TRDOD073-14|adult|Norway|Nord-Trondelag|658[0n]
Enallagma cyathigerum|ODOPL101-19|larvae|Poland||634[0n]
Enallagma cyathigerum|GBODO051-18|a|Germany|Bavaria|511[3n]
Enallagma cyathigerum|FBAQU532-10|A|Germany|Bavaria|658[0n]
Enallagma cyathigerum|FBAQU015-09|I|Germany|Bavaria|644[0n]
Enallagma cyathigerum|GODO011-18||Germany|Mecklenburg-Vorpommern|658[0n]
Enallagma cyathigerum|ODOPL126-19|larvae|Poland||640[0n]
Enallagma cyathigerum|GODO012-18||Germany|Mecklenburg-Vorpommern|658[0n]
Enallagma cyathigerum|GODO013-18||Germany|Mecklenburg-Vorpommern|658[0n]
Enallagma cyathigerum|GODO014-18||Germany|Mecklenburg-Vorpommern|658[0n]
Enallagma cyathigerum|GBEPT912-14|Imago|Germany|Bavaria|658[0n]
Enallagma cyathigerum|PLSW072-20|imago|Poland||658[0n]
Enallagma cyathigerum|FBAQU313-09|A|Germany|Bavaria|658[0n]
Enallagma cyathigerum|GBODO181-18|a|Germany|Bavaria|658[0n]
Enallagma cyathigerum|GBODO100-18|a|Germany|Bavaria|658[0n]
Enallagma cyathigerum|TRDOD024-14|adult|Norway|Nord-Trondelag|658[0n]
Enallagma cyathigerum|TRDOD072-14|adult|Norway|Sor-Trondelag|658[0n]
Enallagma cyathigerum|GBODO054-18|a|Germany|Bavaria|658[3n]
Enallagma cyathigerum|GBODO040-18|a|Germany|Bavaria|658[2n]
Enallagma cyathigerum|GBODO076-18|a|Germany|Bavaria|658[1n]
Enallagma cyathigerum|GBODO096-18|a|Germany|Bavaria|658[2n]
Enallagma cyathigerum|GBODO050-18|a|Germany|Bavaria|567[2n]
Enallagma cyathigerum|PLSW073-20|imago|Poland||633[0n]
Enallagma cyathigerum|ODOPL100-19|larvae|Poland||636[0n]
Enallagma cyathigerum|FBAQU1504-13|adult|Germany|Brandenburg|658[0n]
Enallagma cyathigerum|PLSW016-20|imago|Poland||658[0n]
Ischnura pumilio|FBAQU315-09|A|Germany|Bavaria|658[0n]
Ischnura pumilio|FBAQU496-10|A|Germany|Bavaria|614[0n]
Ischnura pumilio|FBAQU538-10|A|Germany|Bavaria|614[0n]
Ischnura pumilio|FBAQU537-10|A|Germany|Bavaria|521[0n]
Ischnura genei|ZPLOD454-20|Adult|Italy|Sicily|658[0n]
Ischnura genei|ZPLOD457-20|Adult|Italy|Sicily|658[0n]
Ischnura elegans|TRDOD027-14|adult|Norway|Nord-Trondelag|658[0n]
Ischnura elegans|TRDOD088-14|adult|Norway|Nord-Trondelag|658[0n]
Ischnura elegans|TRDOD089-14|adult|Norway|Nord-Trondelag|658[0n]
Ischnura elegans|TRDOD090-14|adult|Norway|Nord-Trondelag|658[0n]
Ischnura saharensis|ZPLOD845-20|Adult|Morocco||658[0n]
Ischnura genei|ZPLOD452-20|Adult|Italy|Sardinia|658[0n]
Ischnura elegans|ODOPL015-18|larvae|Poland||626[0n]
Ischnura elegans|ODOPL087-19|larvae|Poland||633[0n]
Ischnura elegans|GBODO131-18|a|Germany|Bavaria|658[1n]
Ischnura elegans|GBEPT940-14|Imago|Germany|Bavaria|658[0n]
Ischnura elegans|GBODO084-18|a|Germany|Bavaria|658[0n]
Ischnura elegans|GBUPS193-14|adult|Germany|Bavaria|683[1n]
Ischnura elegans|GBUPS201-14|adult|Germany|Bavaria|646[0n]
Ischnura elegans|ODOPL089-19|larvae|Poland||636[0n]
Ischnura elegans|ODOPL088-19|larvae|Poland||636[0n]
Ischnura elegans|ODOPL028-19|larvae|Poland||641[0n]
Ischnura elegans|ODOPL026-19|larvae|Poland||638[0n]
Ischnura elegans|GBUPS205-14|adult|Germany|Bavaria|574[0n]
Ischnura elegans|ODOPL059-19|larvae|Poland||633[0n]
Ischnura elegans|ODOPL060-19|larvae|Poland||633[0n]
Ischnura elegans|GBUPS191-14|adult|Germany|Bavaria|684[0n]
Ischnura elegans|GBUPS195-14|adult|Germany|Bavaria|668[0n]
Ischnura elegans|GODO016-18||Germany|Mecklenburg-Vorpommern|658[0n]
Ischnura elegans|PLSW013-20|imago|Poland||658[0n]
Ischnura saharensis|ZPLOD844-20|Adult|Morocco||658[0n]
Ischnura elegans|FBAQU494-10|A|Germany|Bavaria|658[0n]
Ischnura elegans|GBEPT890-14|Imago|Greece||658[0n]
Ischnura elegans|GBEPT891-14|Imago|Greece||658[0n]
Ischnura elegans|ODOPL014-18|larvae|Poland||627[0n]
Ischnura elegans|ODOPL016-18|larvae|Poland||627[0n]
Ischnura elegans|ODOPL013-18|larvae|Poland||626[0n]
Ischnura elegans|ODOPL061-19|larvae|Poland||630[0n]
Ischnura elegans|ODOPL048-19|larvae|Poland||633[0n]
Ischnura elegans|GBEPT938-14|Imago|Germany|Bavaria|658[0n]
Ischnura elegans|GBEPT943-14|Imago|Austria|Burgenland|658[0n]
Ischnura elegans|GBODO063-18|a|Germany|Bavaria|658[2n]
Ischnura elegans|FBAQU172-09|A|Germany|Bavaria|654[0n]
Ischnura elegans|ODOPL004-18|larvae|Poland||558[0n]
Ischnura elegans|GBODO061-18|a|Germany|Bavaria|658[1n]
Ischnura elegans|ODOPL049-19|larvae|Poland||629[0n]
Ischnura elegans|ODOPL027-19|larvae|Poland||630[0n]
Ischnura elegans|GBODO021-18|a|Germany|Bavaria|658[1n]
Ischnura elegans|GBUPS194-14|adult|Germany|Bavaria|657[1n]
Ischnura elegans|GBUPS202-14|adult|Germany|Bavaria|674[0n]
Ischnura elegans|GBUPS197-14|adult|Germany|Bavaria|669[0n]
Ischnura elegans|FBAQU536-10|A|Germany|Bavaria|588[0n]

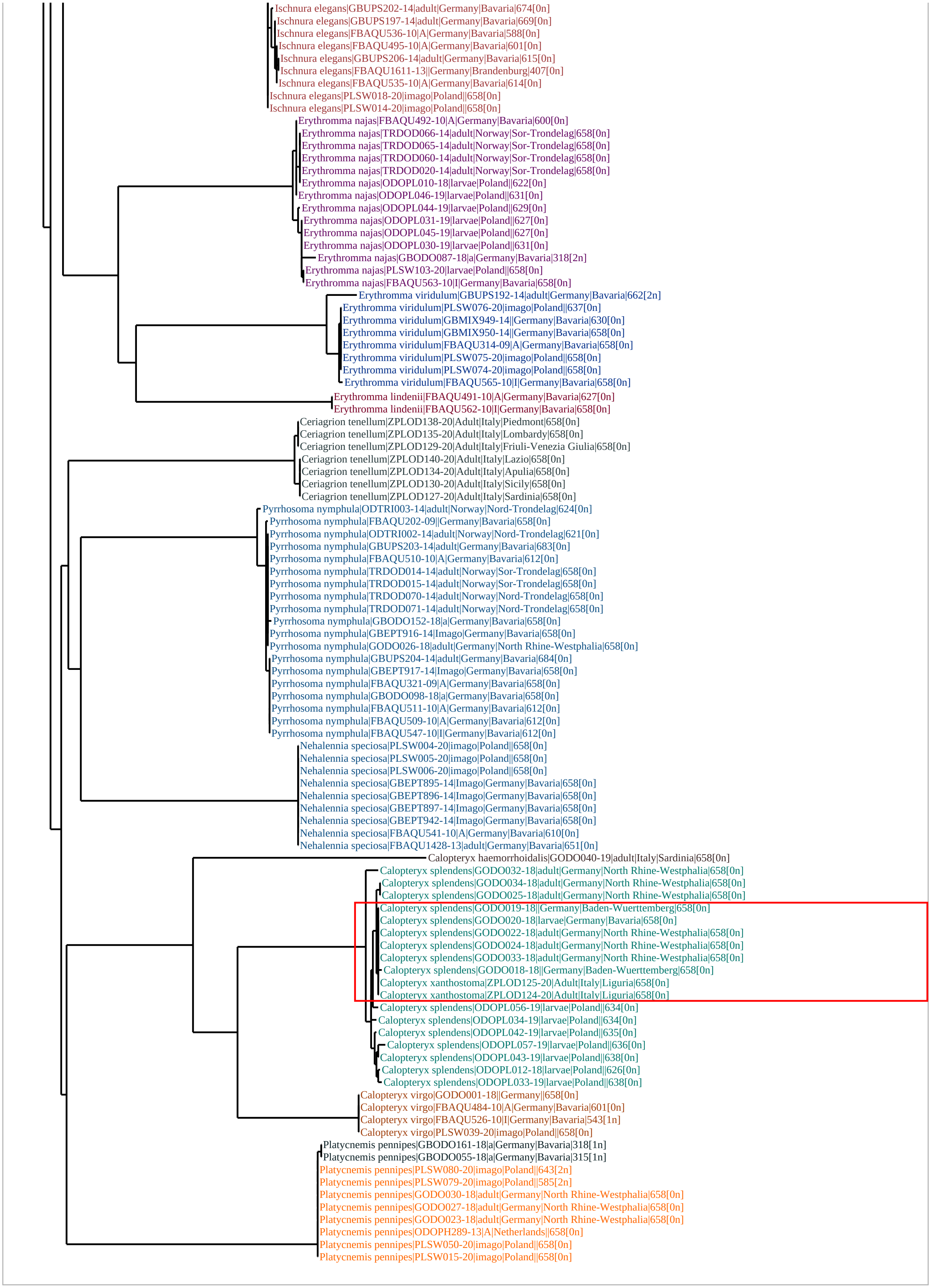

Ischnura elegans|GBUPS202-14|adult|Germany|Bavaria|674[0n]
Ischnura elegans|GBUPS197-14|adult|Germany|Bavaria|669[0n]
Ischnura elegans|FBAQU536-10|A|Germany|Bavaria|588[0n]
Ischnura elegans|FBAQU495-10|A|Germany|Bavaria|601[0n]
Ischnura elegans|GBUPS206-14|adult|Germany|Bavaria|615[0n]
Ischnura elegans|FBAQU1611-13||Germany|Brandenburg|407[0n]
Ischnura elegans|FBAQU535-10|A|Germany|Bavaria|614[0n]
Ischnura elegans|PLSW018-20|imago|Poland||658[0n]
Ischnura elegans|PLSW014-20|imago|Poland||658[0n]
Erythromma najas|FBAQU492-10|A|Germany|Bavaria|600[0n]
Erythromma najas|TRDOD066-14|adult|Norway|Sor-Trondelag|658[0n]
Erythromma najas|TRDOD065-14|adult|Norway|Sor-Trondelag|658[0n]
Erythromma najas|TRDOD060-14|adult|Norway|Sor-Trondelag|658[0n]
Erythromma najas|TRDOD020-14|adult|Norway|Sor-Trondelag|658[0n]
Erythromma najas|ODOPL010-18|larvae|Poland||622[0n]
Erythromma najas|ODOPL046-19|larvae|Poland||631[0n]
Erythromma najas|ODOPL044-19|larvae|Poland||629[0n]
Erythromma najas|ODOPL031-19|larvae|Poland||627[0n]
Erythromma najas|ODOPL045-19|larvae|Poland||627[0n]
Erythromma najas|ODOPL030-19|larvae|Poland||631[0n]
Erythromma najas|GBODO087-18|a|Germany|Bavaria|318[2n]
Erythromma najas|PLSW103-20|larvae|Poland||658[0n]
Erythromma najas|FBAQU563-10|I|Germany|Bavaria|658[0n]
Erythromma viridulum|GBUPS192-14|adult|Germany|Bavaria|662[2n]
Erythromma viridulum|PLSW076-20|imago|Poland||637[0n]
Erythromma viridulum|GBMIX949-14||Germany|Bavaria|630[0n]
Erythromma viridulum|GBMIX950-14||Germany|Bavaria|658[0n]
Erythromma viridulum|FBAQU314-09|A|Germany|Bavaria|658[0n]
Erythromma viridulum|PLSW075-20|imago|Poland||658[0n]
Erythromma viridulum|PLSW074-20|imago|Poland||658[0n]
Erythromma viridulum|FBAQU565-10|I|Germany|Bavaria|658[0n]
Erythromma lindenii|FBAQU491-10|A|Germany|Bavaria|627[0n]
Erythromma lindenii|FBAQU562-10|I|Germany|Bavaria|658[0n]
Ceriagrion tenellum|ZPLOD138-20|Adult|Italy|Piedmont|658[0n]
Ceriagrion tenellum|ZPLOD135-20|Adult|Italy|Lombardy|658[0n]
Ceriagrion tenellum|ZPLOD129-20|Adult|Italy|Friuli-Venezia Giulia|658[0n]
Ceriagrion tenellum|ZPLOD140-20|Adult|Italy|Lazio|658[0n]
Ceriagrion tenellum|ZPLOD134-20|Adult|Italy|Apulia|658[0n]
Ceriagrion tenellum|ZPLOD130-20|Adult|Italy|Sicily|658[0n]
Ceriagrion tenellum|ZPLOD127-20|Adult|Italy|Sardinia|658[0n]
Pyrrhosoma nymphula|ODTRI003-14|adult|Norway|Nord-Trondelag|624[0n]
Pyrrhosoma nymphula|FBAQU202-09||Germany|Bavaria|658[0n]
Pyrrhosoma nymphula|ODTRI002-14|adult|Norway|Nord-Trondelag|621[0n]
Pyrrhosoma nymphula|GBUPS203-14|adult|Germany|Bavaria|683[0n]
Pyrrhosoma nymphula|FBAQU510-10|A|Germany|Bavaria|612[0n]
Pyrrhosoma nymphula|TRDOD014-14|adult|Norway|Sor-Trondelag|658[0n]
Pyrrhosoma nymphula|TRDOD015-14|adult|Norway|Sor-Trondelag|658[0n]
Pyrrhosoma nymphula|TRDOD070-14|adult|Norway|Nord-Trondelag|658[0n]
Pyrrhosoma nymphula|TRDOD071-14|adult|Norway|Nord-Trondelag|658[0n]
Pyrrhosoma nymphula|GBODO152-18|a|Germany|Bavaria|658[0n]
Pyrrhosoma nymphula|GBEPT916-14|Imago|Germany|Bavaria|658[0n]
Pyrrhosoma nymphula|GODO026-18|adult|Germany|North Rhine-Westphalia|658[0n]
Pyrrhosoma nymphula|GBUPS204-14|adult|Germany|Bavaria|684[0n]
Pyrrhosoma nymphula|GBEPT917-14|Imago|Germany|Bavaria|658[0n]
Pyrrhosoma nymphula|FBAQU321-09|A|Germany|Bavaria|658[0n]
Pyrrhosoma nymphula|GBODO098-18|a|Germany|Bavaria|658[0n]
Pyrrhosoma nymphula|FBAQU511-10|A|Germany|Bavaria|612[0n]
Pyrrhosoma nymphula|FBAQU509-10|A|Germany|Bavaria|612[0n]
Pyrrhosoma nymphula|FBAQU547-10|I|Germany|Bavaria|612[0n]
Nehalennia speciosa|PLSW004-20|imago|Poland||658[0n]
Nehalennia speciosa|PLSW005-20|imago|Poland||658[0n]
Nehalennia speciosa|PLSW006-20|imago|Poland||658[0n]
Nehalennia speciosa|GBEPT895-14|Imago|Germany|Bavaria|658[0n]
Nehalennia speciosa|GBEPT896-14|Imago|Germany|Bavaria|658[0n]
Nehalennia speciosa|GBEPT897-14|Imago|Germany|Bavaria|658[0n]
Nehalennia speciosa|GBEPT942-14|Imago|Germany|Bavaria|658[0n]
Nehalennia speciosa|FBAQU541-10|A|Germany|Bavaria|610[0n]
Nehalennia speciosa|FBAQU1428-13|adult|Germany|Bavaria|651[0n]
Calopteryx haemorrhoidalis|GODO040-19|adult|Italy|Sardinia|658[0n]
Calopteryx splendens|GODO032-18|adult|Germany|North Rhine-Westphalia|658[0n]
Calopteryx splendens|GODO034-18|adult|Germany|North Rhine-Westphalia|658[0n]
Calopteryx splendens|GODO025-18|adult|Germany|North Rhine-Westphalia|658[0n]
Calopteryx splendens|GODO019-18||Germany|Baden-Wuerttemberg|658[0n]
Calopteryx splendens|GODO020-18|larvae|Germany|Bavaria|658[0n]
Calopteryx splendens|GODO022-18|adult|Germany|North Rhine-Westphalia|658[0n]
Calopteryx splendens|GODO024-18|adult|Germany|North Rhine-Westphalia|658[0n]
Calopteryx splendens|GODO033-18|adult|Germany|North Rhine-Westphalia|658[0n]
Calopteryx splendens|GODO018-18||Germany|Baden-Wuerttemberg|658[0n]
Calopteryx xanthostoma|ZPLOD125-20|Adult|Italy|Liguria|658[0n]
Calopteryx xanthostoma|ZPLOD124-20|Adult|Italy|Liguria|658[0n]
Calopteryx splendens|ODOPL056-19|larvae|Poland||634[0n]
Calopteryx splendens|ODOPL034-19|larvae|Poland||634[0n]
Calopteryx splendens|ODOPL042-19|larvae|Poland||635[0n]
Calopteryx splendens|ODOPL057-19|larvae|Poland||636[0n]
Calopteryx splendens|ODOPL043-19|larvae|Poland||638[0n]
Calopteryx splendens|ODOPL012-18|larvae|Poland||626[0n]
Calopteryx splendens|ODOPL033-19|larvae|Poland||638[0n]
Calopteryx virgo|GODO001-18||Germany||658[0n]
Calopteryx virgo|FBAQU484-10|A|Germany|Bavaria|601[0n]
Calopteryx virgo|FBAQU526-10|I|Germany|Bavaria|543[1n]
Calopteryx virgo|PLSW039-20|imago|Poland||658[0n]
Platycnemis pennipes|GBODO161-18|a|Germany|Bavaria|318[1n]
Platycnemis pennipes|GBODO055-18|a|Germany|Bavaria|315[1n]
Platycnemis pennipes|PLSW080-20|imago|Poland||643[2n]
Platycnemis pennipes|PLSW079-20|imago|Poland||585[2n]
Platycnemis pennipes|GODO030-18|adult|Germany|North Rhine-Westphalia|658[0n]
Platycnemis pennipes|GODO027-18|adult|Germany|North Rhine-Westphalia|658[0n]
Platycnemis pennipes|GODO023-18|adult|Germany|North Rhine-Westphalia|658[0n]
Platycnemis pennipes|ODOPH289-13|A|Netherlands||658[0n]
Platycnemis pennipes|PLSW050-20|imago|Poland||658[0n]
Platycnemis pennipes|PLSW015-20|imago|Poland||658[0n]
