# Supplementary material for: Coverage and quality of DNA barcode references for Central and Northern European Odonata
Source: PeerJ. 2021 May 3;9:e11192. doi: 10.7717/peerj.11192 (PMC8101477; doi:10.7717/peerj.11192)
Supplement: Supplemental Information 4 [file peerj-09-11192-s004.pdf]

Institute (sample origin)	Institution (processing)	PCR primers	References	Comment
NHMW, Austria	NHMW	Tyr-Odo-F (5’ – CTC CTA TAT AGA TTT ACA GTC T)<br>Leu-Odo-R (5' – CTT AAA TCC ATT GCA CTT TTC TGC C)	Haring et al. 2020	COI plus partial sections of the flanking tRNA gene
NHMW, Austria	NHMW	CO1-Odo-F5 (5’ – TGC GAC RA TGR CTG TTT TC)<br>CO1-Odo-R6 (5’ – TGC ACT TTT CTG CCA CAT TAA A)	Haring et al. 2020	COI plus partial sections of the flanking tRNA gene
NorBOL, Norway	CCDB	CLepFolF (5’ – ATT CAA CCA ATC ATA AAG ATA TTG G)<br>CLepFolR (5’ – TAA ACT TCT GGA TGT CCA AAA AAT CA)	Ivanova et al. 2006 and Hernández-Triana et al. 2014	
KFUG, Austria	KFUG	ODO_LCO1490d (5’ – TTT CTA CWA ACC AYA AAG ATA TTG G)<br>ODO_HCO2198d (5’ – TAA ACT TCW GGR TGT CCA AAR AAT CA)	Dijkstra et al. 2014	
SNSB-ZSM, Germany	CCDB	CLepFolF (5’ – ATT CAA CCA ATC ATA AAG ATA TTG G)<br>CLepFolR (5’ – TAA ACT TCT GGA TGT CCA AAA AAT CA)	Ivanova et al. 2006 and Hernández-Triana et al., 2014	
SNSB-ZSM, Germany	CCDB	dgHCO2198 (5' – TAA ACT TCA GGG TGA CCA AAR AAY CA)<br>mlCOIintF (5' – GGW ACW GGW TGA ACW GTW TAY CCY CC)	Meyer 2003 and Leray et al. 2013	mini barcodes
University of Lodz, Poland	CCDB	OdoF1_t1 (5’ – TGT AAA ACG ACG GCC AGT ATT CAA CHA ATC ATA ARG ATA TTG G)<br>OdoR1_t1 (5’ – CAG GAA ACA GCT ATG ACT AAA CTT CTG GAT GYC CRA ARA AYC A)	Semotok, unpublished, Source: BOLD Systems primer database; Galimberti et al. 2020; Rewicz et al. 2020	includes M13 sequencing tails
University of Milano-Bicocca and private collection G. Assandri	University of Milano-Bicocca	OdoF1_t1 (5’ – TGT AAA ACG ACG GCC AGT ATT CAA CHA ATC ATA ARG ATA TTG G)<br>OdoR1_t1 (5’ – CAG GAA ACA GCT ATG ACT AAA CTT CTG GAT GYC CRA ARA AYC A)	Semotok, unpublished, Source: BOLD Systems primer database; Galimberti et al. 2020; Rewicz et al. 2020	includes M13 sequencing tails
University of Milano-Bicocca and private collection G. Assandri	University of Milano-Bicocca	ODO_LCO1490d (5’ – TTT CTA CWA ACC AYA AAG ATA TTG G)<br>ODO_HCO2198d (5’ – TAA ACT TCW GGR TGT CCA AAR AAT CA)	Dijkstra et al. 2014	
ZFMK, Germany	GBOL at ZFMK	LCO1490-JJ (5' – CHA CWA AYC ATA AAG ATA TYG G)<br>HCO2198-JJ 5' – AWA CTT CVG GRT GVC CAA ARA ATC A)	Astrin et al. 2016	
